# Critical analysis of the limitations of Bleaney's theory of magnetic anisotropy in paramagnetic lanthanide coordination complexes†
†Electronic supplementary information (ESI) available: Synthesis and characterisation of selected complexes, NMR spectral figures, tables of shift (including *T* dependence) and relaxation rate data at 5 magnetic field strengths. The X-ray structure of [Tm.gDOTA(H_2_O)]is available. CCDC 1032378. For ESI and crystallographic data in CIF or other electronic format see DOI: 10.1039/c4sc03429e


**DOI:** 10.1039/c4sc03429e

**Published:** 2014-12-17

**Authors:** Alexander M. Funk, Katie-Louise N. A. Finney, Peter Harvey, Alan M. Kenwright, Emily R. Neil, Nicola J. Rogers, P. Kanthi Senanayake, David Parker

**Affiliations:** a Department of Chemistry , Durham University , South Road , Durham , DH1 3LE , UK . Email: david.parker@dur.ac.uk

## Abstract

The origins of the breakdown of Bleaney's theory of magnetic anisotropy are described, based on an analysis of eleven different complexes of the second half of the 4f elements that form isostructural series. An examination of the chemical shift and relaxation rate behaviour of resonances located at least four bonds away from the paramagnetic centre was undertaken, and correlated to theoretical predictions. The key limitations relate to comparability of ligand field splitting with spin–orbit coupling, variation in the position of the principal magnetic axis between Ln complexes and the importance of multipolar terms in describing lanthanide ligand field interactions.

## Abstract 2

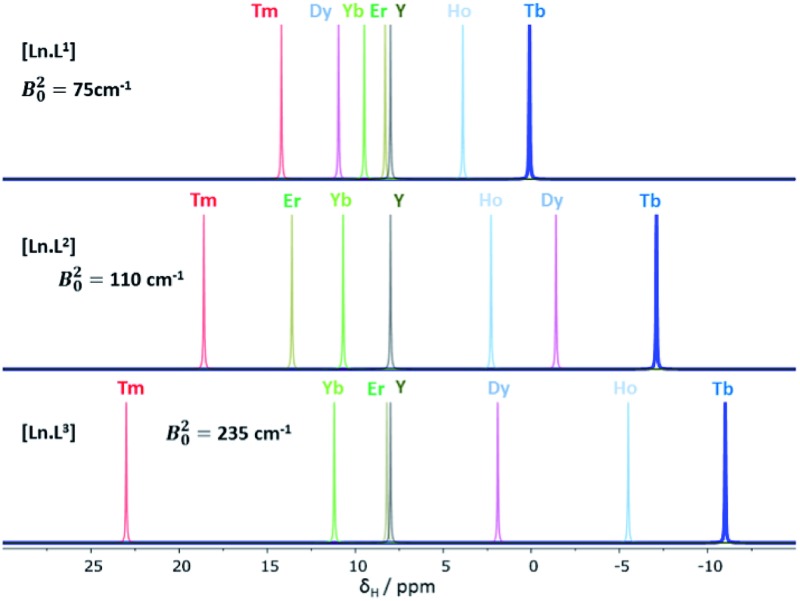
The origins of the breakdown of Bleaney's theory of magnetic anisotropy are described, based on an analysis of eleven different complexes of the second half of the 4f elements that form isostructural series.

## Introduction

We describe experimental evidence showing that Bleaney's theory of magnetic anisotropy has severe limitations. The origins of the breakdown are traced and the implications assessed for the design of paramagnetic probes in magnetic resonance.

Bleaney's theory of magnetic anisotropy^1^ has been a key reference point for over 40 years, when considering the chemical shift of NMR resonances that are at least four bonds from a paramagnetic lanthanide centre. Under these conditions, any contact contribution to the shift is usually very small and the measured paramagnetic shift is predominantly a pseudocontact shift (*δ*_p_)^1^,^2^ that can be defined in terms of the geometric coordinates, the ligand field, temperature and the nature of the lanthanide ion, eqn (1) to (2),1
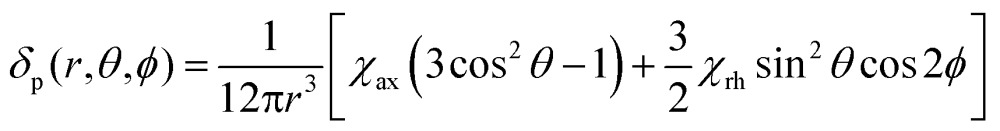

2
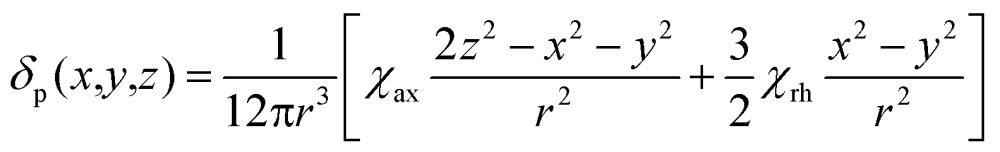
where {*r*,*θ*,*φ*} are spherical coordinates of the observed nucleus, *χ*_ax_ is the axiality of the electron magnetic susceptibility tensor, *χ*_rh_ is its rhombicity, in which the coordinate system is aligned to the eigen system of the susceptibility tensor, with the electron located at the origin. These equations are often expressed (eqn (3)) with reference to the principal magnetic axes system, highlighting the strong directional dependence of the pseudocontact shift, and its link to the Bleaney constant, *C*_*J*_ (eqn (4)).3
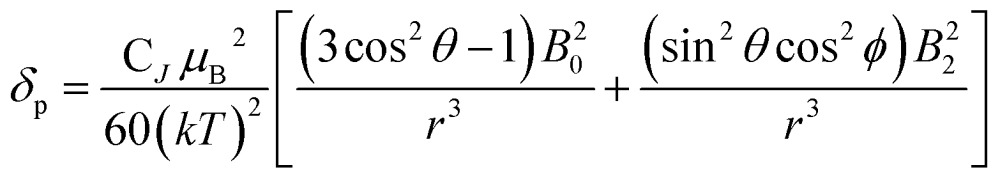

4 *C*_*J*_ = *g*^2^*J*(*J* + 1)(2*J* – 1)(2*J* + 3)*J*|*α*|*J*

where *C*_*J*_ is characteristic of the Ln(iii) ion, *θ* and *φ* are the angles between the nucleus under consideration and the principal magnetic axis of the lanthanide ion, *g* is the Landé factor and *μ*_B_ is the Bohr magneton. The *B* parameters are second order ligand field terms, determined primarily by local symmetry and donor atom polarisability.

## Limitations of Bleaney theory

There are several assumptions in this theory that need scrutinising. First, Bleaney assumed that the ligand field splitting is much less than *kT* (205 cm^–1^ at 298 K). Generally, this is not the case. Values for *B*20 of between 80 and 1500 cm^–1^ have been established,^3^ with the majority of coordination complexes having *B*20 values of more than two times *kT*. The theory ignores the contribution of higher order crystal field terms that may play an important role in determining the overall ligand field, especially in low symmetry systems. This aspect has been addressed in part by Golding in detailed mathematical analyses that lack physicochemical relevance.^4^ Second, it is assumed that the electron is a point charge at the coordinate origin, and that its relaxation is instantaneous. This is evidently also not true, and consideration of f electron density probability functions suggest that a distributed model may be more apt. Such an approach has been put forward recently by Kuprov, in an important step.^5^ Furthermore, models of f electron distributions, highlighted by Long,^6^ show how certain ions (*e.g.* Eu, Yb, Tm and Er) possess a prolate f electron density distribution, whilst others (*e.g.* Ce, Pr, Tb, Dy) are oblate. Such behaviour correlates with the differing sense of shift, incorporated in the Bleaney constant, *C*_*J*_. Third, it is assumed that the position of the principal magnetic axis does not vary as the lanthanide ion changes in an isostructural series of complexes. However, Sessoli has recently demonstrated that in the solid-state at very low temperature, the principal (easy) axis of magnetisation in the *C*_4_ symmetric complexes of [Ln.DOTA(H_2_O)] (DOTA = 1,4,7,10-cyclododecane-tetracetate), changes position as the Ln series is traversed.^7^,^8^ It rotates by up to 90° from Tb to Yb, for example, and aligns approximately with the molecular *C*_4_ axis only for those ions that have a prolate f electron distribution, *i.e.* Yb, Tm and Eu.

Finally, in devising the Bleaney constants (*C*_*J*_), it is implicitly assumed that *J* is a good quantum number that defines the spin–orbit coupling. However, the Russell–Saunders coupling scheme may not be appropriate for these relatively heavy elements and the values of the spin–orbit coupling energies (typically of the order of 650 to 1800 cm^–1^), are not much bigger than the overall ligand field splitting terms in some cases, in complexes where the ligand field is large. In this situation, the concept of ‘*J* mixing’ is often invoked,^9^,^10^ as a means of correcting for, or simply recognising imprecision in *J*. The measurements of Sessoli were carried out in the solid state at cryogenic temperatures. Under these conditions, the orientation of the principal axis of the magnetic anisotropy relative to the molecular symmetry axis should not be regarded as breaking the primacy of the molecular symmetry axis in solution at room temperature. Sessoli argues that the orientation of the principal axis, in the plane perpendicular to the molecular symmetry axis, changes by up to 90° according to whether a water molecule occupies the axial coordination site, (and exchange of water at this site is known to be rapid in aqueous solution at room temperature, which tends to average any anisotropy in the plane perpendicular to the molecular symmetry axis). Furthermore, rapid isotropic molecular tumbling in solution at room temperature also tends to average anisotropy perpendicular to the molecular symmetry axis. Since the principal magnetic axis corresponds to the largest Cartesian component of the anisotropy tensor, in the presence of averaging in the plane perpendicular to the molecular symmetry axis, a principal axis perpendicular to the molecular symmetry axis corresponds to an oblate tensor while a principal axis collinear with the molecular symmetry axis corresponds to a prolate tensor. Indeed, it seems likely that Sessoli and Long report mutually consistent conclusions, expressed in rather different terms. It is clear from the number of ^1^H NMR signals observed for the symmetric complexes that they have effectively zero anisotropy, on the relevant timescale, in the plane perpendicular to the molecular symmetry axis in solution at room temperature. The same averaging does not apply to non-symmetric complexes.

The limitations of Bleaney theory have been expressed previously,^1^,^2^ notably by Binnemans.^10^,^11^ He examined the theoretical impact of distortion from rotational symmetry on the local magnetic anisotropy in selected model systems, and suggested that the effect could be significant.

## Results and discussion

**Scheme 1:**
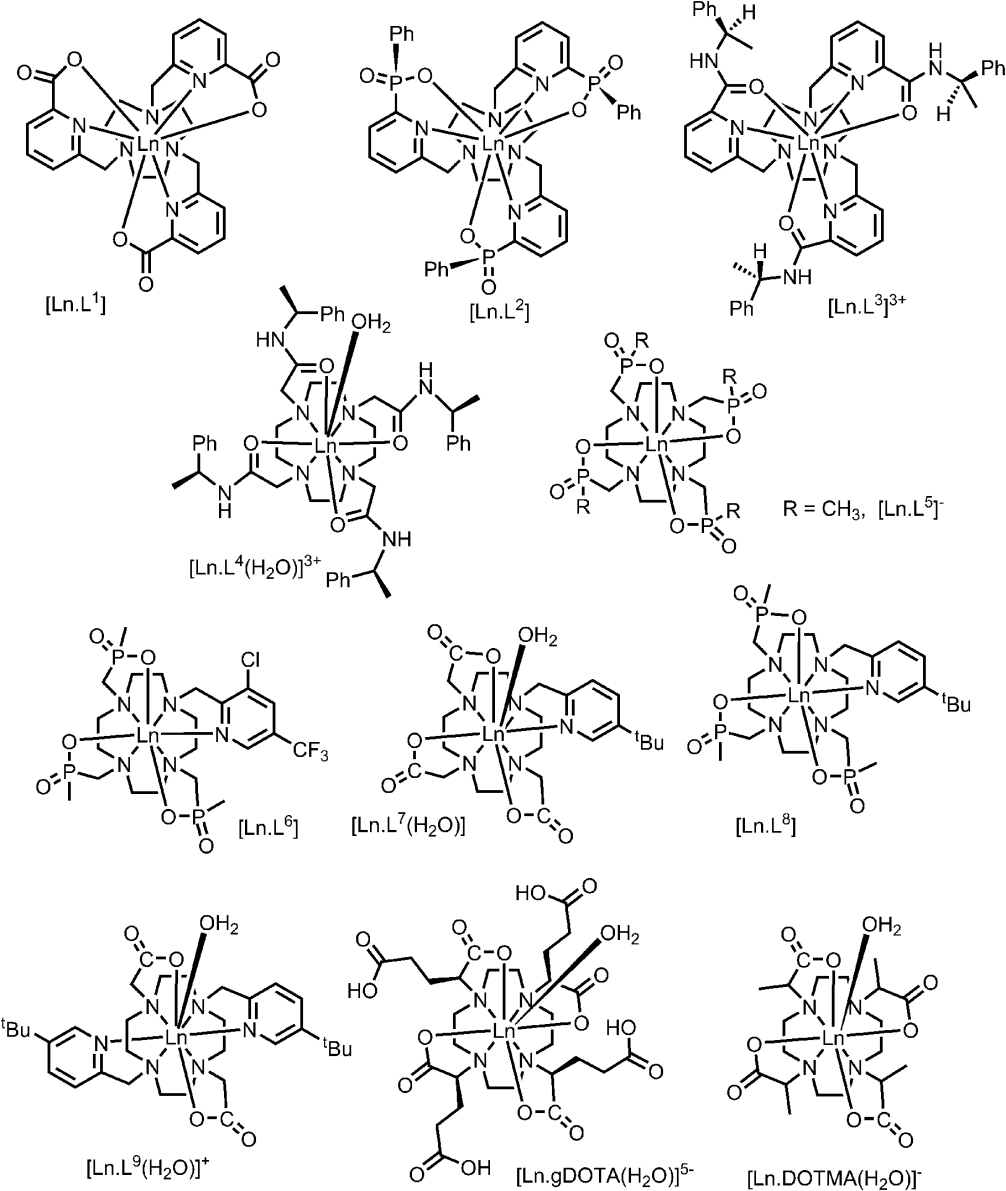


A systematic analysis was undertaken of shift and relaxation rate data for lanthanide(iii) complexes that have been shown to form an isostructural series from Tb to Yb. The resonances analysed were separated by at least four bonds from the lanthanide ion, in order to minimise any contribution arising from a contact shift.1*d* In all, eleven different systems were studied, ranging from three complexes possessing a time-averaged *C*_3_ axis of symmetry [Ln.L^1–3^]^12^–^14^ certain systems with average *C*_4_ symmetry *e.g.*, [Ln.L^4^(H_2_O)]^3+^,^15^ [Ln.L^5^]^–^,^16^ (plus selected complexes of [Ln.gDOTA(H_2_O)]^5–^ and [Ln.DOTMA(H_2_O)]^–^ that themselves do not form an isostructural series, yet whose hydration state and degree of twist is established),^17^,^18^ to a set of lower symmetry cyclen complexes, which divide into 9-coordinate carboxylate and 8-coordinate phosphinate examples,^19^,^20^ (Scheme 1). For each of these examples, the value of the second order crystal field term *B*20 was estimated by analysis of the splitting of the Δ*J* = 1 band in the emission spectrum of the corresponding Eu(iii) complex, following established methods.^21^

The values of crystal field coefficients are generally considered to decrease only slightly across the lanthanide series, for complexes in a common coordination environment. The values of *B*22 are generally smaller than those for *B*20 and are not so readily obtained by spectral analysis.^22^

### Shift behaviour of the *C*_3_ symmetric series, [Ln.L^1–3^]

The three complexes, [Ln.L^1–3^], differ in the nature of the oxygen donor. The ligand field in these systems is comparatively small and is less than [Ln.L^1–2^] or of the same order as *kT* [Ln.L^3^]^3+^. Due to their *C*_3_ symmetry, the second order crystal field splitting parameter, *B*20 should characterise the overall crystal field splitting dependence of each complex, according to Bleaney theory. Assignments of each ligand proton NMR resonance have been reported earlier and were verified by measuring the rate of relaxation of each resonance at five different magnetic fields (4.7 to 16.5 T).^12^–^14^ This data set also allowed the distance of each resonance from the paramagnetic centre in the solution state to be estimated and compared to that established by the X-ray structural analyses, reported in each case. Intramolecular nuclear relaxation rate data is most commonly analysed using Bloch–Redfield–Wangsness theory. The paramagnetic relaxation arises from rotational and conformational modulation of the electron–nuclear dipolar interaction, eqn (5) and (6).5
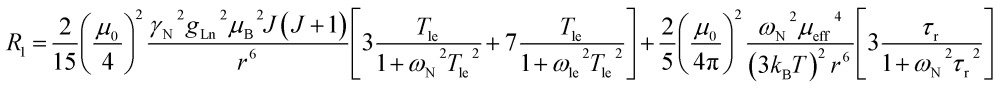

6
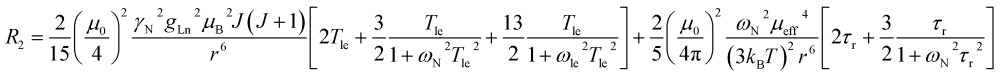
where *μ*_0_ is the vacuum permeability, *γ*_N_ is the gyromagnetic ratio of the nucleus, *g*_Ln_ is the Landé factor of the fundamental multiplet *J* of the free Ln^3+^ ion, *μ*_B_ is the Bohr magneton (BM), *r* is the electron–nuclear distance, *τ*_r_ is the rotational correlation time, *ω*_N_ is the nuclear Larmor frequency, *ω*_e_ is the electron Larmor frequency, (*μ*_eff_)^2^ is the square of the effective magnetic moment and *T*_1e_ is the longitudinal relaxation time of the electron spin. The dependence of *R*_1_ on (*μ*_eff_)^4^ and (*ω*_N_)^2^ in the second parts of eqn (5) and (6) (Curie term) become increasingly important at higher magnetic fields, for ions with larger values of *μ*_eff_, *e.g.* Ho, Dy, Tb and Er.

These equations are also based on certain assumptions. First, the point-dipole approximation is assumed and the dipolar and Curie contributions are treated as additive and ignore any cross-relaxation. The zero-field splitting (ZFS) of the energy levels is neglected, although Luchinat has proposed a modification to the dipolar term that gives weight to the size of the ZFS term, leading to an increase in *T*_1e_.^23^ Finally, the rotational correlation time, *τ*_r_, is treated as isotropic and is assumed to be the same for each resonance examined. This is evidently not true, as perfect motional coupling does not occur. The occurrence of localised rotational correlation times has been quantified by Szabo, for cases where the local motion of the atom or groups of atoms under consideration is not strongly coupled to the overall molecular tumbling rate.^24^

The variation of experimental relaxation rate data^12^ with field was used to estimate the Ln–proton average distance, *r*, and the complex rotational correlation time, *τ*_r_, using global minimisation methods^25^ for the six complexes (Tb–Yb) individually. Using literature values of *μ*_eff_, fits to eqn (5) were allowed to minimise, and converged to well-defined minima. The computed distances correlate well with those found by X-ray analysis, consistent with correct NMR assignments, (Table 2).

**Table 1:** Second order crystal field coefficients assessed by analysing the Δ*J* = 1 manifold in Eu(iii) emission spectrum (H_2_O, 295 K)

Complex	*B* 2 0 ^*a*^/cm^–1^
[Eu.L^1^]	+75
[Eu.L^2^]	+110
[Eu.L^3^]^3+^	+235
[Eu.L^4^(H_2_O)]^3+^	–470
[Eu.L^5^]^–^	–700
[Eu.L^6^]	–550
[Eu.L^7^(H_2_O)]	–455
[Eu.L^8^]	–570
[Eu.L^9^(H_2_O)]^+^	–355
[Eu.gDOTA(H_2_O)]^5–^	–700^*b*^

^*a*^Error estimated to be ±30 cm^–1^.

^*b*^The same value was found for [Eu.DOTMA(H_2_O)]^–^.

**Table 2:** Chemical shift data^*a*^ ^*b*^ ^*c*^ for pyridyl resonances (pyH^3,4^) in [Ln.L^1–3^] with estimated average internuclear distances, derived by single fitting analysis of NMR relaxation rate data (295 K, [Ln.L^1^] in D_2_O, [Ln.L^2,3^] in CD_3_OD) and compared to X-ray structural data (120 K)^12^–^14^

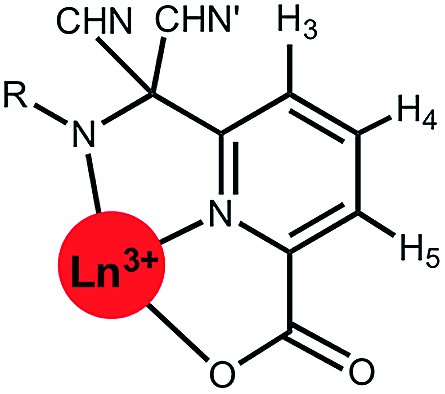						
Ln^3+^	*δ*_H_/ppm					
	pyH^3^			pyH^4^		
	[Ln.L^1^]	[Ln.L^2^]	[Ln.L^3^]^3+^	[Ln.L^1^]	[Ln.L^2^]	[Ln.L^3^]^3+^
Tb	0.1	–7.1	–11.0	4.9	–2.3	–3.2
Dy	9.4	–1.4	1.9	10.6	1.4	5.0
Ho	3.9	2.3	–5.5	6.2	4.1	–0.4
Er	8.3	13.6	8.2	7.9	11.9	7.9
Tm	14.2	18.6	23.0	13.5	16.4	19.6
Yb	9.5	10.7	11.6	9.1	10.3	11.2
Average *r*^*a*^/Å	5.56	5.71	5.50	6.28	6.58	6.46
X-ray *r*^*c*^/Å	5.40	5.53	5.48	6.22	6.36	6.26

^*a*^Averaged overall of the six lanthanide(iii) ions examined; the ionic radius of Ln^3+^ ions in 8 and 9 coordination contracts by 0.06 Å from Tb to Yb;

^*b*^for [Ln.L^1–3^], values rise from +75 to +110 and +235 cm^–1^ respectively.

^*c*^Magnetic susceptibilities used in the fitting analysis here: Tb (9.8); Dy (10.3); Ho (10.4); Er (9.4); Tm (7.6); Yb (4.3) BMA.

A general increase of the pseudocontact shift with increasing ligand field is evident from [Ln.L^1^] to [Ln.L^3^]^3+^. However, the order and strengths of the magnetic anisotropies show irregularities. In particular, Er(iii) stands out, due to the absence of a paramagnetic shift in both [Er.L^1^] and [Er.L^3^]^3+^. The complex [Dy.L^1^] also shows unexpected behaviour with a variable sign for the shift, not shown by the Tb analogue. This is the only case observed where the sign of a pseudocontact shift value does not follow the trend of the sign of the Bleaney constant. The chemical shifts of the pyH^3^ resonance do not follow the predicted values of magnetic anisotropy. The expected order of Dy(iii) > Tb(iii) ≫ Ho(iii) is not conserved in each series. Here, the Tb(iii) complexes give rise to the biggest pseudocontact shift and each Dy(iii) complex behaves differently. Even in these systems with a small ligand field, the order of magnetic anisotropy predicted by Bleaney's theory is not followed. The shift behaviour of pyH^4^ and pyH^5^ in these complexes was similar to pyH^3^ (ESI†).

The unusual shift behaviour here suggested the need to consider that there might be a significant contact shift for the pyH^3–5^ resonances, notwithstanding their 4 and 5-bond separations from the paramagnetic centre. Using the ‘two nuclei’ method devised by Reuben,1*e* plots of the paramagnetic shift of pyH^4^ divided by *S*_z_ *versus* pyH^3^/*S*_z_ gave linear correlations (ESI†) for each complex (*R*^2^ > 0.99) with intercepts of <0.1. These plots are independent of *C*_*J*_ and any crystal field term. The linear relationship confirms isostructurality, and the intercept gives a measure of the hyperfine coupling constant. The near-zero intercepts found (ESI,† pp. 24–27) are consistent with a very small contact shift contribution, as hypothesised above.

Binnemans^10^,^11^ suggested that the local magnetic anisotropy is modulated by the shape and degree of distortion of the coordination polyhedron in series of lanthanide(iii) complexes. An analysis of the twist angle of the mean plane of the 9N_3_ ring with reference to the three oxygen donor atoms in the X-ray structures of the *C*_3_ symmetric complexes ([Ln.L^1–3^]) was undertaken.^12^ No correlation between the twist angles (22 ± 2° in each system) and the measured magnetic anisotropy was found, indicating that polyhedral distortion does not seem to explain the observed shift variation in this case.

### Shift behaviour in *C*_4_-symmetric systems

The cationic lanthanide(iii) complexes of the tetra-amide ligand, L^4^, form an isostructural series across the f block, with each complex adopting a mono-capped square-antiprismatic structure with an axial water molecule.^15^ The paramagnetic shift, for Yb and Eu complexes, has been shown to be particularly sensitive to the polarisability of the capping donor ligand, giving rise to ^1^H NMR shift variations of up to 60 ppm for an axial ring proton in the Yb complex.^26^ Such magnetic anisotropy behaviour may be consistent with the hypothesis of the importance of the matching of a prolate f electron density distribution (*e.g.* Yb, Eu) with the ligand geometry, in defining the orientation of the principal magnetic axis.^7^ The other complexes in *C*_4_ symmetry that were analysed were the mono-aqua isomers of [Ln.DOTMA(H_2_O)]^–^ and the [Ln.gDOTA(H_2_O)]^5–^ analogues (Scheme 1) that exist in a capped square-antiprismatic coordination environment,^17^,^18^ and the eight-coordinate series of tetra-phosphinates, [Ln.L^5^]^–^, in which there is no bound water molecule and the twist angle about the *C*_4_ axis of the N_4_ and O_4_ planes reduces from 40° to 29°.^16^

The tetra-amide shifted resonances analysed were the methyl and the phenyl ring protons. In each case, and as observed for every complex examined here in which the proton was >4.5 Å distant, the observed paramagnetic shift varied linearly with *T*^–2^ (ESI†). The degree of deviation from ideal Bleaney behaviour was assessed by plotting the shift *versus* the Bleaney coefficient, (Fig. 2 and ESI†). In each case, it was assumed that the plot went through the origin (yttrium case) and that Yb systems were the best behaved,1*c* and so these two points were used to define the line drawn, *i.e.* not a ‘best-fit’ plot. The plots for [Ln.L^4^(H_2_O)]^3+^ show a reasonable correlation, indeed better than for any other system examined here, although the Ho, Er and Tm cases showed significant deviations.

**Fig. 1:**
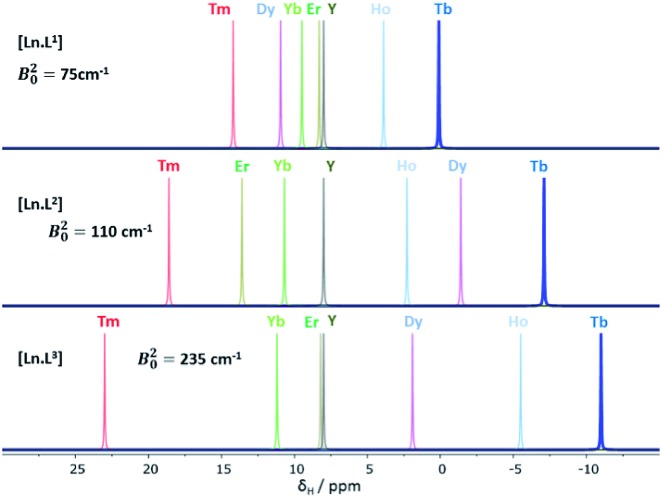
Schematic illustration of the chemical shift behaviour of the pyridyl H^3^ resonance in [Ln.L^1–3^]; (295 K, 9.4 T, [Ln.L^1^] in D_2_O, [Ln.L^2,3^] in CD_3_OD); Bleaney *C*_*J*_ values: Tb(–89), Dy(–100), Ho(–39), Er(+33), Tm(+55), Yb(+22) do not correlate well with this shift behaviour (ESI† for plots). Similar plots arise for the other two pyridyl proton resonances (ESI†); the Y complex serves as the diamagnetic reference.

**Fig. 2:**
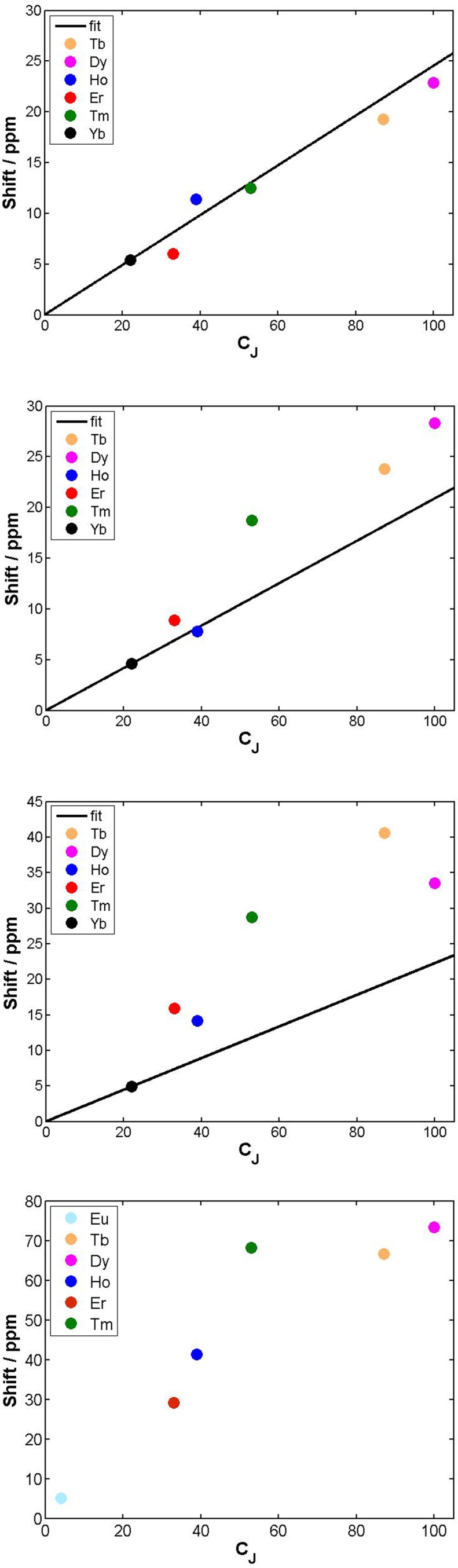
Top and upper centre: variation of the paramagnetic NMR shift of the methyl group {6.9 Å distant} (top) and *m*-phenyl proton resonance (7.0 Å) with the Bleaney constant, *C*_*J*_, in [Ln.L^4^(H_2_O)]^3+^; lower centre: shift variation for the methyl resonances in [Ln.L^5^]^–^ (4.7 Å) and (bottom) [Ln.DOTMA(H_2_O)]^–^ (4.9 Å) (295 K, D_2_O, 9.4 T); the Yb analogue does not form a *q* = 1 square antiprismatic isomer with DOTMA. Similar plots were obtained for the gDOTA series.

The second order crystal field splitting of [Ln.DOTMA(H_2_O)]^–^ is one of the largest considered here (–700 cm^–1^, major isomer *vs.* –470 cm^–1^ for the tetra-amide complex). The ^1^H NMR pseudocontact shifts of the methyl group for [Ln.DOTMA(H_2_O)]^–^ do not correlate very well with *C*_*J*_; the Tm(iii) complex in particular shows a large deviation.1*c* Similar behaviour is evident in the 8-coordinate phosphinate series, [Ln.L^5^]^–^, where even greater scatter was found for the methyl group shifts (Fig. 2; the Me proton is separated by 4 bonds from the Ln(iii) ion).

### Eight and nine-coordinate complexes in lower symmetry

The series of complexes, [Ln.L^6–9^] have lower time-averaged symmetry and allow a comparison of 9-coordinate (*q* = 1) carboxylate systems with 8-coordinate (*q* = 0) phosphinate analogues. In the complexes of L^6^, a CF_3_ group is located 6.1 Å from the metal centre,^19^ whereas in the other three cases a ^*t*^Bu group is 6.6 (±0.2) Å distant, each separated by 5 bonds. Paramagnetic shift data for the ^*t*^Bu resonance revealed dramatic differences between the 9 and the 8-coordinate complexes, (Table 3), that lack an axial donor.

**Table 3:** Chemical shift data of the ^*t*^Bu resonance of the major isomer in [Ln.L^7–9^] and the Bleaney constant, *C*_*J*_, (295 K, 9.4 T, D_2_O)

Ln^3+^	*δ*_H_/ppm [Ln.L^7^]	[Ln.L^8^]	[Ln.L^9^]^+^	*C*_*J*_
Tb	–11.6	–76.9	–7.2	–89
Dy	–20.5	–75.0	–17.8	–100
Ho	–7.4	–31.8	–7.0	–39
Er	7.0	38.2	3.4	+33
Tm	10.8	67.0	6.2	+55
Yb	6.3	16.3	9.1	+22
*B* 2 0 /cm^–1^	–550	–570	–350	

**Fig. 3:**
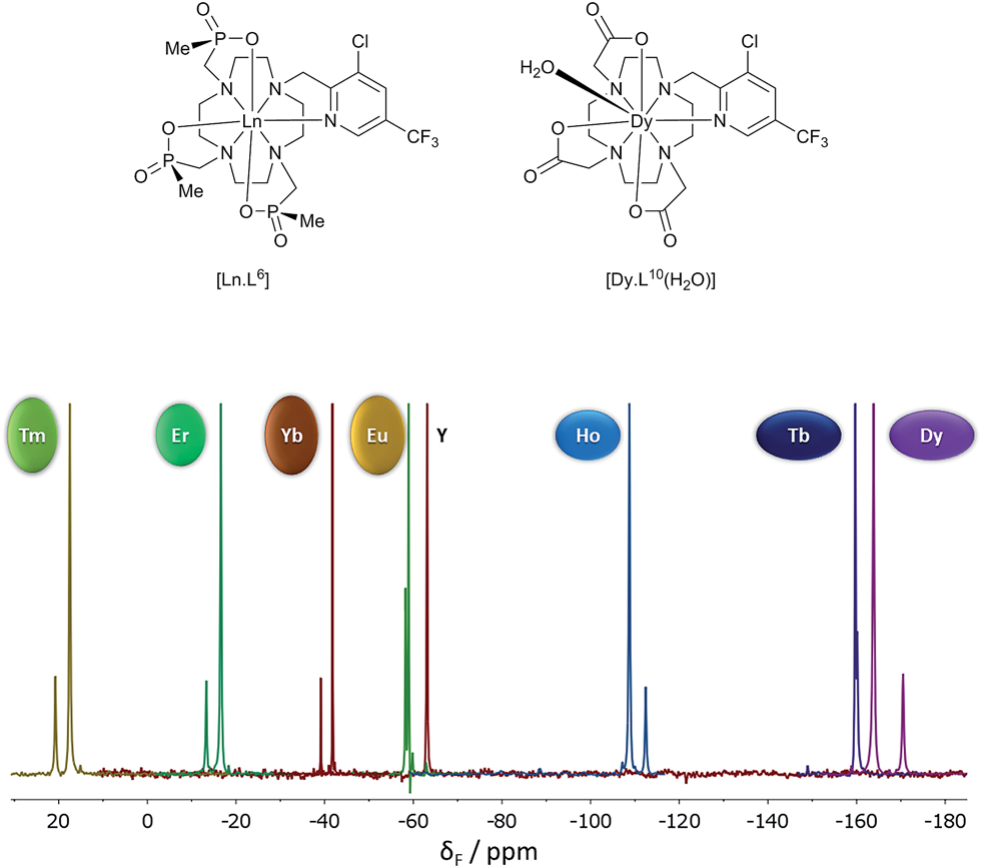
^19^F NMR spectra for [Ln.L^6^] (295 K, D_2_O), showing the two major chiral stereoisomers (RRR-Δ and RRR-Λ); the Y example serves as the diamagnetic reference.

The shift increased by over 50 ppm for the Tb, Dy and Tm complexes, which is independent of any change simply related to the variation of *B*20. The Tb, Ho and Er complexes of L^7^ and L^9^ show rather small shifts, about the same as for Yb in the latter case. The proton NMR dipolar shifts do not follow the Bleaney constant variation, within either series. Furthermore, when comparing the ^19^F shift of the CF_3_ resonances in [Dy.L^6^] (*δ*_F_ –162; dipolar shift –99 ppm) and the mono-aqua 9-coordinate analogue, [Dy.L^10^(H_2_O)],^27^ the difference was 47 ppm (*δ*_F_ –115; dipolar shift –52 ppm). The trend in the fluorine shift correlates fairly well with the *C*_*J*_ value, except for the Tm complex (Fig. 3).

It has been shown that the dipolar shift in the 9-coordinate cationic complexes of L^4^ with Eu and Yb, is primarily determined by the nature of the neutral axial donor group and its relative polarisability.^26^ Therefore, it was considered appropriate to compare complexes of ions with an oblate electron density distribution (*e.g.* Ce, Tb), examining their shift sensitivity to axial donor permutation. Accordingly, the variation of the ligand proton resonances in these complexes as a function of axial donor polarisability was examined in solution, under similar conditions to those studied originally (dry CD_3_CN, 295 K, 10 fold excess of added donor: (H_2_O, MeOH, DMAP, DMF, DMSO)). It was found that changing the donor in the Ce, Tb and Tm complexes gave rise to proportionate pseudocontact shift variations in each case, similar in relative size to those observed for Eu and Yb complexes, (ESI†). This finding lends support to the notion that the nature of the axial donor in *C*_*n*_ symmetric complexes is the major factor determining the dipolar ligand field interaction, in a manner that is proportional to the second order term, *B*20,^26^ and that this effect is largely independent of the spatial distribution of the f electron cloud (prolate or oblate).

## Summary and conclusions

Bleaney theory suggests that there are four key terms that determine the pseudocontact shift: the absolute temperature; the ligand field, defining the interaction between the f electron cloud and the ligand donors; the geometric coordinates of the nucleus with respect to the main axis of magnetisation; the degree of spin–orbit coupling and its relative size with respect to the ligand field. The latter term is related to the Bleaney constant (sign/magnitude), yet is confounded with any ligand field parameter.

The temperature dependence of the pseudocontact shift strictly followed a *T*^–2^ relationship for each case examined here. For resonances where a dominant pseudocontact shift occurs (at least 4 bonds separated; most resonances examined here are >6 Å), a strict *T*^–2^ variation of the paramagnetic shift was followed in every example measured (ESI†); resonances subject to a major contact shift contribution are predicted to show a *T*^–1^ variation.^1^,^2^

**Fig. 4:**
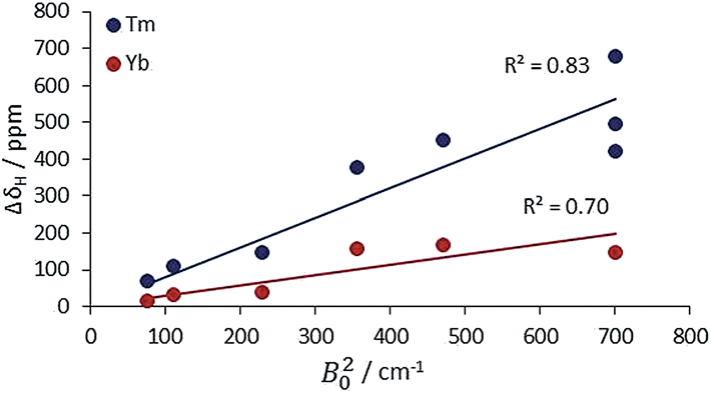
Variation of the total ^1^H NMR spectral width with *B*20 for Tm and Yb complexes with axial symmetry (ESI† for tabulated data).

The second main feature to consider is the proportionality of the pseudocontact shift with the ligand field parameter, *B*20, for axially symmetric isostructural series. The total ^1^H NMR spectral width for *C*_*n*_ symmetric complexes of Yb and Tm was plotted against *B*20 (Fig. 4). These two ions possess a similar f electron density distribution, and Yb complexes have been regarded, in the past, as adhering most faithfully to Bleaney theory, as they possess the largest pseudocontact/contact shift ratio.1*d* The linear correlations (*R*^2^ = 0.70 and 0.83 for Yb and Tm respectively) reveal the extent of this dependence. This behaviour is supported by the ligand-field/shift dependence in complexes of L^4^ (Ce, Eu, Tb, Dy, Tm, Yb), when the axial donor is permuted.

In the complexes of lower symmetry examined herein, higher order ligand field terms must be invoked, relating to multipolar contributions to the overall electrostatic interaction. Such terms are considered, for example, in assessing the non-linear optical behaviour of lanthanide coordination complexes.^13^ The higher order ligand field parameters, *Bkq*, where *k* = 4 and 6, can be over 1000 cm^–1^ in size, according to data reported from detailed analyses of emission spectra.^28^,^29^

The very large changes observed in the chemical shift of both the CF_3_ groups in the Dy, Er and Tm complexes of L^6^ and L^10^ and the ^*t*^Bu resonances in L^7^–L^9^, cannot be attributed simply to ligand field variation, as defined by *B*20 (Table 3), nor to any significant conformational shift of the position of these groups in these relative rigid structures. One explanation is to consider the extent to which the principal axis of magnetisation might have changed position from the complexes of the prolate (*e.g.* Yb, Tm, Eu) to the oblate ions (*e.g.* Tb, Ce, Dy, Ho). Thus, the angular terms defining the multipolar interaction can vary from one lanthanide to another, especially the rhombic terms (that are related to the higher order crystal field coefficients), as these complexes are not axially symmetric.

This hypothesis is supported by consideration of the shift behaviour of the 9-coordinate, *C*_3_ symmetric systems, [Ln.L^1–3^], in which the ligand field splitting is the smallest of the cases examined here. No obvious explanation can be put forward for the anomalous shift behaviour of the complexes of Dy, Er and Ho, examining the pyridyl resonances that reside four or five bonds (5.5 to 6.5 Å) away from the paramagnetic centre (Table 1 and ESI†). Moreover, there is no compelling evidence to suggest that there is a significant contact contribution in these cases. Indeed, the modified Reilley plots (ESI† pp. 24–28) suggest that any contact contribution is very small. Furthermore, Tb and Dy have similar intrinsic contact shift sensitivities^1^,^2^ yet behave completely differently (Fig. 1). One can hypothesise that either the position of the principal axis is varying in these systems or that the *C*_*J*_ values are not robust constants and may not be independent parameters, *i.e.* they are confounded.^7^ Of course, such a situation more obviously arises in cases where the ligand field is large, when *J* may not be a ‘good’ quantum number, so that *C*_*J*_ values are less likely to be robust, as in the low symmetry phosphinate complexes.

A related issue that emerges from this analysis, is that the generally accepted model for lanthanide paramagnetism (Landé, van Vleck) is based on the assumption that the ligand field splitting is small compared to the spin–orbit coupling, and that *J* is a good quantum number. This approximation led to the premise that the magnetic susceptibility of lanthanide complexes is independent of coordination environment. Evidently, it appears that this may not necessarily be the case for coordination complexes with a relatively large ligand field splitting. Further evidence in support of such a hypothesis, addressed by examining the field-dependent relaxation behaviour of these systems, will be reported in the near future.

Finally, this study provides some guidance in the design of chemical shift (‘PARASHIFT’) probes for use in magnetic resonance imaging and spectroscopy.^20^ A pre-requisite in the design of such probes for use *in vivo*, is to observe a fast-relaxing, reporter resonance that is shifted by over 10 000 Hz from the water (and fat) signals, as this allows fast pulse sequences to be used with large sweep widths. The ^*t*^Bu resonances of the low-symmetry, 8-coordinate pyridyl-triphosphinate series, *e.g.* [Ln.L^8^], examined here are much better suited for this application than analogous 9-coordinate carboxylate complexes. They possess relaxation rates of the order of 100 s^–1^ at 3 to 7 Tesla, and a very large proton chemical shift of +67 (Tm) or –75 (Dy) ppm, Moreover, their linewidths are not too great (ESI†), and *R*_1_/*R*_2_ ratios are in the range 0.5 to 0.85 at 4.7 T, allowing the use of fast imaging pulse sequences.

Further details of ‘PARASHIFT’ imaging *in vivo*, with systems related to [Ln.L^8^] will be reported shortly, using this approach.

## Supplementary Material

Supplementary information

Crystal structure data
