# Supplementary material for: Critical analysis of the limitations of Bleaney's theory of magnetic anisotropy in paramagnetic lanthanide coordination complexes
Source: Chem Sci. 2014 Dec 17;6(3):1655–62. doi: 10.1039/c4sc03429e (PMC5812375; doi:10.1039/c4sc03429e)
Supplement: Supplementary file 1 [file SC-006-C4SC03429E-s001.pdf]

Electronic Supplementary Material (ESI) for Chemical Science.
This journal is © The Royal Society of Chemistry 2014

Critical analysis of the limitations of Bleaney's theory of magnetic anisotropy in paramagnetic lanthanide coordination complexes †

Alexander M. Funk, Katie-Louise N. A. Finney, Peter Harvey, Alan M. Kenwright, Nicola J. Rogers, P. Kanthi Senanayake and David Parker*

ESI:

1. **General Procedures**
2. **Synthetic procedures**
3. **Tabulated shift data**
4. **Nuclear relaxation rate data at 5 fields**
5. **References**

1. General Procedures

The ligands and complexes of [Ln.L^1][1] and [Ln.L^5][2] were synthesised from known synthetic routes, which are found in the quoted references in the main text. The majority of complexes and their synthetic procedures have been published before: [Ln.L^1] [1] and [Ln.L^5]$^-$ [2] were synthesised from known routes, [Ln.L^2] prepared by Dr. James Walton[3], [Ln.L^3]$^{3+}$ prepared by Emily Neil[4], [Ln.L^4]$^{3+}$ was complexed from the ligand provided by Dr. Kanthi Senanayake[5], [Ln.L^{5Bz}]$^-$ was available from previous synthesis by Dr. Kanthi Senanyake[2] , [Ln.L^6][6], [Ln.L^7], [Ln.L^{7S}] and [Ln.L^9]$^+$ were prepared by Dr. Peter Harvey[7], [Tm.DOTMA]$^-$ was provided by Dr. Mauro Botta[8] and [Ln.gDOTA]$^{5-}$ samples were synthesised from known routes with the help of Dr. Neil Sim[9]. The complex series [Ln.L^8] was prepared by Dr. Kanthi Senanayake at Durham University. An overview of the structures is given in Scheme 1.

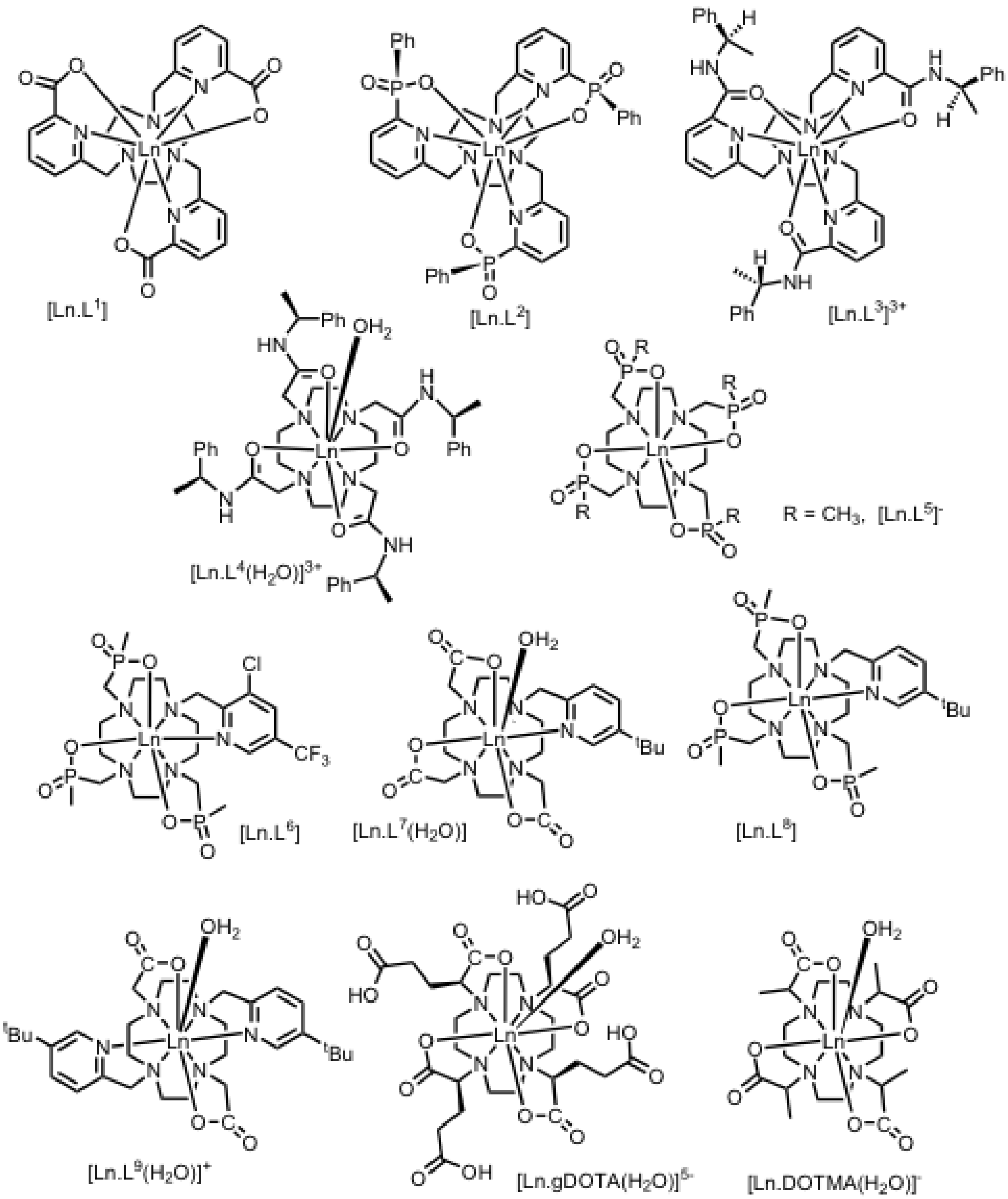

Scheme 1 : *Structures of all complexes analysed in this discussion.*

1.1 Structural analysis

The X-ray structures for [Ln.L^1][1], [Ln.L^2][3], [Ln.L^3]$^{3+}$[4] and [Ln.L^4]$^{3+}$[5] were taken from the quoted literature structures. The [Tm.DOTMA(H_2O)]$^-$[8] structure was provided by Dr. Mark Woods of Oregon Health & Science University.

It was found the quality of crystals of [Tm.gDOTA(H_2O)]$^{5-}$ complex has deteriorated rapidly on cooling so the crystal was mounted on a Bruker D8Venture diffractometer (Photon-100 CMOS detector, ImuS microsource with focusing mirrors, Oxford Cryostream open-flow nitrogen cryostat) at 250.0 K and was

cooled slowly to 200.0 K. Single crystal X-ray data were collected at that temperature (ω-scan, 0.5°/frame).

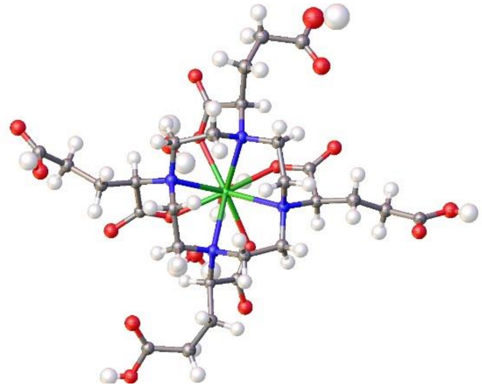

SI-Figure 1 : *Illustration of the X-ray crystallographic structure of the minor isomer (SAP, q=1) of $[Tm.gDOTA]^{5-}$, isostructural with the Eu, Gd, Tb structures*

The structure was solved by Patterson method and refined by full matrix least squared on F^2 for all data using SHELXTL (G.M. Sheldrick, Acta Cryst. (2008). A64, 112-122) and OLEX2 (O. V. Dolomanov, L. J. Bourhis, R. J. Gildea, J. A. K. Howard and H. Puschmann, J. Appl. Cryst. (2009). 42, 339-341) software. All non-hydrogen atoms were refined with anisotropic displacement parameters. Hydrogen atoms were placed into calculated positions and refined in "riding"-mode. Crystallographic data for the structure of $[Tm.gDOTA(H_2O)]^{5-}$ complex have been deposited with the Cambridge Crystallographic Data Centre as supplementary publication CCDC-1032378. When the distances of protons were measured their distances were corrected using the OLEX2 software.

1.2 General NMR procedures

^{1}H, ^{19}F and ^{31}P NMR spectra were obtained at 295 K (unless stated otherwise) on Varian spectrometers operating at 4.7, 9.4, 11.7, 14.1, 16.5 Tesla, specifically on a Mercury 200 spectrometer (^{1}H at 200.057 MHz, ^{19}F at 188.242 MHz, ^{31}P at 80.985 MHz), a Mercury 400 spectrometer (^{1}H at 399.97 MHz, ^{19}F at 376.331 MHz, ^{31}P at 161.910 MHz), a Varian Inova-500 spectrometer (^{1}H at 499.78 MHz, ^{19}F at 470.322 MHz, ^{31}P at 202.340 MHz), a Varian VNMRS-600 spectrometer (^{1}H at 599.944 MHz, ^{19}F at 564.511 MHz, ^{31}P at 242.862 MHz) and a Varian VNMRS-700 spectrometer (^{1}H at 700.000 MHz, ^{19}F at 658.658 MHz, ^{31}P at 283.365 MHz). Commercially available deuterated solvents were used.

The operating temperature of the spectrometers was measured with the aid of an internal calibration sample of neat ethylene glycol for high temperature studies. A calibration sample of neat methanol was used for low temperatures studies. The operating temperature of each spectrometer was measured before each set of measurements of relaxation data.

The ^{19}F and ^{31}P relaxation data were measured without proton decoupling. The ^{31}P chemical shifts are reported relative to 85 % phosphoric acid. The ^{19}F chemical shifts are reported relative to fluorotrichloromethane.

The recorded free induction decays were processed using backward linear prediction, optimal exponential weighting, zero-filling, Fourier transform, phasing and baseline correction (by Whittaker smoothing), if necessary.

1.3 Relaxation data analysis

The nuclear relaxation times of the nuclei of interest were measured at the five different fields mentioned above. The T_1 values were measured using the inversion-recovery technique. At first a crude T_1 value was obtained, which was then used as the initial guess in multiple repeat experiments. The incremented delay time was set to show full inversion and full recovery to equilibrium of the signal, which is roughly achieved at five times the T_1 value.

The line width and T_2 data were obtained by Lorentzian line fitting. Due to the broad nature of the resonances, the field inhomogeneity is negligible and it can be assumed that in the absence of exchange broadening the T_2* measured from the line width corresponds to the actual T_2 value.[10]

The lanthanide(III) complexes of [Ln.L^2] and [Ln.L^3]$^{3+}$ were analysed in CD_3OD, the remaining complexes series were analysed in D_2O solutions, unless stated otherwise. The concentration of a sample was kept constant throughout a series of measurements, which was in the range of 0.1 to 1 mM. For each complex studied the ^1H, ^{19}F and ^{31}P relaxation data considered in this work can be found in the references[4,7,11] or is given herein.

The measured nuclear relaxation data was fitted by using a modified Matlab algorithm originally written by Dr. Ilya Kuprov of Southampton University. The algorithm uses the Solomon-Morgan-Bloembergen equation (1) to fit the measured relaxation data using the Matlab internal Levenberg-Marquardt minimisation of the non-linear squares error function. The results were analysed iteratively. It was assumed that the longitudinal and transverse electronic relaxation times were of a similar magnitude.

$$R_1 = \frac{2}{15}\left(\frac{\mu_0}{4\pi}\right)^2 \frac{\gamma_N^2 g_{Ln}^2\, \mu_B^2\, J\,(J+1)}{r^6}\left[3\frac{T_{1e}}{1+\omega_N^2\, T_{1e}^2} + 7\frac{T_{2e}}{1+\omega_e^2\, T_{2e}^2}\right] + \frac{2}{5}\left(\frac{\mu_0}{4\pi}\right)^2 \frac{\omega_N^2 \mu_{eff}^4}{(3k_B T)^2\, r^6}\left[3\frac{\tau_r}{1+\omega_N^2\, \tau_r^2}\right] \quad (1)$$

Some parameters were used globally for every lanthanide(III) complex in the series and others were used for each complex individually. The rotational correlation time, τ_r, was considered not to vary across a given series of complexes. An estimate for τ_r was determined by the Stokes-Einstein Law. An estimate of the hydrodynamic radius, *r*, was made by inspecting the X-ray data; in each case a reasonable agreement was found (± 0.2 Å). DFT calculations were performed by Dr. Ilya Kurpov for further analysis of internuclear distances and rotational correlation times.[12] The consistency and validity of this method was discussed in detail previously.[11]

1.3 Error Analysis

Each relaxation measurement was repeated at least three times and the mean value recorded. The number of transients used in the measurements was determined by the signal-to-noise ratio and also by the linewidth of the resonance of interest. In each case, the signal was fully recovered during the inversion-recovery sequence.

A statistical error analysis was undertaken to determine the fitting errors. The experimental errors of the measured relaxation rates were combined and used to perturb the relaxation rates for each complex at each field. These perturbed rates together with the unperturbed relaxation rates were used in a statistical error analysis to obtain the error values for the individual parameters (μ_{eff}, *r*, τ_r and T_{1e}) calculated in the fitting process.[13] An example of the obtained estimates is given in SI-Table 1.

SI-Table 1: *Variation of the fitting results when perturbing the experimental nuclear relaxation data within their respective errors for the global fitting of the Heq' resonance of* $[Ln.L^4]^{3+}$ *(295 K,* D_2O*).*

		μ_{eff} / BM				τ_R / ps			T_{1e} / ps			
Eu	Tb	Dy	Ho	Tm	Yb		Eu	Tb	Dy	Ho	Tm	Yb
2.990	8.909	10.325	9.951	7.578	4.344	335.3	0.141	0.564	0.382	0.281	0.262	0.200
2.836	8.898	10.320	9.946	7.568	4.311	334.9	0.101	0.567	0.380	0.278	0.256	0.183
2.979	8.917	10.330	9.956	7.587	4.379	335.6	0.176	0.568	0.386	0.285	0.267	0.216
2.865	8.903	10.323	9.949	7.574	4.328	335.3	0.122	0.568	0.381	0.280	0.259	0.192
2.937	8.911	10.327	9.953	7.592	4.361	335.4	0.159	0.569	0.384	0.283	0.265	0.208

1.4 Pseudocontact shift analysis

The ^1H NMR spectra for the complexes $[Ln.L^{1-5}]$ were fully characterised and analysed. The remaining complex series were only partially analysed, as the attention was given to specific reporter groups (e.g.

^{t}Bu, CF_3), due to varying amounts of available sample material and more difficult analysis in the low symmetry complexes.

The accurate measurement of the chemical shift of a given resonance was performed on the 9.4 Tesla Mercury 400 spectrometer, unless stated otherwise.

The observed shifts were tested for changes due to the bulk magnetic susceptibility shift of the lanthanide(III) complexes. Using a coaxial insert tube containing a tert-butanol reference (1%), it was found that over the concentration range used here (0.1 – 1 mM), the BMS alteration to the observable shift was less than 0.2 ppm. Considering that the observed shift range can vary from +500 ppm to -500 ppm in these paramagnetic complexes, this small effect can be largely neglected at the concentrations used here.

1.5 Variable temperature NMR studies

The variable temperatures ^{1}H and ^{19}F NMR resonances (D_2O except [Ln.L^{2}] and [Ln.L^{3}]$^{3+}$ (CD_3OD)) of most complexes were measured as a function of the temperature on the Varian VNMRS-600 spectrometer, with a few exceptions run on the Varian VNMRS-700 spectrometer, as stated in the discussion. However, the VT experiments on [Tm.L^{4}] were performed on the Varian Inova-500 spectrometer, due to restrictions for lowering the temperature on the other high field spectrometers. In each measurement, the temperature was measured with the internal calibration standard (ethylene glycol or methanol) to ensure accurate temperature measurements.[14] The error for the shift per unit Kelvin was calculated using linear regression.

2. Synthetic procedures of [Ln.L^{6}], [Ln.L^{8}] and [Ln.L^{10}]

Tri-*tert*-butyl 10-((3-chloro-5-(trifluoromethyl)pyridin-2-yl)methyl)-1,4,7,10-tetraazacyclododecane-1,4,7-tricarboxylate

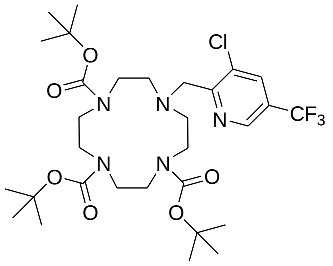

A stirred mixture of tri-BOC-cyclen (2.26 g, 4.79 mmol), 3-chloro-2-(chloromethyl)-5-(trifluoromethyl)pyridine (1.00 g, 4.35 mmol), and K_2CO_3 (0.90 g, 6.53 mmol) in anhydrous MeCN (25 mL) was boiled under reflux for 18 h under argon. The reaction mixture was cooled and filtered before

solvent was removed under reduced pressure. The resulting yellow oil was purified by silica gel column chromatography, eluting with a gradient starting from 100 % DCM to 5 % MeOH/DCM to yield a pale yellow oil (1.84 g, 64 %). R_f (5 % MeOH/DCM) = 0.61. ^{1}H NMR (400 MHz, $CDCl_3$): δ = 8.75 (d, J = 1 Hz, 1H, H^6), 7.90 (d, J = 1 Hz, 1H, H^4), 4.13 (s, 2H, $NC\underline{H}_2py$), 3.59 (br s, 4H, cyclen-$C\underline{H}_2$), 3.40 (br s, 8H, cyclen-$C\underline{H}_2$), 2.84 (br s, 4H, cyclen-$C\underline{H}_2$), 1.47 (s, 9H, $C(C\underline{H}_3)_3$), 1.43 (s, 18H, $C(C\underline{H}_3)_3$); ^{13}C NMR (101 MHz, $CDCl_3$): δ = 159.1 (C^2), 155.7 ($\underline{C}O_2{}^tBu$), 155.4 ($\underline{C}O_2{}^tBu$), 143.6 (q, $^3J_{CF}$ = 4 Hz, C^6), 134.1 (q, $^3J_{CF}$ = 4 Hz, C^4), 132.0 (C^3), 126.2 (q, $^2J_{CF}$ = 34 Hz, C^5), 122.6 (q, $^1J_{CF}$ = 273 Hz, CF_3), 79.5 ($\underline{C}(CH_3)_3$), 79.2 ($\underline{C}(CH_3)_3$), 54.8 ($N\underline{C}H_2py$), 53.7 (cyclen-$\underline{C}H_2$), 50.1 (cyclen-$\underline{C}H_2$), 48.0 (cyclen-$\underline{C}H_2$), 47.7 (cyclen-$\underline{C}H_2$), 28.7 ($C(\underline{C}H_3)_3$), 28.5 ($C(\underline{C}H_3)_3$); ^{19}F NMR (376 MHz, $CDCl_3$): δ = -62.3; ESI/MS$^+$ m/z 666.5 $[M+H]^+$; HRMS Calcd for $C_{30}H_{48}ClF_3N_5O_6$ 666.3238. Found 666.3240.

1-((3-Chloro-5-(trifluoromethyl)pyridin-2-yl)methyl)-1,4,7,10-tetraazacyclododecane

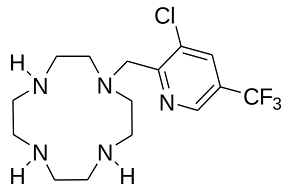

Tri-*tert*-butyl-10-((3-chloro-5-(trifluoromethyl)pyridin-2-yl)methyl)-1,4,7,10-tetraazacyclododecane-1,4,7-tricarboxylate (150 mg, 0.225 mmol) was dissolved in DCM (2 mL) with stirring. To this was added TFA (1 mL) and the mixture was stirred at rt for 18 h. After this time, the solvent was removed under reduced pressure before being repeatedly washed with DCM. The resulting oil was dissolved in $KOH_{(aq)}$ (1 M, 10 mL) and extracted with DCM (3 x 5 mL). The solvent was removed under reduced pressure to yield an orange oil (78 mg, 95 %). ^{1}H NMR (400 MHz, $CDCl_3$): δ = 8.68 (s, 1H, H^6), 7.87 (s, 1H, H^4), 3.92 (s, 2H, $NC\underline{H}_2py$), 2.76 (br m, 4H, cyclen-$C\underline{H}_2$), 2.67 (br s, 8H, cyclen-$C\underline{H}_2$), 2.53 (br s, 4H, cyclen-$C\underline{H}_2$); ^{13}C NMR (101 MHz, $CDCl_3$): δ = 159.9 (C^2), 143.6 (q, $^3J_{CF}$ = 4 Hz, C^6), 134.5 (q, $^3J_{CF}$ = 4 Hz, C^4), 132.2 (C^3), 126.6 (q, $^2J_{CF}$ = 34 Hz, C^5), 122.7 (q, $^1J_{CF}$ = 273 Hz, CF_3), 58.2 ($N\underline{C}H_2py$), 51.7 (cyclen-$\underline{C}H_2$), 46.9 (cyclen-$\underline{C}H_2$), 46.0 (cyclen-$\underline{C}H_2$), 44.8 (cyclen-$\underline{C}H_2$); ^{19}F NMR (376 MHz, $CDCl_3$): δ = -62.7; ESI/MS$^+$ m/z 366.3 $[M+H]^+$; HRMS Calcd for $C_{15}H_{24}ClF_3N_5$ 366.1684. Found 366.1672.

Triethyl(10-((3-chloro-5-(trifluoromethyl)pyridin-2-yl)methyl)-1,4,7,10-tetraazacyclododecane-1,4,7-triyl)tris(methylene)tris(methylphosphinate)

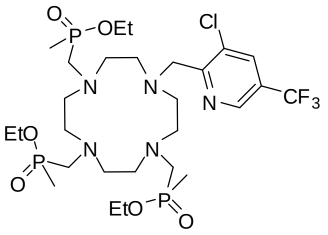

1-((3-Chloro-5-(trifluoromethyl)pyridin-2-yl)methyl)-1,4,7,10-tetraazacyclododecane (100 mg, 0.273 mmol) was heated to 80°C in anhydrous THF under argon. Paraformaldehyde (110 mg, 3.68 mmol) followed by diethoxymethylphosphine (500 mg, 3.68 mmol) were added. The solution was heated at reflux over molecular sieves for 15 h. After this time, the excess paraformaldehyde was removed by filtration and the solvent removed under reduced pressure. The resulting orange oil was purified by mixed alumina/silica (4:1) gel column chromatography, eluting with a gradient starting from 100 % DCM to 8 % MeOH/DCM, to yield a yellow oil (130 mg, 71 %), which was present as a mixture of diastereoisomers. ^1H NMR (400 MHz, $CDCl_3$): δ = 8.73 (s, 1H, H^6), 7.90 (s, 1H, H^4), 4.74 (s, 2H, $NC\underline{H}_2py$), 4.06 (m, 9H, $POC\underline{H}_2CH_3$), 3.03 (br m, 8H, $NC\underline{H}_2P$/cyclen-$C\underline{H}_2$), 2.79 (br m, 14H, $NC\underline{H}_2P$/cyclen-$C\underline{H}_2$), 1.53 (m, 9H, $PC\underline{H}_3$), 1.31 (m, 9H, $POCH_2C\underline{H}_3$); ^{13}C NMR (101 MHz, $CDCl_3$): δ = 158.8 (C^3), 142.5 (q, J = 4 Hz, C^6), 133.2 (q, $^3J_{CF}$ = 4 Hz, C^4), 131.0 (C^3), 129.8 (q, $^2J_{CF}$ = 34 Hz, C^5), 121.6 (q, $^1J_{CF}$ = 273 Hz, CF_3), 59.3 (d, $^2J_{CP}$ = 7 Hz, $PO\underline{C}H_2CH_3$), 56.2 ($N\underline{C}H_2py$), 53.9 (m, $N\underline{C}H_2P$/cyclen-$\underline{C}H_2$), 53.2 (m, $N\underline{C}H_2P$/cyclen-$\underline{C}H_2$), 52.8 (m, $N\underline{C}H_2P$/cyclen-$\underline{C}H_2$), 51.5 (m, $N\underline{C}H_2P$/cyclen-$\underline{C}H_2$), 15.7 (d, $^3J_{CP}$ = 6 Hz, $PCH_2\underline{C}H_3$), 12.7 (d, $^1J_{CP}$ = 89 Hz, $P\underline{C}H_3$), 12.5 (d, J = 92 Hz, $P\underline{C}H_3$); ^{19}F NMR (376 MHz, $CDCl_3$): δ = -63.2; ^{31}P NMR (162 MHz, $CDCl_3$): δ = 54.3, 53.4; ESI/MS$^+$ m/z 726.4 $[M+H]^+$, 748.4 $[M+Na]^+$; HRMS Calcd for $C_{27}H_{51}N_5O_6F_3ClP_3$ 726.2693. Found 726.2698.

(10-((3-Chloro-5-(trifluoromethyl)pyridin-2-yl)methyl)-1,4,7,10-tetraazacyclododecane-1,4,7-triyl)tris(methylene)tris(methylphosphinic acid) , L^6

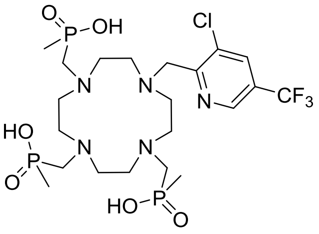

Triethyl-(10-((3-chloro-5-(trifluoromethyl)pyridin-2-yl)methyl)-1,4,7,10-tetraazacyclododecane-1,4,7-triyl)tris(methylene)tris(methylphosphinate) (125 mg, 0.17 mmol) was dissolved in HCl (6 M, 10 mL) and stirred for 18 h at 90°C. The solvent was removed under reduced pressure and the residue washed repeatedly with DCM (3 x 5 mL) before the resulting oil was dissolved in EtOH. The white precipitate was removed by filtration and the solvent removed under reduced pressure to yield the hydrochloride salt as a yellow oil (107 mg, 98 %). ^1H NMR (400 MHz, D_2O) δ = 8.72 (s, 1H, H^6), 8.11 (s, 1H, H^4), 3.59 (m, 2H, $NC\underline{H}_2py$), 3.43 (br s, 8H, $NC\underline{H}_2P$/cyclen-$C\underline{H}_2$), 3.07 (br m, 14H, cyclen-$C\underline{H}_2$), 1.26 (br m, 9H, $PC\underline{H}_3$); ^{19}F NMR (376 MHz, $CDCl_3$) δ = -63.0; ^{31}P NMR (162 MHz, $CDCl_3$) δ = 53.4; ESI/MS$^-$ m/z 640.3 $[M-H]^-$.

[Dy.L[6]]

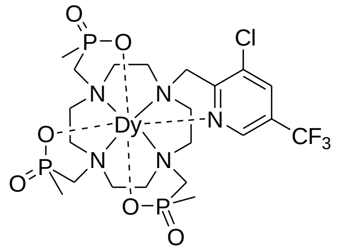

$Dy(III)Cl_3.6H_2O$ (8.8 mg, 0.0234 mmol) was added to a solution of (10-((3-chloro-5-(trifluoromethyl)pyridin-2-yl)methyl)-1,4,7,10-tetraazacyclododecane-1,4,7-triyl)tris(methylene)tris(methylphosphinic acid) (10.7 mg, 0.0167 mmol) dissolved in H_2O (5 mL). The pH was adjusted to 5.5 before stirring the solution for 18 h at 40°C. After this time, the solution was allowed to cool to rt before the pH was raised to 10, causing a white solid to precipitate out of solution. This precipitate was removed by centrifugation and the pH of the resulting solution neutralised. The solvent was removed under reduced pressure to yield a yellow solid, which was purified by preparative HPLC to yield an off-white solid (13 mg, 97 %). ^{19}F NMR (376 MHz, D_2O, pH 6.5): δ = -163.5 (major), -170.2 (minor); ESI/MS$^-$ m/z 837.4 [M+Cl]$^-$; HRMS Calcd for $C_{21}H_{35}{}^{35}Cl_2{}^{160}DyF_3N_5O_6P_3$ 833.0381. Found 833.0401.

[Er.L[6]] ^{19}F NMR (376 MHz, D_2O, pH 6.5): δ = -16.9 (major), -13.5 (minor); ESI/MS$^+$ m/z 805.0 [M+H]$^+$; HRMS Calcd for $C_{21}H_{35}{}^{35}Cl^{166}ErF_3N_5O_6P_3Na$ 827.0642. Found 827.0616.

[Tb.L[6]] ^{19}F NMR (376 MHz, D_2O, pH 6.5): δ = -158.4 (major), -160.8 (minor); ESI/MS$^+$ m/z 820.3 [M+Na]$^+$; HRMS Calcd for $C_{21}H_{35}{}^{35}Cl^{159}TbF_3N_5O_6P_3Na$ 820.0592. Found 820.0617. τ_{H2O} = 3.33ms; τ_{D2O} = 3.61 ms; q = 0.

[Tm.L[6]] ^{19}F NMR (376 MHz, D_2O, pH 6.5): δ = 17.1 (major), 20.3 (minor); ESI/MS$^+$ m/z 830.2 [M+Na]$^+$; HRMS Calcd for $C_{21}H_{35}{}^{35}Cl^{169}TmF_3N_5O_6P_3Na$ 830.0681. Found 830.0701.

[Ho.L[6]] ^{19}F NMR (376 MHz, D_2O, pH 6.5): δ = -107.8 (major), -112.4 (minor); ESI/MS$^+$ m/z 826.2 [M+Na]$^+$; HRMS Calcd for $C_{21}H_{35}{}^{35}Cl^{165}HoF_3N_5O_6P_3Na$ 826.0642. Found 826.0659.

[Gd.L[6]] ^{19}F NMR (376 MHz, D_2O, pH 6.5): δ = -63.8 (broad); ESI/MS$^+$ m/z 819.2 [M+Na]$^+$; HRMS Calcd for $C_{21}H_{35}{}^{35}Cl^{154}GdF_3N_5O_6P_3Na$ 815.0547. Found 815.0559.

[Eu.L[6]] ^{19}F NMR (376 MHz, D_2O, pH 6.5): δ = -58.6 (major), -58.2 (minor); ESI/MS$^+$ m/z 814.2 [M+Na]$^+$; HRMS Calcd for $C_{21}H_{35}{}^{35}Cl^{151}EuF_3N_5O_6P_3Na$ 812.0537. Found 812.0553.

[Y.L[6]] ^{19}F NMR (376 MHz, D_2O, pH 6.5): δ = -64.0 (major), -63.0 (minor); ESI/MS$^+$ m/z 750.2 [M+Na]$^+$; HRMS Calcd for $C_{21}H_{35}{}^{35}ClYF_3N_5O_6P_3Na$ 750.0397. Found 750.0415.

[Yb.L^6] ^{19}F NMR (376 MHz, D_2O, pH 6.5): δ = -41.5 (major), -39.1 (minor); ESI/MS$^-$ m/z 845.1 [M+Cl]$^-$; HRMS Calcd for $C_{21}H_{35}{}^{35}Cl_2{}^{170}YbF_3N_5O_6P_3$ 843.0482. Found 843.0463.

***tert*-Butyl 2,2',2''-(10-((3-chloro-5-(trifluoromethyl)pyridin-2-yl)methyl)-1,4,7,10-tetraazacyclododecane-1,4,7-triyl)triacetate**

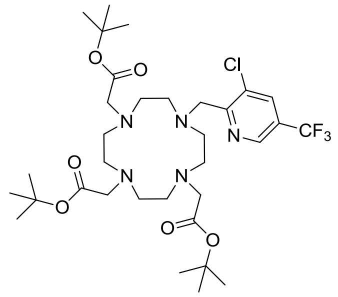

A stirred mixture of *tert*-butyl 2,2',2''-(1,4,7,10-tetraazacyclododecane-1,4,7-triyl)triacetate (DO3A) (0.90 g, 1.74 mmol), 3-chloro-2-(chloromethyl)-5-(trifluoromethyl)pyridine (0.48 mg, 2.09 mmol), K_2CO_3 (0.34 g, 2.44 mmol), and KI (ca. 5 mg) in anhydrous MeCN (25 mL) was boiled under reflux for 18 h under argon. The reaction mixture was cooled and filtered before the solvent was removed under reduced pressure. The resulting yellow oil was purified by silica gel column chromatography, eluting with a gradient starting from 100 % DCM to 6 % MeOH/DCM to yield a yellow oil (0.84 g, 68 %). R_f (10 % MeOH/DCM) = 0.40. ^{1}H NMR (400 MHz, $CDCl_3$): δ = 8.38 (d, *J* = 2 Hz, 1H, H^6), 7.87 (d, *J* = 2 Hz, 1H, H^4), 3.81 (s, 2H, NC$\underline{H}_2$py), 3.60 – 2.64 (br m, 12H, NC$\underline{H}_2CO_2{}^tBu$/cyclen-C$\underline{H}_2$), 2.13 (br m, 10H, cyclen-C$\underline{H}_2$), 1.39 (s, 18H, ^{t}Bu), 1.18 (s, 9H, ^{t}Bu); ^{13}C NMR (101 MHz, $CDCl_3$): δ = 171.8 ($\underline{C}O_2{}^tBu$), 159.6 (C^2), 141.9 (q, $^3J_{CF}$ = 4 Hz, C^6), 132.9 (q, $^3J_{CF}$ = 7 Hz, C^4), 130.2 (C^3), 125.0 (q, $^2J_{CF}$ = 34 Hz, C^5), 121.5 (q, $^1J_{CF}$ = 273 Hz, CF_3), 81.1 ($\underline{C}(CH_3)_3$), 55.8 (N$\underline{C}H_2CO_2{}^tBu$), 54.2 (cyclen-$\underline{C}H_2$), 52.5 (cyclen-$\underline{C}H_2$), 26.8 ($C(\underline{C}H_3)_3$), 26.7 ($C(\underline{C}H_3)_3$); ^{19}F NMR (376 MHz, $CDCl_3$): δ = -63.4; ESI/MS$^+$ m/z 730.5 [M+Na]$^+$; HRMS Calcd for $C_{33}H_{53}ClF_3N_5NaO_6$ 730.3534. Found 730.3530.

2,2',2''-(10-((3-Chloro-5-(trifluoromethyl)pyridin-2-yl)methyl)-1,4,7,10-tetraazacyclododecane-1,4,7-triyl)triacetic acid , L^{10}

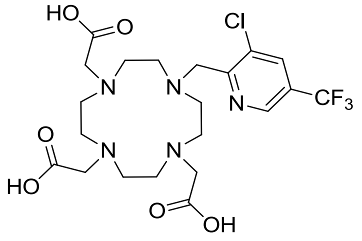

tert-Butyl 2,2',2''-(10-((3-chloro-5-(trifluoromethyl)pyridin-2-yl)methyl)-1,4,7,10-tetraazacyclododecane-1,4,7-triyl)triacetate (100 mg, 0.141 mmol) was dissolved in HCl (6 M, 10 mL) and stirred for 18 h at 90°C. The solvent was removed under reduced pressure and the residue washed repeatedly with DCM (3 x 5 mL) and the resulting oil was dissolved in EtOH. The white precipitate was removed by filtration

and the solvent removed under reduced pressure to yield the hydrochloride salt as a yellow oil (75.5 mg, 99 %). ^{1}H NMR (400 MHz, D_2O): δ = 8.53 (s, 1H, H^6), 8.19 (s, 1H, H^4), 4.07 (br s, 2H, NC$\underline{H}_2$py), 3.75 – 3.25 (br m, 12H, NC$\underline{H}_2$$CO_2$H/cyclen-C$\underline{H}_2$), 3.10 (br m, 10H, cyclen-C$\underline{H}_2$); ^{19}F NMR (376 MHz, D_2O): δ = -63.1; ESI/MS$^+$ m/z 540.3 [M+H]$^+$, ESI/MS$^-$ m/z 538.3 [M-H]$^-$.

[Dy.L^{10}](H_2O)] Dy(III)Cl_3.6H_2O (33.5 mg, 8.9 mmol) was added to a solution of 2,2',2''-(10-((3-chloro-5-(trifluoromethyl)pyridin-2-yl)methyl)-1,4,7,10-tetraazacyclododecane-1,4,7-triyl)triacetic acid (40 mg, 0.074 mmol) dissolved in H_2O (5 mL). The pH was adjusted to 5.5 before stirring the solution for 18 h at 40°C. After this time, the solution was allowed to cool to rt before the pH was raised to 10, causing a white solid to precipitate out of solution. This precipitate was removed by centrifugation and the pH of the resulting solution neutralised. The solvent was removed under reduced pressure to give a yellow solid, which was purified by preparative HPLC to yield an off-white solid (49 mg, 95 %). ^{19}F NMR (376 MHz, D_2O, pH 6.5): δ = -115.9 (major), -126.5 (minor); ESI/MS$^+$ m/z 722.8 [M+Na]$^+$; HRMS Calcd for $C_{21}H_{26}{}^{35}Cl^{160}DyF_3N_5O_6Na$ 719.0668. Found 719.0652.

[Tm.L^{10}(H_2O)] ^{19}F NMR (376 MHz, D_2O, pH 6.5): δ = -40.9 (major), -5.6 (minor); ESI/MS$^+$ m/z 728.7 [M+Na]$^+$; HRMS Calcd for $C_{21}H_{26}{}^{35}Cl^{169}TmF_3N_5O_6Na$ 706.0939. Found 706.0915.

5-*tert*-Butyl-2-methylpyridine (1).

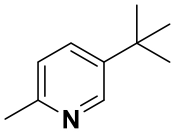

1

Copper(I) cyanide (10 mg) was added to anhydrous diethyl ether (50 mL) and the resulting suspension cooled to -78°C. To this was added *tert*-butylmagnesium chloride (14.5 mL, 2.0 M solution in diethyl ether, 29.1 mmol) and the mixture was maintained at -78°C, stirred for 20 min under argon and 5-bromo-2-methylpyridine (2.5 g, 14.5 mmol) in THF (10mL) was added. The reaction was stirred for 3 h at -78°C before being allowed to warm to rt and stirred for a further 18 h. Upon completion of the reaction, sat.$NH_4OH_{(aq)}$ was added drop wise to quench any excess Grignard reagent remaining in solution. This mixture was extracted with diethyl ether (3 x 50 mL), the organic layers were combined, dried over $MgSO_4$ and solvent removed under reduced pressure. The resulting yellow liquid was carried on to the next step without further purification, (1.42 g, 66 %). R_f (50 % ethyl acetate/hexane) = 0.35; ^{1}H NMR (400 MHz, $CDCl_3$): δ 8.31 (d, *J* = 2 Hz, 1H, H^6), 7.63 (dd, *J* = 8, 2 Hz, 1H, H^4), 7.10 (d, *J* = 8 Hz, 1H, H^3), 2.39 (s, 3H, Me), 1.24 (s, 9H, ^{t}Bu); ^{13}C NMR (101 MHz, $CDCl_3$): δ 157.6 (C^2), 148.0 (C^6), 146.5 (C^5), 137.4 (C^4), 126.1 (C^3), 36.2 ($\underline{C}(CH_3)_3$), 33.2 (C($\underline{C}H_3$)), 23.6 ($\underline{C}H_3$); ESI-LRMS (+) *m/z* 150.2 [M+H]$^+$; ESI-HRMS (+) calcd for $C_{10}H_{16}N$ 150.1283, found 150.1279. The NMR data was in good agreement with the data reported previously.[15]

5-*tert*-Butyl-2-methylpyridine 1-oxide (2).

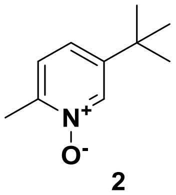

2

*m*CPBA (2.17 g, 12.6 mmol) was added to a solution of 5-*tert*-butyl-2-methylpyridine (1.25 g, 8.39 mmol) in chloroform (30 mL). The resulting solution was stirred at rt for 18 h under argon, before being quenched with sat. $Na_2SO_{4(aq)}$ (10 mL) and stirred for 10 min. The organic layer was extracted and washed with sat. $NaHCO_{3(aq)}$ (30 mL). The aqueous layer was extracted with ethyl acetate (3 x 25 mL); the organic layers were combined, dried over Na_2SO_4, and the solvent removed under reduced pressure. The resulting yellow liquid was purified by silica gel column chromatography, eluting with a gradient starting from 100 % DCM to 5 % MeOH / DCM to yield a pale yellow liquid (0.51 g, 37 %). R_f (10 % MeOH / DCM) = 0.39; ^{1}H NMR (400 MHz, $CDCl_3$): δ 8.32 (d, *J* = 2 Hz, 1H, H^6), 7.21 (dd, *J* = 8, 2 Hz, 1H, H^4), 7.18 (d, *J* = 8 Hz, 1H, H^3), 2.50 (s, 3H, Me), 1.31 (s, 9H, ^{t}Bu); ^{13}C NMR (101 MHz, $CDCl_3$): δ 147.4 (C^2), 145.7 (C^6), 137.4 (C^5), 125.7 (C^3), 123.7 (C^4), 33.6 ($\underline{C}(CH)_3$), 30.6 ($C(\underline{C}H)_3$), 17.3 ($\underline{C}H_3$); ESI-LRMS (+) *m/z* 166.2 $[M+H]^+$; ESI-HRMS (+) calcd for $C_{10}H_{16}NO$ 166.1232, found 166.1268.

(5-*tert*-Butylpyridin-2-yl)methanol (3).

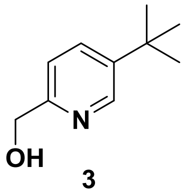

3

Trifluoroacetic anhydride (10 mL) was added to a solution of 5-*tert*-butyl-2-methylpyridine 1-oxide (450 mg, 2.73 mmol) in $CHCl_3$ (15 mL). The resulting mixture was heated at 60°C for 36 h under an inert atmosphere. After this time, the solvent was removed under reduced pressure and reaction completion to the trifluoroacetate intermediate was confirmed by ^{1}H NMR analysis. The resulting bright yellow oil was stirred in a mixture of EtOH (5 mL) and H_2O (5mL) for 1 h. The solution was concentrated (ca. 2 mL) and extracted with ethyl acetate (3 x 10 mL). The organic layers were combined, and washed with dil NaOH solution to remove the trifluoro acetic acid residues, dried over $MgSO_4$, and the solvent removed under reduced pressure to yield a yellow oil (396 mg, 88 %). R_f (10 % MeOH / DCM) = 0.42; ^{1}H NMR (400 MHz, $CDCl_3$): δ 8.57 (s, 1H, H^6), 7.71 (d, *J* = 8 Hz, 1H, H^4), 7.18 (d, *J* = 8 Hz, 1H, H^3), 4.77 (s, 2H, $C\underline{H}_2OH$), 1.34 (s, 9H, ^{t}Bu); ^{13}C NMR (101 MHz, $CDCl_3$): δ 153.4 (C^2), 148.5 (C^6), 141.6 (C^5), 137.8 (C^3), 123.7 (C^4), 59.5 ($\underline{C}H_2OH$), 33.4 ($\underline{C}(CH)_3$), 29.5 ($C(\underline{C}H)_3$); ESI-LRMS (+) *m/z* 166.2 $[M+H]^+$; ESI-HRMS (+) calcd for $C_{10}H_{16}NO$ 166.1232, found 166.1248.

(5-*tert*-Butylpyridin-2-yl)methyl methanesulfonate (4).

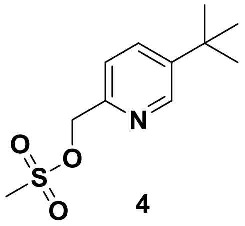

4

(5-*tert*-Butylpyridin-2-yl) methanol (396 mg, 2.40 mmol) was dissolved in THF (10 mL) and cooled to 5°C. Triethylamine (0.67 mL, 4.79 mmol) and mesyl chloride (0.28 mL, 3.60 mmol) were added dropwise to this solution. Once addition was complete, the reaction mixture was allowed to warm to rt and stirred for 2 h, before the solvent was removed under reduced pressure. The residue was treated with brine (10 mL) and extracted with DCM (2 x 10 mL). The organic layers were combined, dried over $MgSO_4$, and the solvent removed under reduced pressure to yield a pale orange oil, which was used immediately (530 mg, 91 %). R_f (10 % MeOH/DCM) = 0.76; ^{1}H NMR (400 MHz, $CDCl_3$): δ 8.68 (d, *J* = 2 Hz, 1H, H^6), 7.89 (dd, *J* = 8, 2 Hz, 1H, H^4), 7.52 (d, *J* = 8 Hz, 1H, H^3), 5.39 (s, 2H, C<u>H</u>$_2$OMs), 3.12 (s, 3H, SO_2C<u>H</u>$_3$), 1.37 (s, 9H, ^{t}Bu); ESI-LRMS (+) *m/z* 244.2 $[M+H]^+$; ESI-HRMS (+) calcd for $C_{11}H_{17}NO_3S$ 244.1007, found 244.1020

1,4,7-Tetraaza-cyclododecane-1,4,7-tricarboxylic acid tri-*tert*-butyl ester(5).

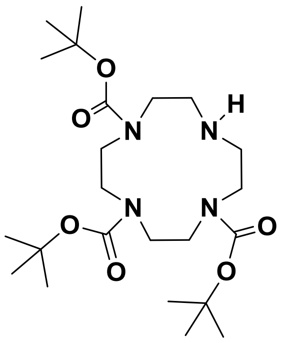

5

A solution of di-*tert*-butyl dicarbonate (10.1 g, 46.7 mmol) in chloroform (65 mL) was added drop wise over 2 h, at 0°C, to a stirred solution of cyclen (2.86 g, 15.6 mmol) and triethylamine (6.5 mL, 46.7 mmol) in chloroform (85 mL) under argon. The reaction mixture was then stirred at rt for 18 h before being washed with H_2O (2 x 100 mL). The organic phase was dried over Na_2SO_4, filtered, and the solvent removed under reduced pressure. The resulting white solid was purified by silica gel column chromatography, eluting with diethyl ether to yield a white solid (4.47 g, 61 %). R_f (Et_2O) = 0.21. 1H NMR (400 MHz, CDCl3): δ = 3.61 (br s, 4H, cyclen-CH_2), 3.33 (br s, 8H, cyclen-CH_2), 2.83 (br s, 4H, cyclen-CH_2), 1.46 (s, 9H, ^{t}Bu), 1.44 (s, 18H, ^{t}Bu); ^{13}C NMR (101 MHz, $CDCl_3$): δ = 155.6 (CO_2tBu), 155.4 (CO_2tBu), 79.3 ($C(CH_3)_3$), 79.2 ($C(CH_3)_3$), 51.0 (cyclen-CH_2), 49.5 (cyclen-CH_2), 46.0 (cyclen-CH_2), 45.0 (cyclen-CH_2), 28.7 ($C(CH_3)_3$), 28.5 ($C(CH_3)_3$); ESI/MS+ m/z 473.5 [M+H]+; HRMS Calcd for $C_{23}H_{45}N_4O_6$ 473.3323. Found 473.3339.

tert-Butyl-2,2',2''-(10-((5-tert-butylpyridin-2-yl)methyl)- ,4,7-tricarboxylic acid tri-tert-butyl ester (6)

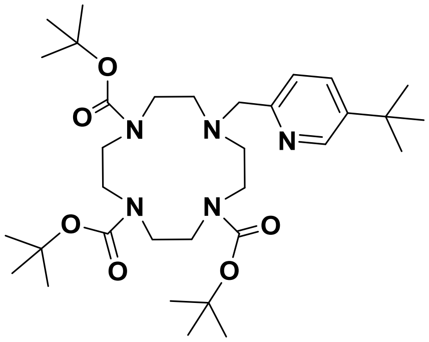

A stirred mixture of 1,4,7- tetraaza-cyclododecane-1,4,7-tricarboxylic acid tri-*tert*-butyl ester (0.35 g, 0.74 mmol), (5-*tert*-Butylpyridin-2-yl)methyl methanesulfonate (0.18 g, 0.74 mmol), and K_2CO_3 (0.11 g, 0.74 mmol) in anhydrous MeCN (20 mL) was refluxed for 18 h under argon. After this, the reaction mixture was cooled and filtered before the solvent was removed under reduced pressure. The resulting yellow oil was purified by silica gel column chromatography, eluting with a gradient starting from 100 % DCM to 2 % MeOH / DCM to yield a yellow sticky solid (0.27 g, 60 %) R_f (5 % MeOH / DCM) = 0.45. ^{1}H NMR (400 MHz, CDCl3): δ = 8.59 (s, 1H, H^6), 7.7.58 (d, *J* = 7 Hz, 1H, H^3), 7.18 (d, *J* = 7 Hz, 1H, H^4), 3.79 (s, 2H, NCH2py), 3.61-3.27 (br m, 12H, cyclen-CH2), 2.74 (br m, 4H, cyclen-CH2), 1.47 (s, 18H,^{t}Bu), 1.44 (s, 9H, ^{t}Bu), 1.32 (s, 9H, ^{t}Bu);^{13}C NMR (101 MHz, CDCl3): δ = 155.2 ($CO_2{}^t$Bu), 153.4 (C^2), 148.5 (C^6), 141.6 (C^5), 137.8 (C^3), 123.7 (C^4), 57.1 (NCH2py), 54.0 (cyclen-CH2), 53.2 (cyclen-CH2), 52.4 (cyclen-CH2), 49.0 (cyclen-CH2), 46.9 (cyclen-CH2), 33.4(C̲(CH3)3), 29.5 (C(C̲H)3), 27.7 (C(CH3)3), 27.4 (C(CH3)3); ESI/MS+ m/z 620.6 [M+H]+, HRMS Calcd for $C_{33}H_{58}N_5O_6$ 620.8502. Found 620.8496.

1-((5-tert-butyl pyridin-2-yl)methyl)-1,4,7,10-tetraazacyclododecane(7)

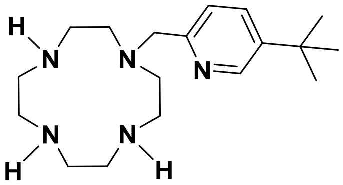

tert-Butyl-2,2',2''-(10-((5-*tert*-butylpyridin-2-yl)methyl)-1 ,4,7-tricarboxylic acid tri-*tert*-butyl ester (270 mg, 0.43 mmol) was dissolved in DCM (5 mL) with stirring. To this was added TFA (3 mL) and the mixture was stirred at rt for 18 h. After this time, TFA residues were removed by adding DCM (3x10mL) and removing under reduced pressure. The resulting oil was washed with diethyl ether. The residue was dissolved in 2 ml of water and the pH was adjusted to 10 using NaOH solution. The product was extracted using DCM (3 x 10 mL) and dried over $MgSO_4$. The solvent was removed under reduced pressure to yield a yellow oil (130 mg, 93 %).^{1}H NMR (400 MHz, $CDCl_3$): δ = 8.52 (d, *J* = 4 Hz, 1H, H^6), 7.65 (dd, *J* = 8, 4 Hz, 1H, H^4), 7.38 (d, *J* = 8 Hz, 1H, H^3), 3.74 (s, 2H, NCH_2py), 2.78 (m, 4H, cyclen-CH_2), 2.69 (m, 4H, cyclen-CH_2), 2.64 (m, 4H, cyclen-CH_2), 2.58 (m, 4H, cyclen-CH_2)1.30 (s, 9H, tBu); ^{13}C NMR (101 MHz,

CDCl3): δ = 152.7 (C^2), 142.7 (C^6), 140.4 (C^5), 130.0 (C^3), 118.3(C^4), 56.9 (N<u>C</u>H_2py), 47.7 (cyclen-<u>C</u>H_2), 43.5 (cyclen-<u>C</u>H_2), 42.8 (cyclen-<u>C</u>H_2), 41.4 (cyclen-<u>C</u>H_2), 29.6 (<u>C</u>$(CH)_3$), 27.3 (C(<u>C</u>$H_3)_3$), 27.2 (C(<u>C</u>$H_3)_3$); ESI/MS+ m/z 320.3 [M+H]+; HRMS Calcd for $C_{18}H_{34}N_5$ 320.2814, Found 320.2818.

Triethyl (10-((5-*tert*-Butyl pyridin-2-yl)methyl)-1,4,7,10- tetraazacyclododecane-1,4,7-triyl)tris(methylene)tris(methylphosphinate)

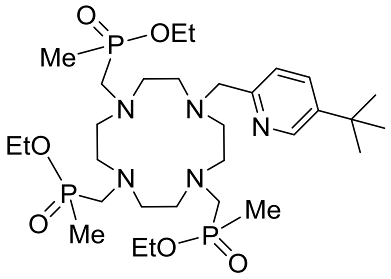

8

1-((5-(*tert*-Butyl)pyridin-2-yl)methyl)-1,4,7,10-tetraazacyclododecane (100 mg, 0.40 mmol) and paraformaldehyde (60 mg, 2.0 mmol) were taken in to anhydrous THF (30ml) and heated to 80°C under argon. Diethoxymethylphosphine (0.27 g, 2.0 mmol) were added before the solution was heated at reflux over molecular sieves for 15 h. Excess paraformaldehyde was removed by filtration and the solvent removed under reduced pressure. The resulting orange oil was purified using alumina, column chromatography, eluting with a gradient starting from 100 % DCM to 5 % MeOH / DCM, to yield a yellow oil (70 mg, 33 %), which consisted of a mixture of diastereoisomers. ^{1}H NMR (400 MHz, CDCl3): δ = 8.54 (s, 1H, H^6), 8.43 (d, *J* = 8 Hz, 1H, H^4), 7.48 (d, *J* = 8 Hz, 1H, H^3), 4.07 (m, 6H, POC<u>H</u>$_2CH_3$), 3.98 (s, 2H, NC<u>H</u>$_2$py), 3.84 (s, 2H, NC<u>H</u>$_2$P), 3.42 (br s, 4H, NC<u>H</u>$_2$P), 2.41-3.23 (br m, 16H, cyclen-C<u>H</u>$_2$), 1.39 (br m, 9H, PC<u>H</u>$_3$), 1.32 (s, 9H,C(C<u>H</u>$_3$)), 1.30 (m, 9H, OCH_2C<u>H</u>$_3$); ^{31}P NMR (162 MHz, $CDCl_3$) δ = 52.2, 52.0; ESI/MS+ m/z 680.3 [M+H]+; HRMS Calcd for $C_{30}H_{61}N_5O_6P_3$ 680.3835, Found 680.3837.

(10-((5-(*tert*-butyl)pyridin-2-yl)methyl)-1,4,7,10-tetraazacyclododecane- 1,4,7-triyl)tris(methylene)tris(methylphosphinic acid) (L^1)

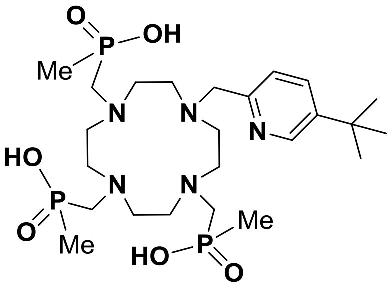

9

Triethyl-(10-((5-(tert-butyl)pyridin-2-yl)methyl)-1,4,7,10-tetraazacyclododecane-1,4,7-triyl)tris(methylene)tris(methylphosphinate) (60 mg, 0.087 mmol) was dissolved in NaOH (6 M, 5 mL)

and stirred for 18h at RT. The solvent was removed under reduced pressure and the residue washed repeatedly with DCM (3 x 5 mL) and the resulting oil was dissolved in water and pH was adjusted to 6.0 and the water was removed. The residue was taken into EtOH (5ml) and a white precipitate was removed by filtration and solvent removed under reduced pressure to yield a glassy solid (40 mg, 76 %).^{1}H NMR (400 MHz, D_2O): δ = 8.58 (s, 1H, H^6), 8.42 (s, 1H, H^4), 7.81 (s, 1H, H^3), 3.95 (s, 2H, NCH_2py), 3.42 (br s, 6H, NCH_2P), 3.32-2.71 (br m, 16H, cyclen-CH_2), 1.33 (m, 6H, PCH_3), 1.17 (m, 12H, PCH_3, $C(CH_3)_3$); ^{31}P NMR (162 MHz, D_2O) δ = 32.2; ESI/MS+ m/z 596.3 [M+H]+, HRMS Calcd for $C_{24}H_{49}N_5O_6P_3$ 596.2896, Found 596.2911, mp : dcs >160°C.

[Dy.L^8]

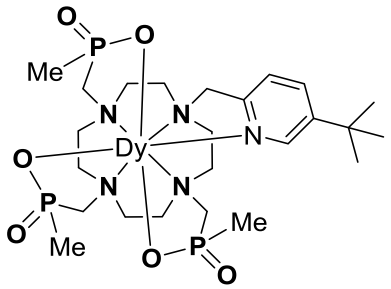

Dy(III)Cl_3.6H_2O (7 mg, 0.019 mmol) was added to a solution of (10-((5- (tert-butyl)pyridin-2-yl)methyl)-1,4,7,10-tetraazacyclododecane-1,4,7- riyl)tris(methylene)tris(methylphosphinic acid) (10mg, 0.016 mmol) dissolved in H_2O (3 mL). The pH was adjusted to 5.5 before stirring the solution for 18 h at 40°C. After this time, the solution was allowed to cool to rt before the pH was raised to 10, causing a white solid to precipitate out of solution. This precipitate was removed by centrifugation and the pH of the resulting solution neutralised using dil HCl. The solvent was removed under reduced pressure to yield a yellow solid that was purified by reverse-phase HPLC (12 mg, 98 %). HPLC :t_R=1.89 [Acquity UPLC BEH C18 :1.7µm (2.1mm x 50mm), water containing formic acid (0.1%v/v):Methanol, Flow rate 0.6 mL/min] ^{1}H NMR (400 MHz, D_2O, pD 6.9): δ = -75.0 major,-79.0 minor; ESI/MS+ m/z 757.87 $[M]^+$; HRMS Calcd for $C_{24}H_{45}{}^{160}DyN_5O_6P_3$ 753.1930. Found 753.1928.

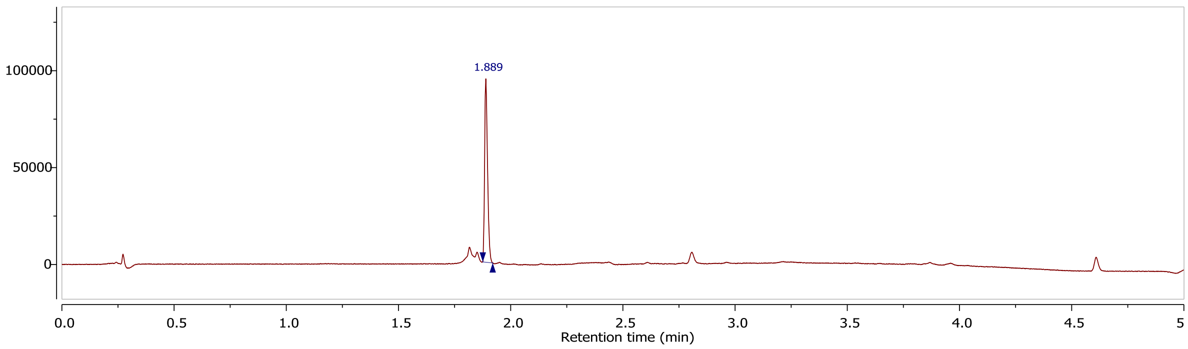

SI-Figure 2 : *HPLC chromatogram for [Dy.L^8], t_R = 1.89 min.*

[Tm.L[8]]

An analogous procedure to that described for **[Dy.L[1]]** was followed using (10-((5- (tert-butyl)pyridin-2-yl)methyl)-1,4,7,10-tetraazacyclododecane-1,4,7- triyl)tris(methylene)tris(methylphosphinic acid) (10 mg, 0.016 mmol) and Tm(III)Cl_3 (7.2 mg, 0.019 mmol) to yield a yellow solid (8 mg, 95 %).; ^{1} NMR (400 MHz, D_2O, pD 6.9): δ = 67 major, 68.3 minor ; ESI/MS+ m/z 762.2 [M]+,; HRMS Calcd for $C_{24}H_{45}N_5O_6P_3{}^{169}Tm$ 762.2003. Found 808.0838.

[Er.L[8]]

^{1}H NMR (400 MHz, D_2O, pD 6.9): δ = 38.2 major, 40.1 minor ; ESI/MS+ m/z 759.5 [M+1]+,; HRMS Calcd for $C_{24}H_{46}{}^{162}ErN_5O_6P_3$ 755.1949. Found 755.1954.

[Ho.L[8]]

^{1}H NMR (400 MHz, D_2O, pD 6.9): δ = - 31.8 major,-33.7 minor ; ESI/MS+ m/z 758.19 [M+1]+,; HRMS Calcd for $C_{24}H_{46}{}^{165}HoN_5O_6P_3$ 758.1964. Found 758.1971.

[Yb.L[8]]

^{1}H NMR (400 MHz, D_2O, pD 6.9): δ = - 16.3 major,18.1minor ; ESI/MS+ m/z 767.2 [M+1]+,; HRMS Calcd for $C_{24}H_{46}{}^{170}YbN_5O_6P_3$ 763. Found 763.2040.

[Eu.L[8]]

^{1}H NMR (400 MHz, D_2O, pD 6.9): δ = 5.3 major, 5.4 minor ; ESI/MS+ m/z 746.19 [M+1]+,; HRMS Calcd for $C_{24}H_{46}{}^{151}EuN_5O_6P_3$

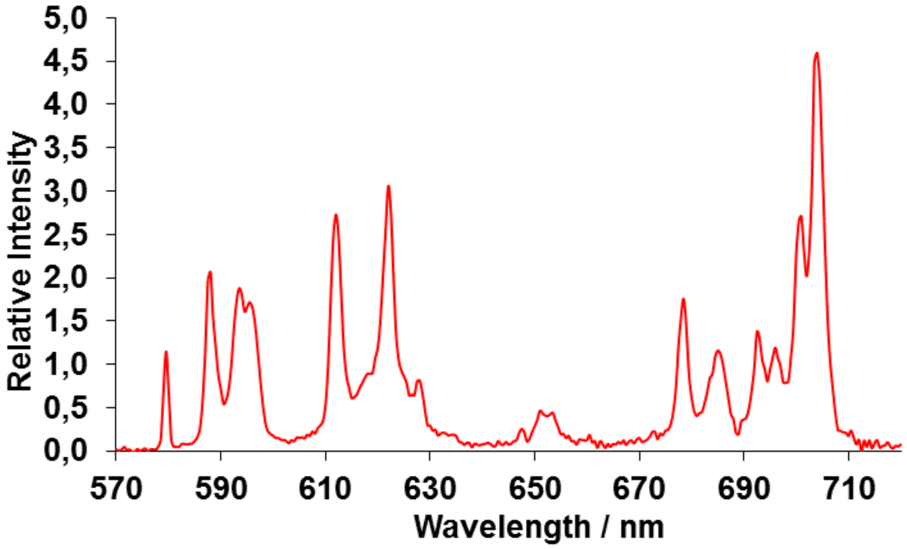

SI-Figure 3 : *Europium(III) emission spectrum (295 K, pD = 6) for [Eu.L[8]].*

[Tb.L[8]]

^{1}H NMR (400 MHz, D_2O, pD 6.9): δ = - 76.9 major,-77.9 minor ; ESI/MS+ m/z 752.18 [M+1]+,; HRMS Calcd for $C_{24}H_{46}N_5O_6P_3{}^{159}Tb$ 752.1914. Found 752.1895. τ_{H2O}= 3.23 ms and . $_{D2O}$ = 3.63 ms.

3. Chemical shift data of lanthanide(III) complexes

The following section is sorted by the number of the complex series. Starting from [Ln.L[1]].

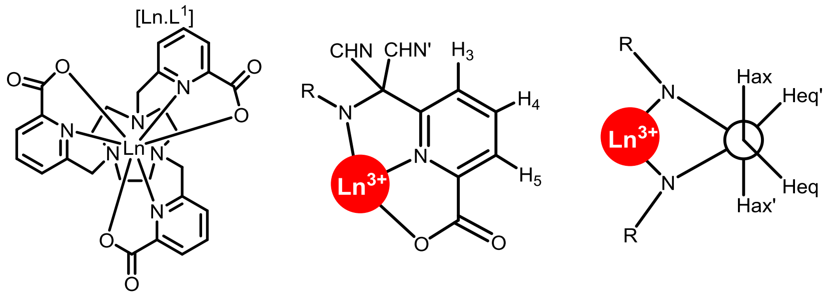

Scheme 2 : *Structure and resonances of interest of [Ln.L[1]].*

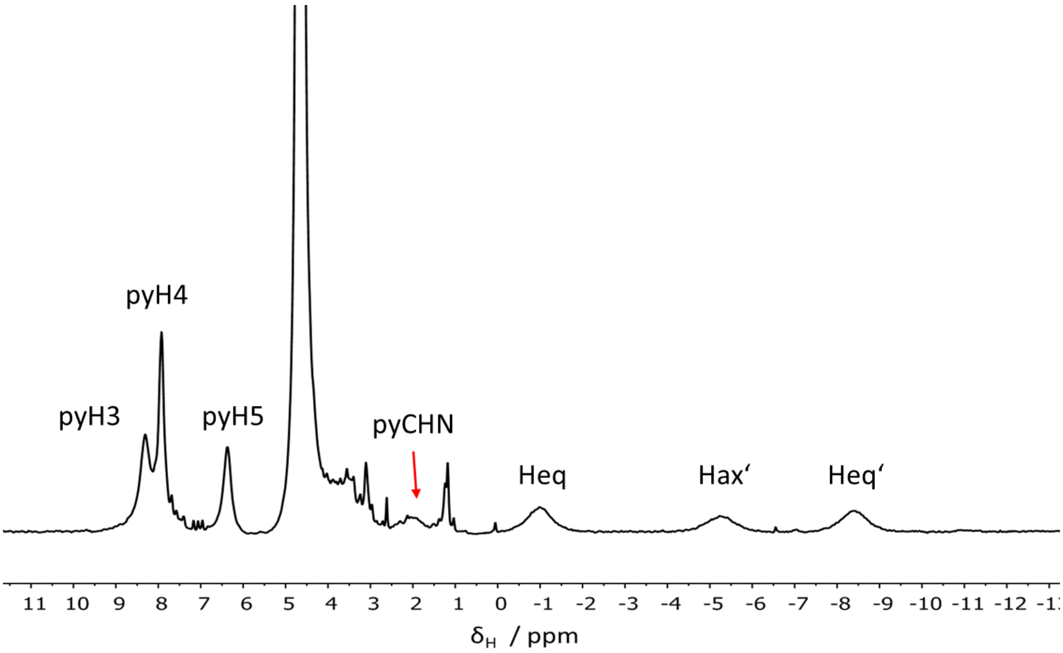

SI-Figure 4 : *^1H NMR spectrum of [Er.L[1]] (295 K, D_2O, pD = 6).*

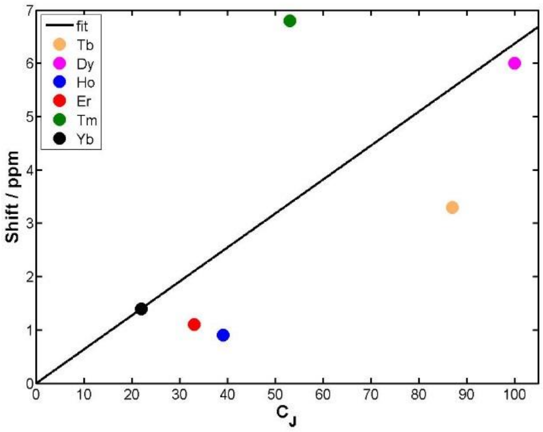

SI-Figure 5 : *Correlation of the pseudocontact shift data of the pyH[4] resonance for [Ln.L[1]] with the Bleaney constant, C_J (295 K, 9.4 T, D_2O).*

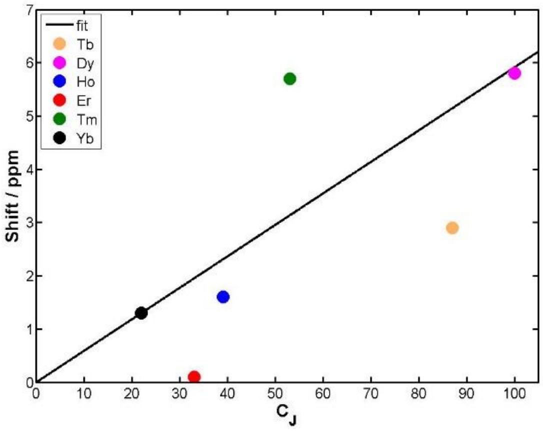

SI-Figure 6: *Correlation of the pseudocontact shift data of the pyH[5] resonance for [Ln.L[1]] with the Bleaney constant, C_J (295 K, 9.4 T, D_2O).*

SI-Table 2 : ^{1}H NMR c*hemical shift data for the resonances of [Ln.L[1]] (295 K, 9.4 T, D_2O)*

Ln^{3+}	Δδ / ppm								
	Hax	Hax’	Heq	Heq’	CHN	CHN’	H^3	H^4	H^5
Tb	-5.2	x	x	x	x	x	0.1	4.9	4.1
Dy	-13.6	26.5	x	-4.9	-24.0	-29.4	9.4	10.6	11.0
Ho	x	x	x	x	2.0	-9.0	3.9	6.2	6.5
Er	x	-5.6	-1.2	-8.6	1.9	12.4	8.3	7.9	6.3
Tm	11.5	-35.9	-13.1	-15.2	5.6	34.7	14.2	13.5	14.2
Yb	5.8	-4.8	0.6	-1.8	4.2	11.1	9.5	9.1	8.8
Y	2.7-3.5				4.1	4.4	8.0	7.8	7.4

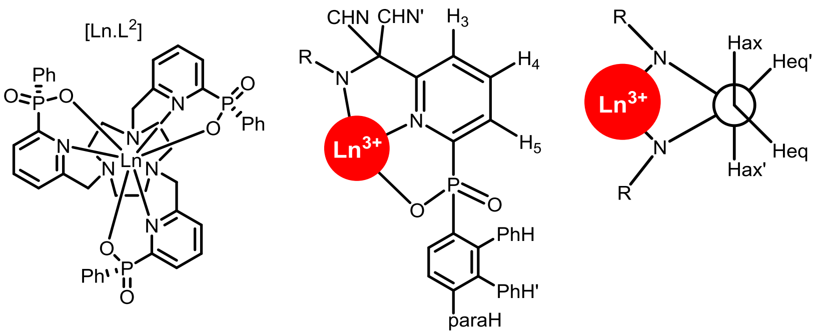

Scheme 3 : *Structure and resonances of interest of [Ln.L^2].*

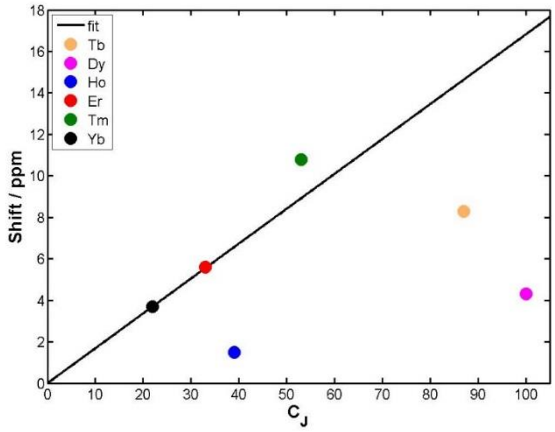

SI-Figure 7 : *Correlation of the pseudocontact shift data of the pyH^4 resonance for [Ln.L^2] with the Bleaney constant, C_J (295 K, 9.4 T, CD_3OD).*

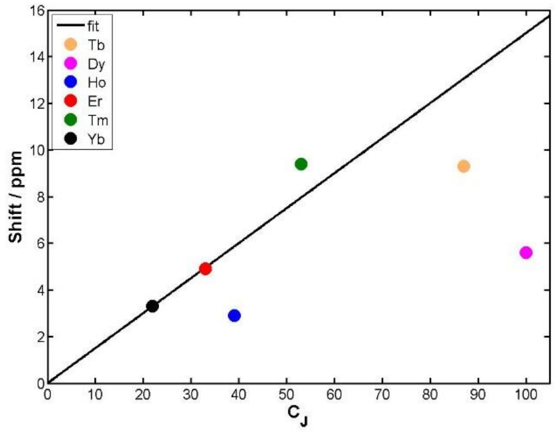

SI-Figure 8 : *Correlation of the pseudocontact shift data of the pyH^5 resonance for [Ln.L^2] with the Bleaney constant, C_J (295 K, 9.4 T, CD_3OD).*

SI-Table 3 : ^{1}H NMR *chemical shift data for the resonances of [Ln.L^{2}] (295 K, 9.4 T, CD$_3$OD)*

Ln^{3+}	δH / ppm											
	Hax	Hax'	Heq	Heq'	CHN	CHN'	pyH^3	pyH^4	pyH^5	ph	ph'	^{31}P
Tb	30.9	47.7	-11.6	x	19.2	-38.1	-7.1	-2.3	-1.3	13.2	14.7	-35.7
Dy	29.5	20.1	-6.2	x	x	-23.8	-1.4	1.4	2.7	10.8	x	-15.9
Ho	15.4	13.8	-2.4	-2.7	6.6	-8.4	2.3	4.1	5.5	9.0	x	-24.6
Er	35.8	-32.2	-13.5	x	-17.7	35.8	13.6	11.9	12.6	3.2	1.0	-10.5
Tm	11.4	-56.9	-22.7	-23.0	-21.7	54.6	18.6	16.4	17.8	6.5	1.0	8.4
Yb	6.6	-13.7	-3.4	5.2	-0.6	20.7	10.7	10.3	10.7	5.2	3.8	17.7
Y	3.62	2.65	2.86	2.65	4.95	4.1	8.0	7.8	7.4	6.7	7.9	23.9

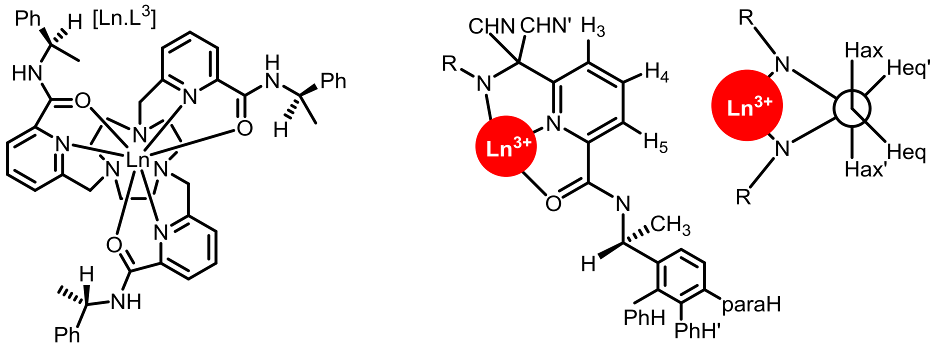

Scheme 4 : *Structure and resonances of interest of [Ln.L^{3}]$^{3+}$.*

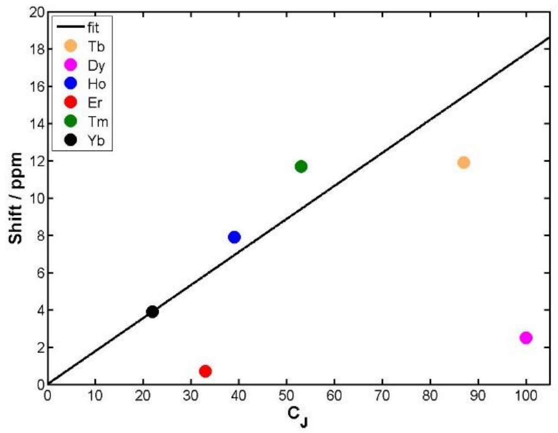

SI-Figure 9 : *Correlation of the pseudocontact shift data of the pyH^4 resonance for [Ln.L^{3}]$^{3+}$ with the Bleaney constant, C_J (295 K, 9.4 T, CD$_3$OD).*

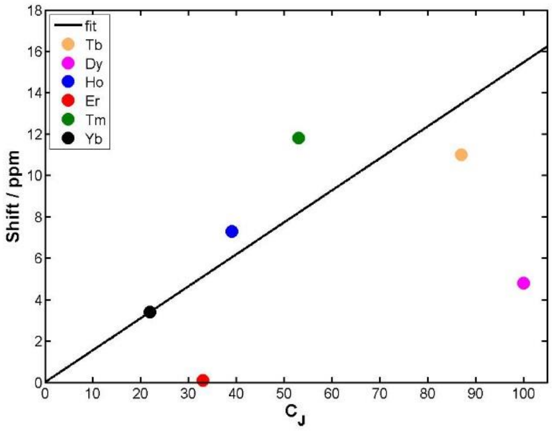

SI-Figure 10 : *Correlation of the pseudocontact shift data of the pyH^5 resonance for [Ln.L^3]$^{3+}$ with the Bleaney constant, C_J (295 K, 9.4 T, CD_3OD).*

SI-Table 4 : ^{1}H NMR c*hemical shift data for the resonances for [Ln.L^3]$^{3+}$ (295 K, 9.4 T, CD_3OD)*

Ln^{3+}	Δδ / ppm												
	H_{ax}	H_{ax}'	H_{eq}	H_{eq}'	CHN	CHN'	pyH^3	pyH^4	pyH^5	PhH	PhH'	CH_3	H
Tb	x	57.5	29.9	23.9	x	-45.5	-11.0	-3.2	-4.4	4.5	2.0	8.6	5.8
Dy	22.5	x	18.2	11.1	x	x	1.9	5.0	3.0	6.5	6.2	x	-1.5
Ho	-3.7	47.3	23.3	22.6	x	-32.0	-5.5	-0.4	0.5	5.5	5.1	7.2	5.8
Er	3.6	-5.1	-8.8	x	1.8	12.8	8.2	7.9	8.2	x	x	0.9	x
Tm	11.3	-79.8	-29.0	-30.5	-22.0	70.4	23.0	19.6	19.2	10.4	10.8	-7.5	9.3
Yb	8.9	-18.8	-4.2	-6.7	-2.8	22.3	11.6	11.2	11.4	7.6	8.2	-1.1	x
Y	2.4-2.8				3.2		8.0	7.8	7.4	7.2-7.4		1.62	5.4

SI-Table 5 : *Variation of the chemical shift of the quoted resonance for [Yb.L^3]$^{3+}$ with temperature over the given range (16.5 T, D_2O).*

T / K	δ_H / ppm			
	pyH^3	pyH^4	pyH^5	$H_{ax'}$
298	12.3	12.0	11.7	-23.6
313	12.0	11.8	11.5	-20.8
323	11.8	11.7	11.4	-19.2
333	11.6	11.6	11.3	-17.8
343	11.5	11.5	11.3	-16.5
353	11.4	11.5	11.2	-15.3
ppm / K	n.d.	n.d.	n.d.	-0.2

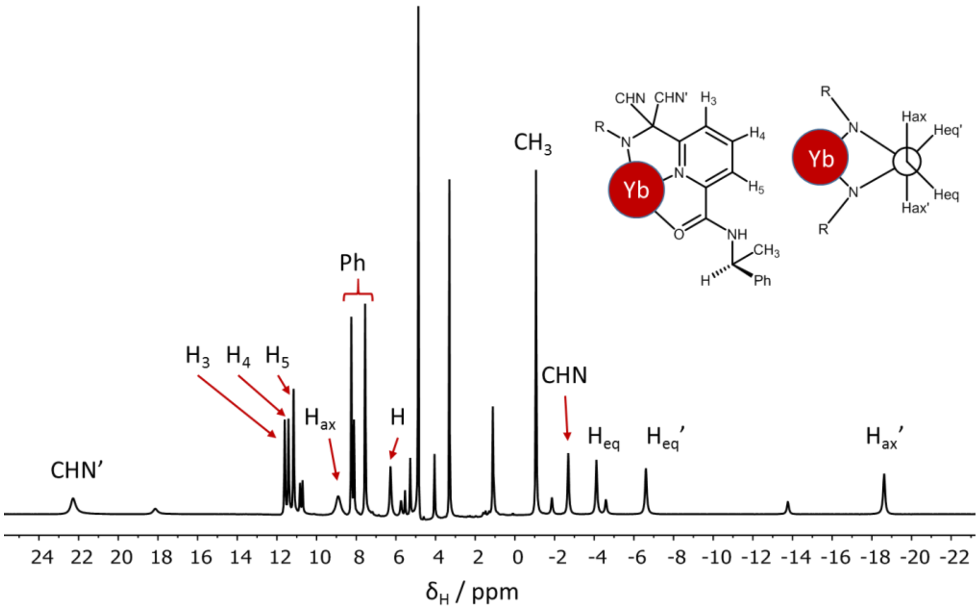

SI-Figure 11 : *Annotated ^{1}H NMR spectrum of (S)-[Yb.L^{3}]$^{3+}$ in CD_3OD, (295 K, 9.4 T). Unassigned resonances relate to the minor isomer.*

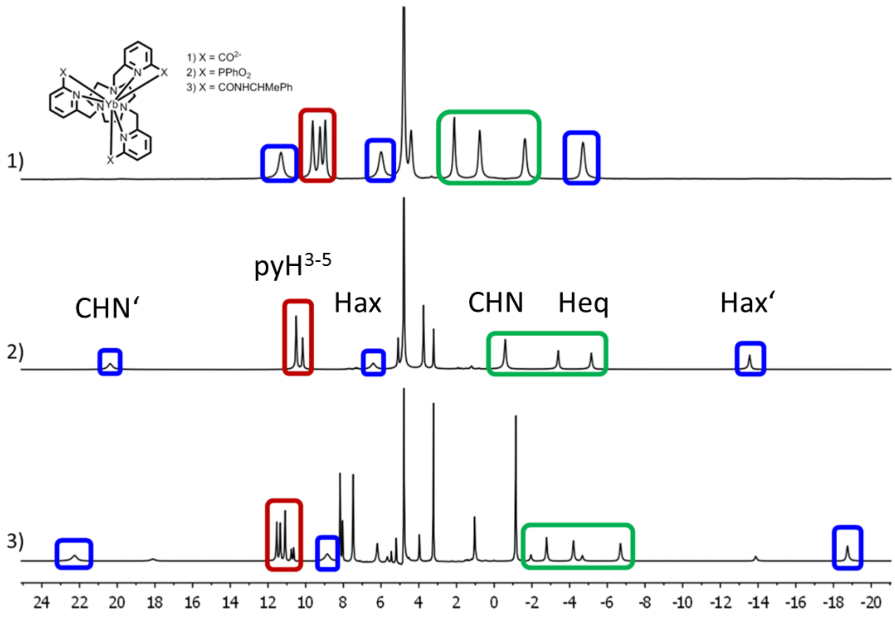

SI-Figure 12 : *^{1}H NMR spectrum of [Yb.L^{1}] (upper), [Yb.L^{2}] and [Yb.L^{3}]$^{3+}$ (lower) showing the variation of shift dispersion with B_0^2, with 75, 110 and 235 cm^{-1} respectively (295K, 9.4 T, CD_3OD, [Ln.L^{1}] in D_2O).*

It is very important to make sure that the lanthanide(III) complexes of a given ligand form an isostructural series. There are multiple methods to ensure that an isostructural series is formed, for example, investigating the X-ray crystallographic structures, if available. One of the simplest ways is to analyse the chemical shift data. In axial symmetric complexes it is possible to simplify the equation of the overall dipolar shift as follows:

$$\delta_{ij}^{para} = F_i\langle S_z\rangle + C_J B_0^2 G_i$$

where *i* is the nucleus of interest of the complex of the lanthanide(III) ion, *j* and B_0^2 is the second order crystal field splitting parameter. F_i is the contact term, C_J the Bleaney constant, and G_i the geometrical term and are defined as follows:

$$F_i = \frac{A_i}{\hbar\,\gamma_i\,H_0} \qquad\qquad G_i = \frac{3cos^2\theta - 1}{r^3}$$

$$C_J = g_J^2\langle J\,\|\alpha\|\,J\rangle J(J + 1(2J + 3)(1 + p)$$

where *A* is the hyperfine coupling constant, γ_i the magnetogyric ratio, H_0 the magnetic field strength, θ the angle to the principal magnetic axis, *r* the internuclear distance to the lanthanide(III) ion and a $\langle J\,\|\alpha\|\,J\rangle$ numerical coefficient calculated by Bleaney. Assuming that both, <Sz> and Cj are not modulated by the crystal field splitting it is possible to create Reilley[16] plots (plotting $\frac{\delta_j^{para}}{<S_z>}\,vs\,\frac{C_j}{<S_z>}$) across a series of lanthanide(III) complexes that should show a linear trend, if the series is isostructural. A discrepancy in the linear behaviour would indicate structural changes across the series of complexes.

However, we propose, in this work, that C_j does vary with the coordination environment. The two-nuclei method proposed by Reuben[17] analyses two nuclei of a lanthanide(III) complex across the series, so that the plots are independent of C_j and B_0^2. Plotting $\frac{\delta_{ij}^{para}}{<S_z>}\,vs\,\frac{\delta_{kj}^{para}}{<S_z>}$ should, also give a straight line across an isostructural series of lanthanide(III) complexes. This method removes the crystal field splitting and Bleaney constant from the equation. The gradient of the plot is then simply dependent

on the geometrical factor G_i, and the intercept indicates the hyperfine coupling constant, *A*.[18]

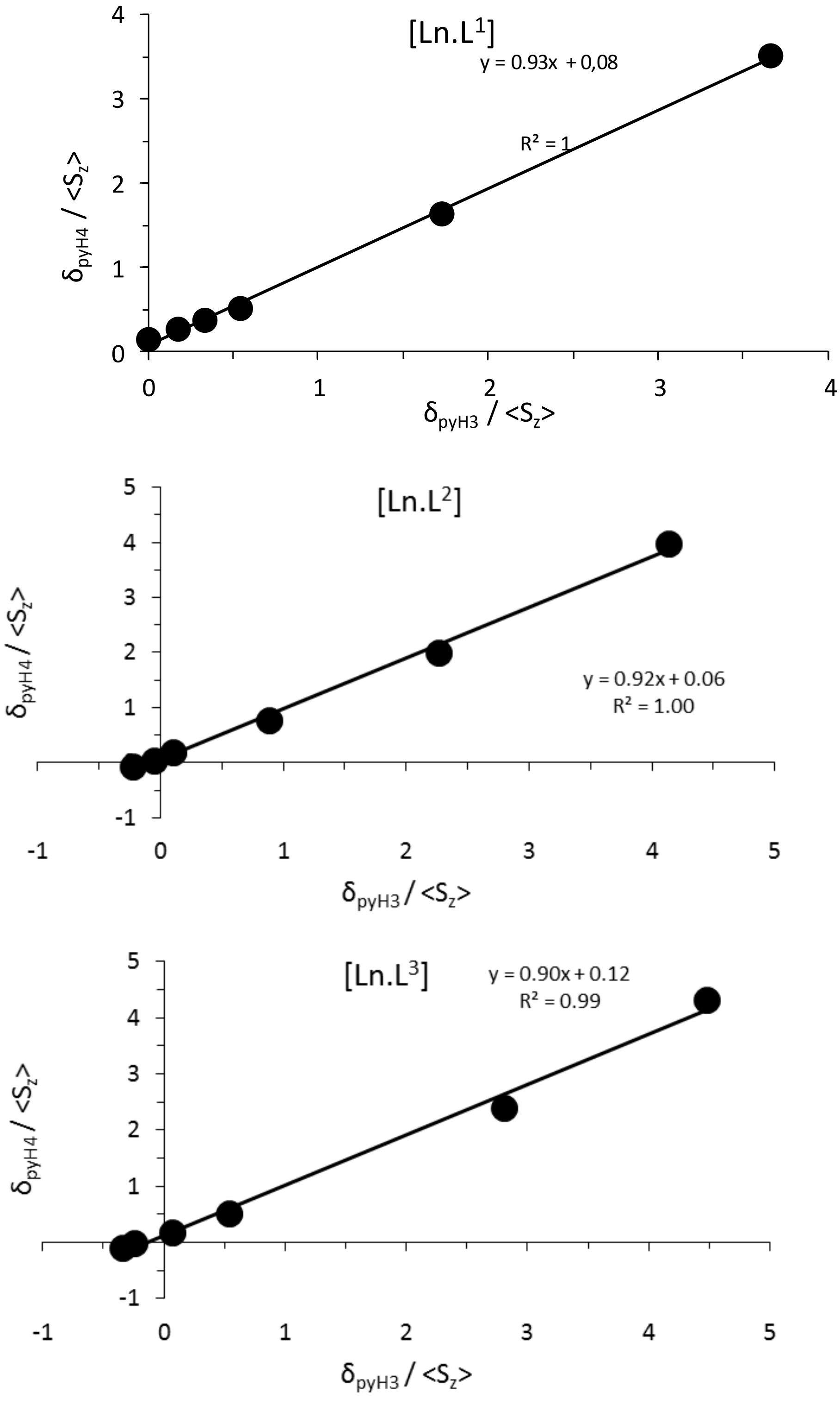

SI-Figure 13 *Two-nuclei Reilley plots of the chemical shifts of pyH^3 and pyH^4 of [Ln.L^1] to [Ln.L^3] showing a linear correlation across the series (9.4 T, 295 K, [Ln.L^1] in D_2O, [Ln.L^{2,3}] in CD_3OD.*

In each case (Fig.SI-1, above) a very good linear correlation is present between the different lanthanide(III) ions consistent with an isostructural series. The intercept is near zero, indicating a very small contact shift contribution in every case.

This principle can be extended to the three nuclei method proposed by Geraldes[19], removing $<S_z>$ from the equation by plotting $\frac{\delta_{ij}^{para}}{\delta_{kj}^{para}}\ vs\ \frac{\delta_{lj}^{para}}{\delta_{kj}^{para}}$. An example of this method is given in SI Figure 2.

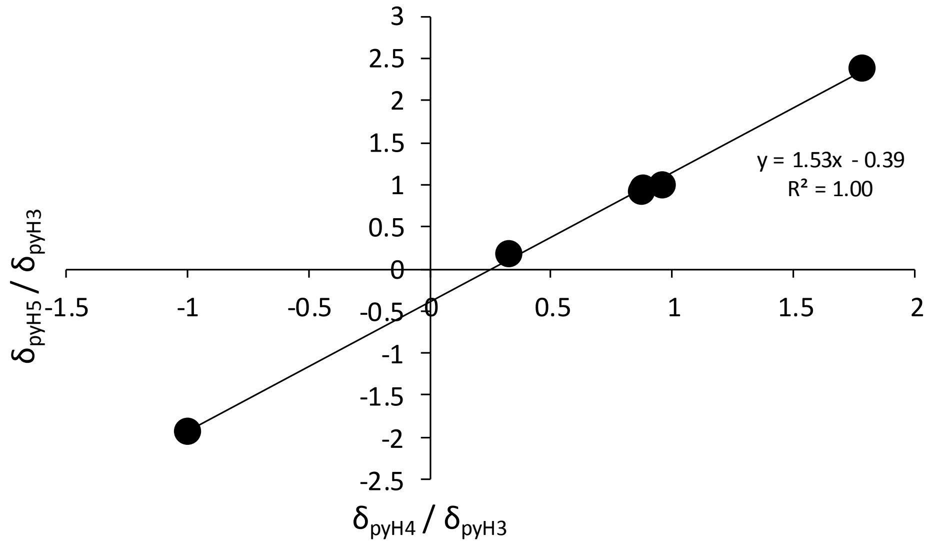

SI-Figure 14 *Three nuclei plots of the chemical shift of pyH[3,4,5] for the [Ln.L[2]] series, 9.4 T, 295 K, CD_3OD.*

Similar to the two nuclei plots, the [Ln.L[2]] series shows a very good linear correlation, consistent with the isostructurality of the 9-coordinate series. The corresponding plots for [Ln.L[1]] and [Ln.L[3]] revealed similar behaviour, but the correlation coefficient was not as high(R^2 = 0.94 and 0.84 respectively). The slope and intercept in these cases are rather complex non-linear combinations of the geometrical factor and the contact shift term and are difficult to analyse and interpret.

SI-Table 6: *Ratios of the chemical shift of the pyH[3] and the pyH[4] resonance of [Ln.L[1]] over the expectation value $<S_z>$ (295 K, 9.4 T, D_2O).*

Ln^{3+}	δ_{pyH3} / <S_z>	δ_{pyH4} / <S_z>
Tb	0.0031	0.1540
Dy	0.3293	0.3713
Ho	0.1723	0.2740
Er	0.5400	0.5140
Tm	1.7296	1.6443
Yb	3.6680	3.5135

SI-Table 7 *Ratios of the chemical shift of the pyH[3] and the pyH[4] resonance of [Ln.L[2]] over the expectation value <S_z> (295 K, 9.4 T, CD_3OD).*

Ln^{3+}	δ_{pyH3} / <S_z>	δ_{pyH4} / <S_z>
Tb	-0.2231	-0.0723
Dy	-0.0490	0.0490
Ho	0.1016	0.1811
Er	0.8848	0.7742
Tm	2.2655	1.9976
Yb	4.1313	3.9768

SI-Table 8: *Ratios of the chemical shift of the pyH[3] and the pyH[4] resonance of [Ln.L[3]] over the expectation value <S_z> (295 K, 9.4 T, CD_3OD).*

Ln^{3+}	δ_{pyH3} / <S_z>	δ_{pyH4} / <S_z>
Tb	-0.3457	-0.1006
Dy	0.0666	0.1751
Ho	-0.2430	-0.0177
Er	0.5335	0.5140
Tm	2.8015	2.3873
Yb	4.4788	4.3243

SI-Table 9 *Ratios of the chemical shift of the pyH[4] and the pyH[5] resonance of [Ln.L[3]] over the chemical shift of the pyH[3] resonance (295 K, 9.4 T, CD_3OD).*

Ln^{3+}	δ_{pyH4} / δ_{pyH3}	δ_{pyH5} / δ_{pyH3}
Tb	0.3239	0.1831
Dy	-1.0000	-1.9286
Ho	1.7826	2.3913
Er	0.8750	0.9265
Tm	0.8817	0.9570
Yb	0.9626	1.0000

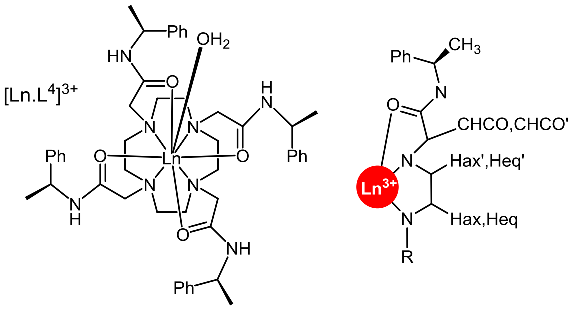

Scheme 5 : *Structure and resonances of interest of $[Ln.L^4]^{3+}$.*

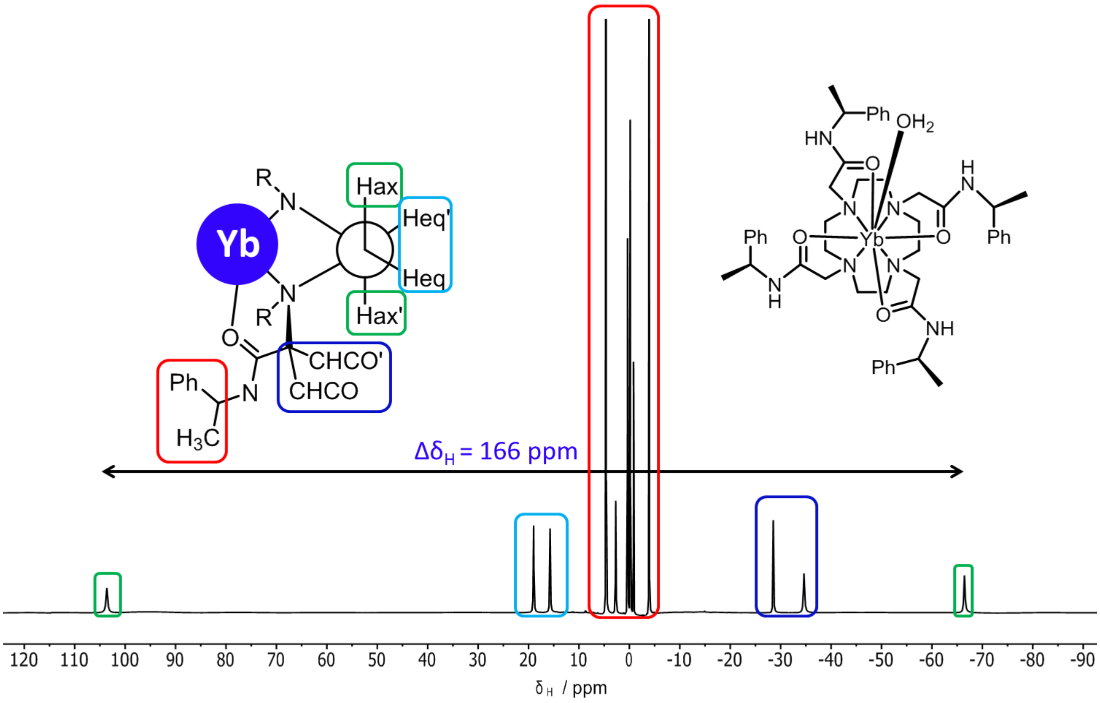

SI-Figure 15 : *Model paramagnetic NMR spectrum of a C_4 symmetric Yb(III) complex, with shifted resonances highlighted (295 K, D_2O, 9.4 T).*

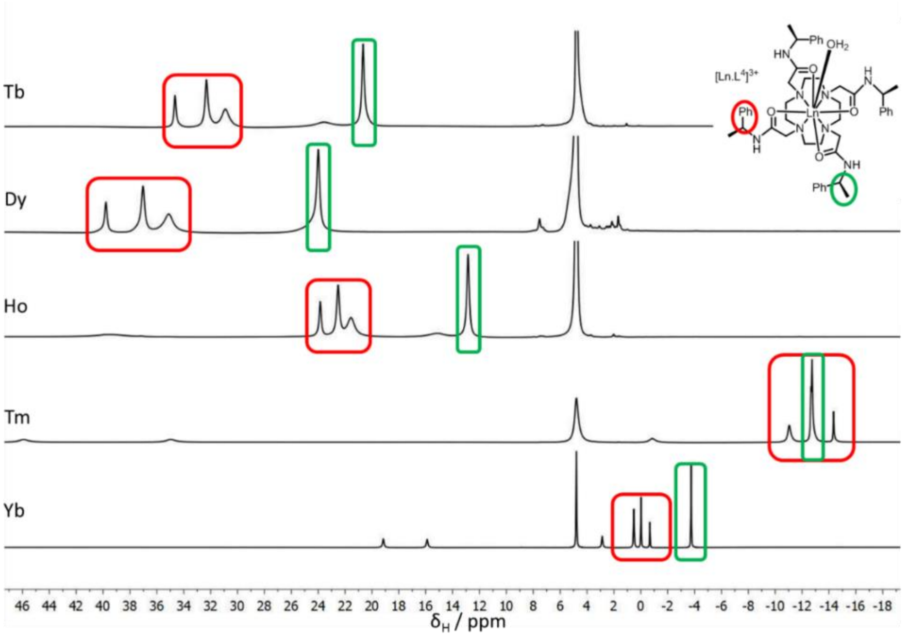

SI-Figure 16 : *Part of the ^{1}H spectrum some selected [Ln.L^4]$^{3+}$ complexes in which the phenyl and methyl resonances are highlighted. The different shift dispersion and line widths of the ligand resonances for the different lanthanide(III) ions are apparent (D_2O, 11.7 T, 295 K).*

SI-Table 10 : ^{1}H NMR c*hemical shift data for the resonances for [Ln.L^4]$^{3+}$ (295 K, 9.4 T, D_2O).*

Ln^{3+}	Δδ / ppm									
	Hax	Hax'	Heq	Heq'	CHCO	CHCO'	ph	ph'	paraH	CH_3
Tb	-341.7	223.9	x	-88.5	61.8	112.3	34.9	32.5	31.1	20.8
Dy	-400.4	270.8	-91.0	-95.0	87.5	137.8	40.4	37.6	35.6	24.4
Ho	-202.4	137.5	-49.8	-51.2	39.7	70.8	24.0	22.7	15.1	12.9
Er	89.5	-73.5	15.0	x	-39.2	-42.0	-0.2	-0.8	-1.6	-4.5
Tm	225.5	-182.6	34.9	45.9	-82.7	-95.7	-14.8	x	-11.4	-13.1
Yb	103.3	-66.2	15.7	19.0	-28.4	-34.5	-0.8	0.1	2.7	-3.9
Y	2.5-2.8				2.1	2.2	7.3		7.0	1.5

SI-Table 11 : *Variation of the chemical shift of the quoted resonance for [Tm.L^4]$^{3+}$ with temperature over the given range (11.7, CD_3OD).*

T / K	δ_H / ppm								
	Heq'	Heq	PhH	PhH2	paraH	CH3	CHCO	CHCO'	Hax
241	69.7	54.1	-22.2	-21.7	-23.7	-18.7	-118.7	-137.9	-269.1
251	64.4	49.9	-19.9	-19.6	-21.2	-17.1	-110.0	-127.9	-249.1
261	59.8	46.3	-17.8	-17.8	-19.0	-15.4	-102.2	-118.5	-230.9
273	55.6	43.0	-15.9	-15.9	-17.0	-14.3	-95.1	-110.2	-214.5
283	51.8	40.0	-14.2	-14.5	-15.2	-13.2	-88.7	-102.7	-199.6
298	46.9	36.1	-12.0	-12.5	-12.8	-11.6	-80.3	-92.7	-180.0
308	44.0	33.8	-10.7	-11.4	-11.4	-10.7	-75.4	-87.1	-168.7
319	41.4	31.8	-9.5	-10.3	-10.2	-9.8	-70.9	-81.7	-158.3
ppm / K	-0.4	-0.3	0.2	0.2	0.2	0.1	0.6	0.7	1.4

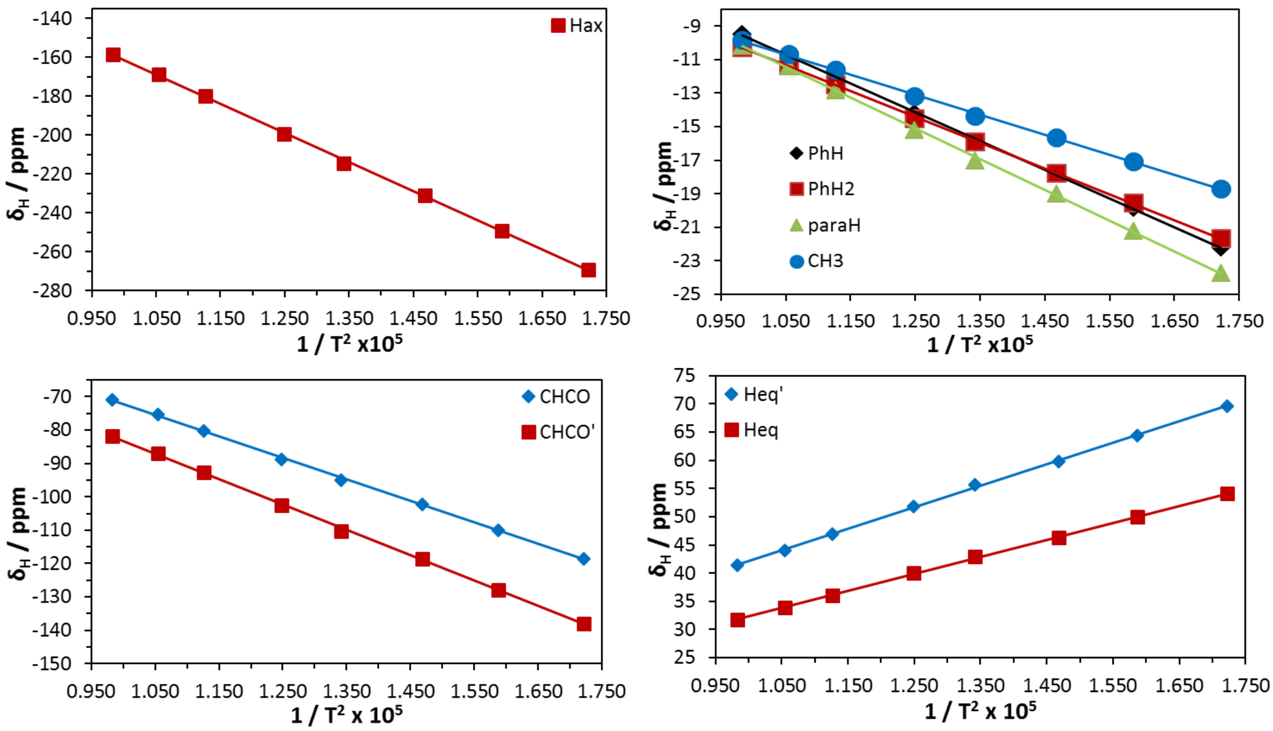

SI-Figure 17 : *Variation of the chemical shift of various resonances of $[Tm.L^4]^{3+}$ with $1 / T^2$ from 241 to 319 K, (CD_3OD, 11. 7 T). A steeper gradient indicates a bigger shift in ppm / K.*

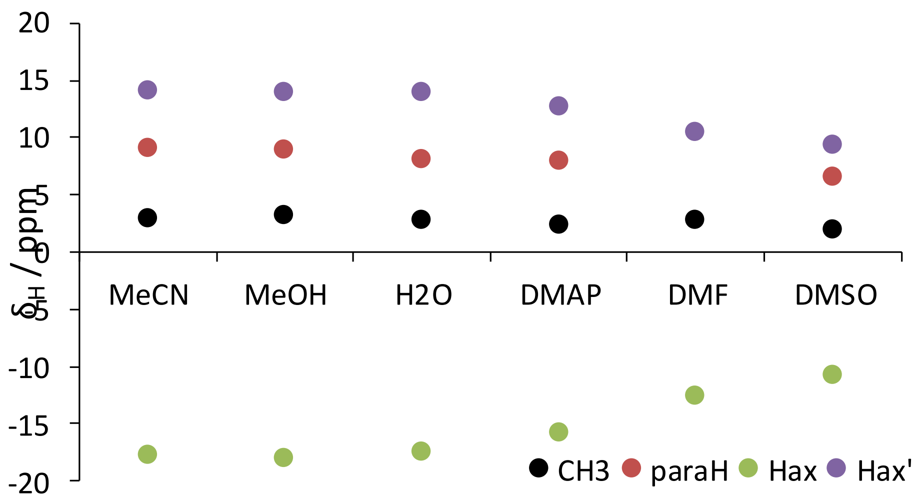

SI-Figure 18 : *Changes in chemical shift data for selected ^1H resonances of $[Ce.L^4]^{3+}$ with the polarizability of the axial donor group (CH_3CN, 295 K, 4.7 T).*

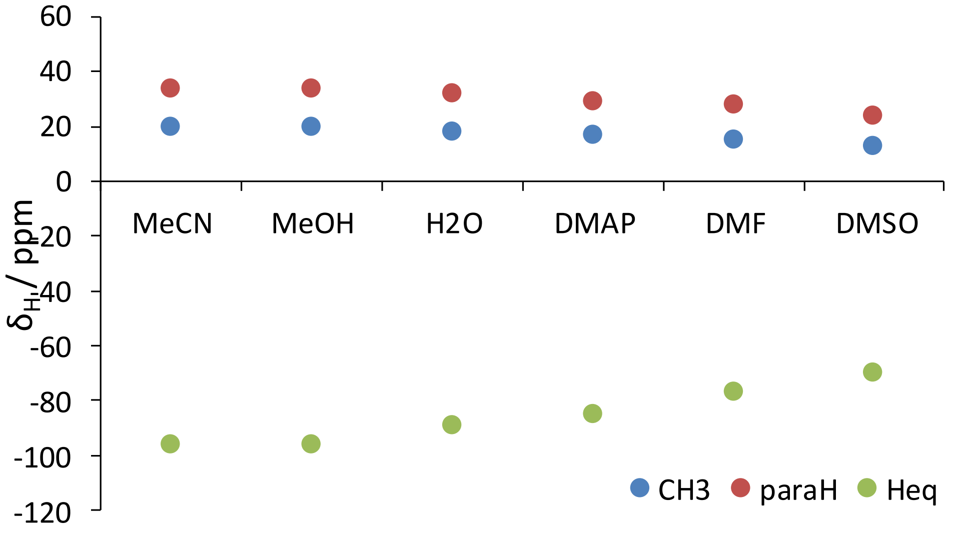

SI-Figure 19 : *Changes in chemical shift data for selected ^{1}H resonances of [Tb.L[4]][3+] with the polarizability of the axial donor group (CH_3CN, 295 K, 4.7 T).*

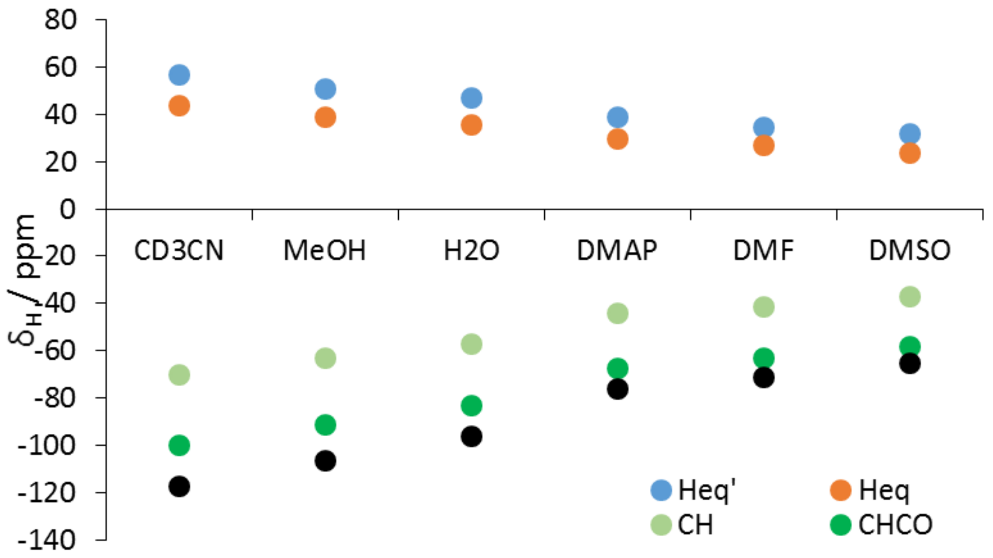

SI-Figure 20 : *Changes in chemical shift data for selected ^{1}H resonances of [Tm.L[4]][3+] with the polarizability of the axial donor group (CH_3CN, 295 K, 4.7 T).*

SI-Table 12 : *Overview of the average percentage change of the resonances of the [Ln.L^{4}]$^{3+}$ with changing axial donor group in order of donor polarizability (CH_3CN, 4.7 T, 295 K).*

Donor	Ce	Eu[a]	Tb	Tm	Yb[a]
MeCN	100	100	100	100	100
MeOH	101.6	88.7	100.2	89.9	96.1
Water	94.9	89.3	93.6	82.2	86.7
DMAP	86.4	70.2	87.0	66.3	76.7
DMF	79.3	66.5	79.7	61.0	75.3
DMSO	66.0	55.4	69.7	55.4	60.6

[a]Taken from [20]

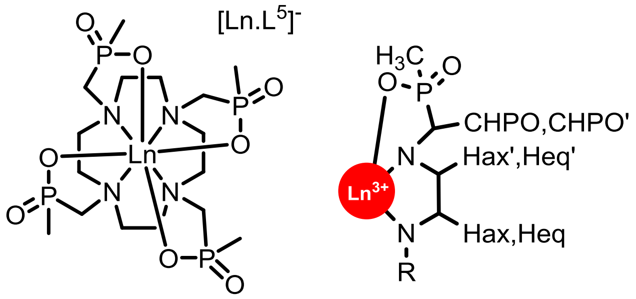

Scheme 6 : *Structure and resonances of interest of [Ln.L^{5}]$^{-}$.*

SI-Table 13 : ^{1}H NMR *chemical shift data for the resonances for [Ln.L^{5}]$^{-}$ (295 K, 9.4 T, D_2O).*

Ln^{3+}	Δδ / ppm						
	Hax	Hax'	Heq	Heq'	CHPO	CHPO'	CH_3
Tb	190.2	380.0	-105.8	-166.1	135.1	-450.0	42.1
Dy	320.3	163.0	-87.6	-93.5	115.2	-428.7	35.0
Ho	151.4	79.1	-42.7	-44.5	52.0	-198.7	15.7
Er	-93.8	-185.2	26.7	43.4	-79.2	260.0	-14.4
Tm	-193.7	-374.9	71.0	91.3	-154.7	305.6	-27.2
Yb	-30.9	-59.3	17.7	13.2	-24.7	90.9	-3.4
Y	2.2-2.4				3.1	3.1	1.4

SI-Table 14 : *Variation of the chemical shift of the quoted resonance for [Tm.L^5]$^-$ with temperature over the given range (16.5 T, D_2O).*

T / K	δ_H / ppm				
	Hax	CHPO	CH3	Heq’	Heq
298	-189.9	-151.9	-26.8	89.8	69.7
301	-186.4	-149.1	-26.4	88.2	68.5
304	-182.8	-146.3	-25.9	86.7	67.3
307	-179.3	-143.5	-25.4	85.2	66.1
310	-175.8	-140.8	-25.0	83.7	65.0
313	-172.5	-138.2	-24.5	82.3	63.9
316	-169.2	-135.6	-24.1	80.9	62.8
319	-165.8	-133.1	-23.7	79.5	61.7
321	-162.9	-130.8	-23.3	78.1	60.6
ppm / K	1.2	0.9	0.2	-0.5	-0.4

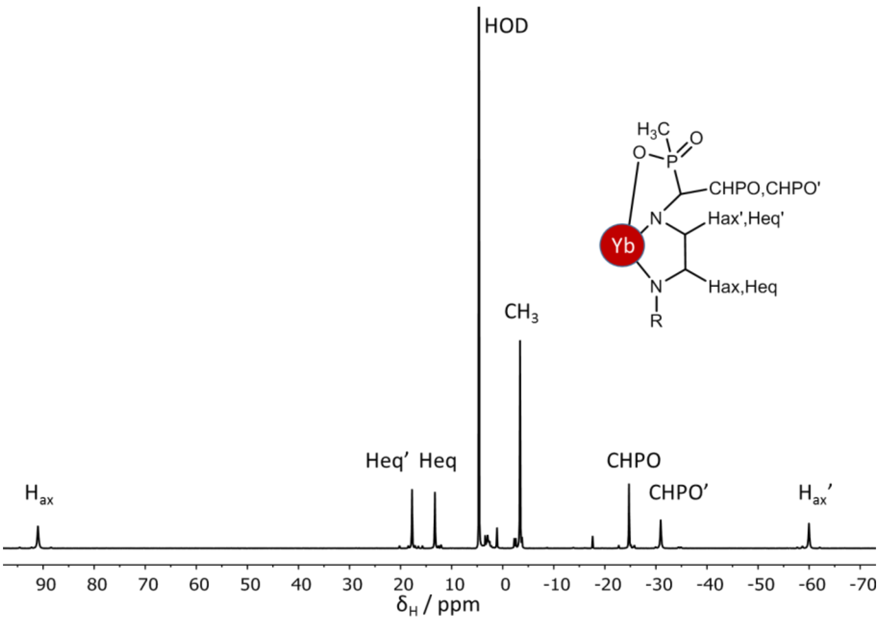

SI-Figure 21 : *Annotated ^1H NMR spectrum of [Yb.L^5]$^-$ (D_2O, 295 K, 9.4 T). Unassigned resonances relate to a minor isomer.*

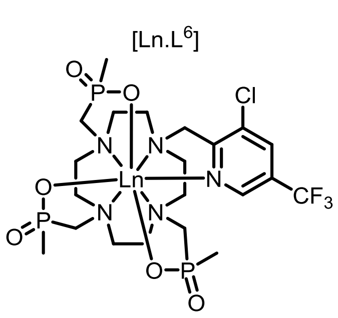

Scheme 7 : *Structure of [Ln.L^6].*

SI-Table 15 : ^{1}H NMR *chemical shift data for the resonances of [Ln.L^{6}] (295 K, 9.4 T, D_2O).*

Ln^{3+}	Δδ / ppm			
	CH_3	CH_3'	CH_3''	^{19}F
Tb	26.0	58.1	124.6	-158.4
Dy	71.4	89.9	113.5	-162.4
Ho	15.3	45.2	x	-107.8
Er	-37.2	-40.1	x	-16.9
Tm	-36.7	-37.1	-84.5	17.1
Yb	-6.9	-15.3	-29.8	-41.5
Y	1.5	1.5	1.5	-64.0

SI-Table 16 : *Variation of the chemical shift of the quoted resonance of [Dy.L^{6}] with temperature over the given range (16.5 T, D_2O).*

T / K	δ_F / ppm		
	major	minor	difference
298	-160.8	-167.3	6.5
303	-158.3	-164.7	6.4
308	-155.7	-161.9	6.2
310	-154.7	-160.9	6.2
313	-153.4	-159.6	6.2
318	-151.1	-157.2	6.1
323	-148.8	-154.7	5.9
ppm / K	0.5	0.5	n.d.

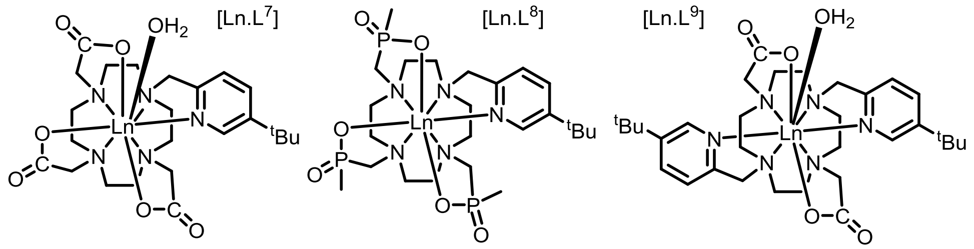

Scheme 8: *Structures of [Ln.L^{7}], [Ln.L^{8}] and [Ln.L^{9}]$^{+}$.*

SI-Table 17 : *Variation of the chemical shift of the ^{t}Bu resonance of [Dy.L$^{7-9}$] with temperature over the given range (16.5 T, D_2O).*

T / K	δ_H / ppm		
	[Dy.L^{7}]	[Dy.L^{8}]	[Dy.L^{9}]
298	-20.2	-73.1	-17.3
303	-19.6	-71.2	-16.8
308	-19.0	-69.4	-16.2
313	-18.5	-67.6	-15.7
318	-18.0	-65.9	-15.3
323	-17.5	-64.3	-14.8
ppm / K	0.1	0.4	0.1

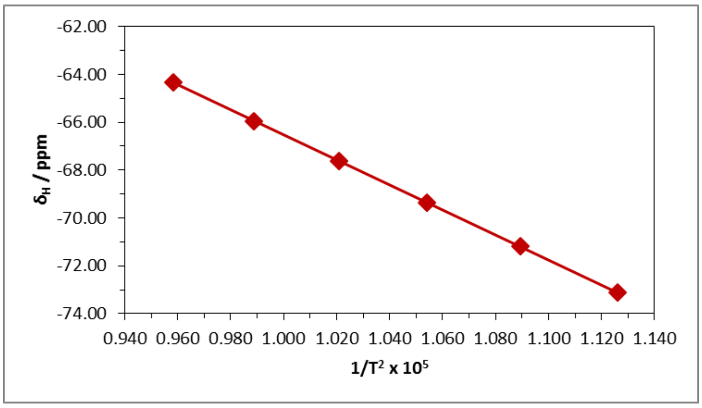

SI-Figure 22 : *Variation of the chemical shift of various resonances of [Dy.L^{8}] with 1 / T^{2} from 298to 323 K, (D_2O, 11. 7 T). A steeper gradient indicates a bigger shift in ppm / K.*

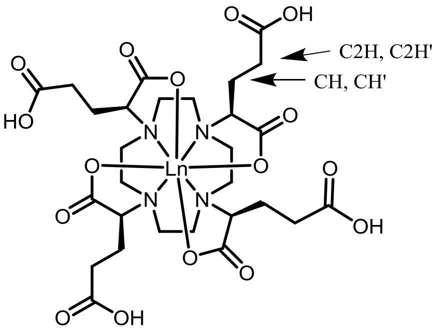

Scheme 9 : *Structure and resonances of interest of [Ln.gDOTA]$^{5-}$*

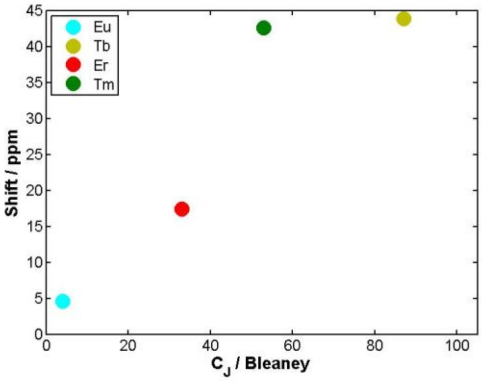

SI-Figure 23 : *Correlation of the pseudocontact shift data of the CH resonance for [Ln.gDOTA]$^{5-}$ with the Bleaney constant, C_J (295 K, 9.4 T, CD_3OD).*

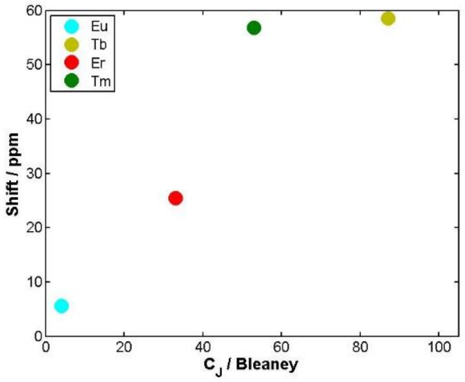

SI-Figure 24 : *Correlation of the pseudocontact shift data of the C2H resonance for [Ln.gDOTA]$^{5-}$ with the Bleaney constant, C_J (295 K, 9.4 T, CD_3OD).*

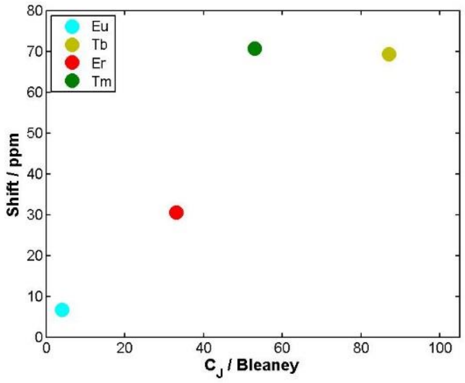

SI-Figure 25 : *Correlation of the pseudocontact shift data of the C2H' resonance for [Ln.gDOTA]$^{5-}$ with the Bleaney constant, C_J (295 K, 9.4 T, CD_3OD).*

SI-Table 18 : *Chemical shift data for the CH_3 resonances of [Ln.gDOTA]$^{5-}$ (295 K, 9.4 T, D_2O).*

	δ$_H$ / ppm							
Ln^{3+}	CH		CH'		C2H		C2H'	
	major	minor	major	minor	major	minor	major	minor
Eu	0.94	-2.6	-1.34	x	-0.5	-3.6	-2.0	-4.7
Tb	47.0	45.9	x	x	63	60.6	72.7	71.31
Er	-28.6	-15.5	-50.1	x	-29.4	-23.4	-39.3	-28.6
Tm	-68.5	-40.55	-124.9	x	-73.3	-54.8	-97.2	-68.7
Yb	-12.2	x	-38.1	x	-14.6	x	-17.3	x
Y	2.0				2.3			

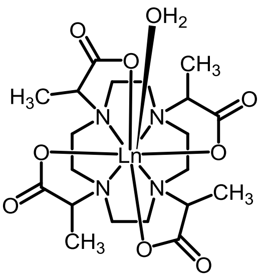

Scheme 10 : *Structure of [Ln.DOTMA]⁻ .*

SI-Table 19 : *Chemical shift data for the CH_3 resonances of $[Ln.DOTMA]^-$ (295 K, 9.4 T, D_2O). Data taken in part from [21], [Dy.DOTMA] from [22].*

Ln^{3+}	δ_H / ppm major	minor
Eu	-1.74	-3.95
Tb	61.6	67.8
Dy	81.3	74.5
Ho	35.8	42.5
Er	-43.7	-28.0
Tm	-104.0	-67.0
Yb	-14.7	*

* *not observed*

SI-Table 20 : *Variation of the chemical shift of the CH_3 resonances of $[Tm.DOTMA]^-$ with temperature (16.5 T, D_2O).*

T / K	δ_H / ppm CH_3 major[a]	CH_3 minor	ΔCH_3
298	-104.3	-65.8	38.5
303	-101.0	-64.6	36.3
308	-97.7	-63.5	34.2
313	-94.6	-62.5	32.2
318	-91.7	-61.5	30.2
323	-88.9	-60.7	28.2
ppm / K	0.6	0.2	0.4

[a]The major isomer is an 8-coordinate twisted SAP complex, whilst the minor isomer is 9-coordinate (q=1), and is a mono-capped square-antiprism.

SI-Table 21 : *Variation of the total 1-H NMR spectral width with the second order crystal field coefficient (for the Eu analogues) for complexes in axial symmetry.*

Tm			Yb		
[Ln.L^x]	Δδ / ppm	B_0^2 / cm^{-1}	[Ln.L^x]	Δδ / ppm	B_0^2 / cm^{-1}
1	70	75	1	16	75
2	112	110	2	34	110
3	150	235	3	41	228
9	380	355	5	150	700
4	454	470	4	169	470
5	681	700	9	158	355

4. Nuclear relaxation rate data for Ln complexes

(295K unless stated)

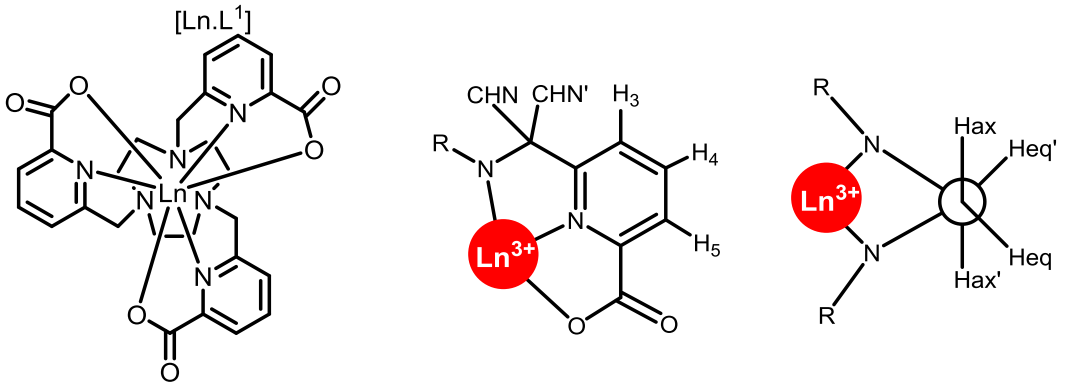

SI-Table 22 : *^{1}H nuclear relaxation rates, R_1, and single fitting values (μ_{eff} = 9.8 BM) for [Tb.L^1] (295 K, D_2O).*

^{1}H	R_1/ s^{-1} 4.7 T	9.4 T	11.7 T	14.1 T	16.5 T	Fitting values r / Å	τ_R / ps	T_{1e} / ps
pyH^4	44 ±3	85 ±5	131 ±21	152 ±7	177 ±12	6.48	196.5	0.19
pyH^3	108 ±6	195 ±5	258 ±5	316 ±9	390 ±15	5.58	124.0	0.29
Hax'	499 ±57	1027 ±55	1484 ±86	1639 ±149	1957 ±166	4.36	229.7	0.20
Heq	392 ±25	768 ±25	995 ±41	1268 ±50	1465 ±43	4.55	172.2	0.27
Hax	465 ±28	869 ±45	1155 ±59	1398 ±39	1607 ±50	4.51	195.3	0.30
pyCHN'	403 ±11	714 ±45	1002 ±21	1346 ±30	1575 ±54	4.34	107.5	0.21

SI-Table 23 : *^{1}H nuclear relaxation rates, R_1, and single fitting values (μ_{eff} = 10.3 BM) for [Dy.L^1] (295 K, D_2O).*

^{1}H	R_1/ s^{-1} 4.7 T	9.4 T	11.7 T	14.1 T	16.5 T	Fitting values r / Å	τ_R / ps	T_{1e} / ps
pyH^5	125 ±6	238 ±6	314 ±6	395 ±12	503 ±9	5.31	85.4	0.23
pyH^4	64 ±2	126 ±1	164 ±2	211 ±3	258 ±3	6.15	121.6	0.26
pyH^3	130 ±7	255 ±3	347 ±6	414 ±20	513 ±11	5.58	151.5	0.28
Heq	435 ±32	776 ±33	1252 ±37	1576 ±243	1787 ±42	4.50	156.3	0.20

SI-Table 24 : *^{1}H nuclear relaxation rates, R_1, and single fitting values (μ_{eff} = 10.4 BM) for [Ho.L^1] (295 K, D_2O).*

^{1}H	R_1/ s^{-1} 4.7 T	9.4 T	11.7 T	14.1 T	16.5 T	Fitting values r / Å	τ_R / ps	T_{1e} / ps
pyH^5	91 ±6	210 ±6	305 ±26	382 ±13	509 ±26	5.21	75.8	0.12
pyH^4	48 ±2	103 ±2	142 ±5	184 ±3	232 ±6	6.16	100.5	0.17
pyH^3	92 ±11	298 ±27	283 ±51	423 ±51	538 ±51	5.47	125.1	0.18
pyCHN	362 ±10	819 ±30	1245 ±32	1556 ±43	1777 ±60	4.55	193.4	0.12

SI-Table 25 : *^{1}H nuclear relaxation rates, R_1, and single fitting values (μ_{eff} = 9.4 BM) for [Er.L^{1}] (295 K, D_2O).*

^{1}H	R_1/ s^{-1}					Fitting values		
	4.7 T	9.4 T	11.7 T	14.1 T	16.5 T	*r* / Å	τ_R / ps	T_{1e} / ps
pyH^{3}	87 ±5	176 ±8	233 ±13	284 ±27	358 ±48	5.52	130.3	0.22
pyH^{4}	39 ±2	68 ±3	88 ±4	119 ±2	140 ±3	6.34	105.5	0.24
pyH^{5}	84 ±5	181 ±8	223 ±10	272 ±10	325 ±10	5.89	199.4	0.25
Heq	365 ±30	667 ±19	1137 ±47	1352 ±58	1666 ±196	4.40	148.1	0.17
Heq'	389 ±20	657 ±48	1085 ±51	1281 ±67	1597 ±67	4.21	107.7	0.18

SI-Table 26: *^{1}H nuclear relaxation rates, R_1, and single fitting values (μ_{eff} = 7.6 BM) for [Tm.L^{1}] (295 K, D_2O).*

^{1}H	R_1/ s^{-1}					Fitting values		
	4.7 T	9.4 T	11.7 T	14.1 T	16.5 T	*r* / Å	τ_R / ps	T_{1e} / ps
CHN'	414 ±7	989±44	1431±33	1872±321	2295±108	3.48	132.5	0.07
pyH$^{3/5}$	25 ±1	61 ±0.3	84 ±0.2	108 ±0.3	134 ±0.5	5.63	140.5	0.08
pyH^{4}	12 ±0.2	27 ±0.2	37 ±0.1	48 ±0.2	60 ±0.3	6.36	116.6	0.09
Hax'	400±19	971±51	1433±52	1745±143	2159±69	3.57	164.2	0.07
CHN	102±85	209 ±8	311 ±8	362 ±19	471 ±21	4.55	122.5	0.11
Heq	82 ±12	201 ±5	282 ±1	365 ±2	454 ±3	4.57	133.5	0.07
Heq'	94 ±1	226 ±4	321 ±2	415 ±3	515 ±3	4.47	132.0	0.07
Hax	116 ±2	277 ±5	390 ±3	510 ±3	636 ±9	4.29	121.5	0.07

SI-Table 27 *^{1}H nuclear relaxation rates, R_1, and single fitting values (μ_{eff} = 4.5 BM) for [Yb.L^{1}] (295 K, D_2O).*

^{1}H	R_1/ s^{-1}					Fitting values		
	4.7 T	9.4 T	11.7 T	14.1 T	16.5 T	*r* / Å	τ_R / ps	T_{1e} / ps
pyCHN'	89 ±1	159 ±2	210 ±3	257 ±4	311 ±4	3.41	134.7	0.06
pyH^{3}	5.7 ±0.2	9.5 ±0.5	12.5 ±0.1	12.8 ±0.7	18.6 ±0.2	5.46	112.4	0.07
pyH^{4}	3.1 ±0.1	4.8 ±0.2	5.8 ±0.1	7.4 ±0.4	8.4 ±0.1	6.29	128.7	0.10
pyH^{5}	5.4 ±0.1	9.4 ±0.4	12.0 ±0.1	15.4 ±0.7	17.8 ±0.1	5.45	13.5	0.06
CHN	27 ±3	34 ±2	46 ±1	61 ±1	54 ±1	4.42	99.9	0.11
Heq	21 ±1	32 ±1	42 ±1	52 ±1	62 ±1	4.34	92.5	0.07
Heq'	23 ±1	36 ±1	48 ±1	59 ±1	70 ±1	4.33	112.1	0.08
Hax	29 ±1	44 ±1	59 ±1	73 ±1	88 ±1	4.01	79.4	0.06

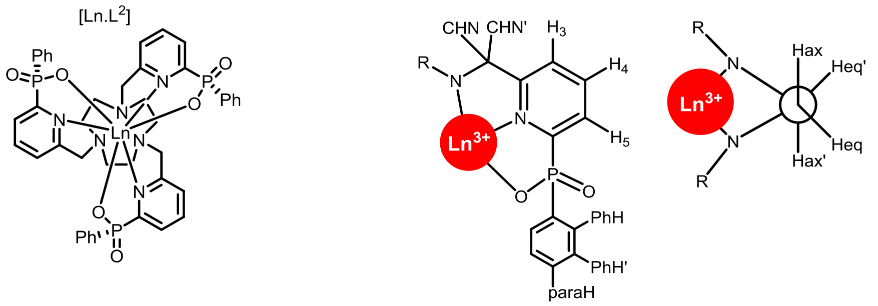

SI-Table 28 : *^{1}H nuclear relaxation rates, R_1, and single fitting values (μ_{eff} = 9.8 BM) for [Tb.L^2] (295 K, CD_3OD).*

^{1}H	4.7 T	9.4 T	R$_1$/ s^{-1} 11.7 T	14.1 T	16.5 T	Fitting values r / Å	τ_R / ps	T_{1e} / ps
Hax'	571 ±18	1178 ±12	1528 ±14	1834 ±22	2208 ±24	4.27	86.7	0.26
Hax	526 ±7	1090 ±11	1357 ±5	1687 ±15	2057 ±26	4.30	160.3	0.27
CHN	384 ±11	809 ±12	1022 ±6	1298 ±26	1550 ±26	4.50	164.8	0.24
PhH'	34 ±1	69 ±1	89 ±1	107 ±1	127 ±1	6.88	194.8	0.27
PhH	14 ±0.1	27 ±1	35 ±1	41 ±1	49 ±1	8.08	196.5	0.31
pyH^5	89 ±1	181 ±1	234 ±1	287 ±3	330 ±6	5.87	206.6	0.25
pyH^4	43 ±1	87 ±1	109 ±1	133 ±1	153 ±1	6.68	214.9	0.28
pyH^3	97 ±2	199 ±2	252 ±2	305 ±3	351 ±6	5.81	220.63	0.26
Heq	388 ±6	806 ±13	1031 ±12	1143 ±31	1471 ±27	4.59	189.6	0.29
CHN'	1877 ±63	3253 ±125	4572 ±82	5848 ±230	6784 ±183	3.52	159.2	0.29

SI-Table 29 : *^{1}H nuclear relaxation rates, R_1, and single fitting values (μ_{eff} = 10.3 BM) for [Dy.L^2] (295 K, CD_3OD).*

^{1}H	4.7 T	9.4 T	R$_1$/ s^{-1} 11.7 T	14.1 T	16.5 T	Fitting values r / Å	τ_R / ps	T_{1e} / ps
Hax'	787 ±25	1608 ±16	2058 ±7	2546 ±20	2895 ±71	4.22	212.8	0.28
Hax	710 ±27	1484 ±9	1848 ±5	2312 ±13	2593 ±45	4.30	225.6	0.27
PhH'	46 ±1	93 ±1	131 ±4	149 ±2	182 ±2	6.69	19.5	0.26
PhH	20 ±1	37 ±1	52 ±1	59 ±1	72 ±1	7.81	181.0	0.32
pyH^5	136 ±2	261 ±5	310 ±16	414 ±13	459 ±12	5.73	187.0	0.36
pyH^3	150 ±2	282 ±5	344 ±7	455 ±7	518 ±11	5.59	213.6	0.35

SI-Table 30 : *^{1}H nuclear relaxation rates, R_1, and single fitting values (μ_{eff} = 10.4 BM) for [Ho.L^2] (295 K, CD_3OD).*

^{1}H	4.7 T	9.4 T	R$_1$/ s^{-1} 11.7 T	14.1 T	16.5 T	Fitting values r / Å	τ_R / ps	T_{1e} / ps
PhH	25 ±3	55 ±1	68 ±1	84 ±2	98 ±2	7.46	206.8	0.26
pyH^5	94 ±4	199 ±10	278 ±19	394 ±25	482 ±25	5.21	72.4	0.12
pyH^4	44 ±2	102 ±4	141 ±5	170 ±4	203 ±4	6.58	199.5	0.17
pyH^3	92 ±4	218 ±13	299 ±8	382 ±8	433 ±16	5.78	207.0	0.14
Heq'	458 ±7	1076 ±26	1367 ±37	1658 ±36	2166 ±38	4.38	135.9	0.23

SI-Table 31 : *^{1}H nuclear relaxation rates, R_1, and single fitting values (μ_{eff} = 9.4 BM) for [Er.L^2] (295 K, CD_3OD).*

^{1}H	R_1/ s^{-1}					Fitting values		
	4.7 T	9.4 T	11.7 T	14.1 T	16.5 T	r / Å	τ_R / ps	T_{1e} / ps
CHN’	1893 ±32	3562 ±104	4391 ±44	5408 ±194	6592 ±271	3.43	181.6	0.24
pyH^5	98 ±1	182 ±2	233 ±4	280 ±3	327 ±6	5.73	192.0	0.31
pyH^3	107 ±2	192 ±2	245 ±3	295 ±3	343 ±6	5.69	188.3	0.33
pyH^4	49 ±1	88 ±1	108 ±1	131 ±1	151 ±1	6.53	198.2	0.36
PhH’	33 ±1	58 ±1	74 ±1	90 ±1	104 ±1	6.93	182.8	0.35
Hax	485 ±11	917 ±11	1184 34±	1427 ±39	1662 ±64	4.37	194.1	0.29
Heq	381 ±9	731 ±12	938 ±31	1120 ±33	1319 ±52	4.54	195.3	0.29
CHN	434 ±12	802 ±18	1048 ±30	1252 ±29	1473 ±49	4.45	185.5	0.30
Hax’	553 ±14	1033 ±16	1314 ±31	1586 ±26	1832 ±13	4.30	200.0	0.31

SI-Table 32 : *^{1}H nuclear relaxation rates, R_1, and single fitting values (μ_{eff} = 7.6 BM) for [Tm.L^2] (295 K, CD_3OD).*

^{1}H	R_1/ s^{-1}					Fitting values		
	4.7 T	9.4 T	11.7 T	14.1 T	16.5 T	r / Å	τ_R / ps	T_{1e} / ps
CHN’	574±10	1304±35	1700±26	2133±16	2544±88	3.51	182.0	0.11
pyH^3	30±1	69±0.3	92±1	116±1	139±1	5.68	174.1	0.10
pyH^5	28±1	65±0.4	86±1	109±1	130±0.4	5.75	177.2	0.10
pyH^4	13±0.4	31±0.2	40±0.2	50±0.2	56±0.1	6.61	243.9	0.08
Hax’	517±18	1390±53	1550±26	1806±91	2459±25	3.56	178.6	0.14
CHN	120±1	287±3	398±2	495±1	558±13	4.49	227.2	0.06
Heq	104±1	244±2	333±1	415±1	475±12	4.63	215.1	0.08
Heq’	114±1	261±3	360±2	442±1	504±14	4.59	221.6	0.08
Hax	133±2	323±3	430±3	542±1	631±6	4.42	205.0	0.08

SI-Table 33 : *^{1}H nuclear relaxation rates, R_1, and single fitting values (μ_{eff} = 4.5 BM) for [Yb.L^2] (295 K, CD_3OD).*

^{1}H	R_1/ s^{-1}					Fitting values		
	4.7 T	9.4 T	11.7 T	14.1 T	16.5 T	r / Å	τ_R / ps	T_{1e} / ps
CHN’	104 ±1	193 ±1	243 ±3	283 ±1	326 ±1	3.45	228.7	0.07
pyH$^{3/5}$	6.2 ±0.1	10.5 ±0.5	12.7 ±0.1	15.0 ±0.1	17.1 ±0.1	5.68	214.3	0.09
pyH^4	4.3 ±0.1	5.7 0.2±	6.5 ±0.1	7.4 ±0.1	8.3 ±0.1	6.58	193.2	0.18
CHN	23 ±2	43 ±1	54 ±1	66 ±1	79 ±1	4.34	161.4	0.07
Heq	23 ±2	37 ±1	46 ±1	55 ±1	63 ±1	4.57	174.1	0.10
Heq’	27 ±1	42 ±1	52 ±1	62 ±1	70 ±1	4.51	178.4	0.11
HAx	33 ±1	51 ±1	64 ±1	75 ±1	87 ±1	4.34	162.2	0.11

SI-Table 34 *^{31}P relaxation rates at a range of temperature K for [Tm.L^2], (CD_3OD, 16.5 T, 1 mM).*

R_1 / s^{-1}	T	1 / T^2 x 10^{-3}
463	295	0.0115
446	297	0.0114
426	299	0.0112
413	301	0.0111
397	302	0.0109

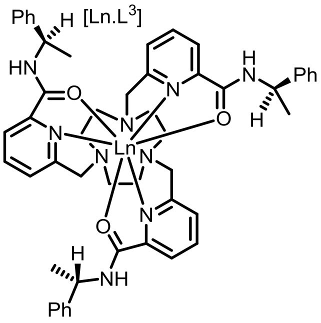

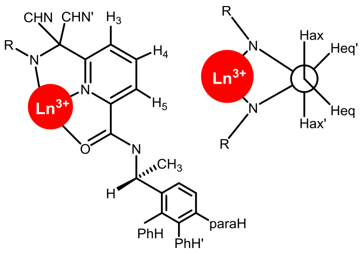

SI-Table 35 : *^{1}H nuclear relaxation rates, R_1, and single fitting values (μ_{eff} = 9.8 BM) for $[Tb.L^3]^{3+}$ (295 K, CD_3OD).*

^{1}H	R$_1$/ s^{-1} 4.7 T	9.4 T	11.7 T	14.1 T	16.5 T	Fitting values r / Å	τ_R / ps	T_{1e} / ps
Hax'	793 ±45	1471 ±41	1828 ±25	2230 ±31	2574 ±58	4.18	198.1	0.35
Heq	336 ±7	668 ±28	880 ±20	999 ±36	1204 ±33	4.75	214.2	0.28
CH_3	53 ±1	96 ±1	122 ±1	147 ±1	170 ±1	6.57	196.2	0.36
H	95 ±2	169 ±2	210 ±1	262 ±3	305 ±4	5.93	169.2	0.37
PhH	23 ±1	40 ±1	50 ±1	59 ±1	67 ±2	7.70	221.6	0.41
pyH^4	46 ±3	93 ±1	122 ±1	148 ±2	168 ±3	6.56	220.0	0.24
pyH^5	115 ±2	216 ±6	294 ±4	361 ±6	400 ±16	5.67	210.4	0.27
pyH^3	122 ±3	226 ±4	305 ±7	365 ±8	433 ±10	5.59	174.4	0.30

SI-Table 36: *^{1}H nuclear relaxation rates, R_1, and single fitting values (μ_{eff} = 10.3 BM) for $[Dy.L^3]^{3+}$ (295 K, CD_3OD).*

^{1}H	R$_1$/ s^{-1} 4.7 T	9.4 T	11.7 T	14.1 T	16.5 T	Fitting values r / Å	τ_R / ps	T_{1e} / ps
PhH	23 ±1	40 ±3	49 ±1	61 ±2	69 ±3	7.89	195.5	0.45
PhH'	29 ±1	53 ±2	70 ±1	84 ±1	99 ±2	7.40	180.5	0.36
pyH^4	59 ±3	114 ±5	141 ±6	176 ±5	212 ±5	6.49	158.2	0.35
pyH^3	94 ±3	187 ±5	249 ±15	315 ±10	385 ±22	5.79	133.5	0.26

SI-Table 37 : *^{1}H nuclear relaxation rates, R_1, and single fitting values (μ_{eff} = 10.4 BM) for $[Ho.L^3]^{3+}$ (295 K, CD_3OD).*

^{1}H	R$_1$/ s^{-1} 4.7 T	9.4 T	11.7 T	14.1 T	16.5 T	Fitting values r / Å	τ_R / ps	T_{1e} / ps
Hax'	721 ±51	1544 ±24	2299 ±123	2494 ±116	3252 ±93	4.15	173.6	0.21
Heq	328 ±7	830 ±37	1026 ±19	1216 ±31	1647 ±99	4.59	135.2	0.22
Heq'	448 ±13	1044 ±47	1359 ±30	1531 ±66	1864 ±150	4.56	248.5	0.18
CH_3	50 ±1	113 ±2	145 ±1	178 ±2	226 ±10	6.41	145.5	0.24
H	81 ±1	184 ±2	240 ±4	296 ±2	362 ±19	5.97	163.6	0.24
PhH'	20 ±1	47 ±1	63 ±1	72 ±1	95 ±6	7.45	159.8	0.22
pyH^6	112 ±4	262 ±10	378 ±6	405 ±9	475 ±46	5.67	238.5	0.15

SI-Table 38: *^{1}H nuclear relaxation rates, R_1, and single fitting values (μ_{eff} = 9.4 BM) for $[Er.L^3]^{3+}$ (295 K, CD_3OD).*

^{1}H	R_1/ s^{-1} 4.7 T	9.4 T	11.7 T	14.1 T	16.5 T	Fitting values r / Å	τ_R / ps	T_{1e} / ps
pyH^4	36 ±2	61 ±3	93 ±7	108 ±7	122 ±7	6.65	144.1	0.29
PhH	18 ±1	30 ±1	40 ±1	46 ±1	54 ±1	7.75	189.8	0.38
CH_3	52 ±1	85 ±1	107 ±1	132 ±2	153 ±2	6.47	160.2	0.40

SI-Table 39 : *^{1}H nuclear relaxation rates, R_1, and single fitting values (μ_{eff} = 7.6 BM) for $[Tm.L^3]^{3+}$ (295 K, CD_3OD).*

^{1}H	R_1/ s^{-1} 4.7 T	9.4 T	11.7 T	14.1 T	16.5 T	Fitting values r / Å	τ_R / ps	T_{1e} / ps
CHN’	566±20	1413±97	1718±20	2088±43	2482±76	3.53	239.3	0.09
pyH^3	31±0.3	75±2	105±1	125±2	149±2	5.62	213.9	0.07
pyH^4	18±0.4	40±2	53±1	63±1	73±1	6.36	234.4	0.11
PhH’	6±0.1	13±0.1	16±0.1	20±0.1	24±0.2	7.68	179.4	0.15
PhH	4±0.1	8±0.1	10±0.1	12±0.2	15±0.2	8.27	150.7	0.19
H	22±0.1	50±0.4	65±0.3	82±1	97±1	6.71	186.5	0.15
CH_3	14±0.1	29±0.1	37±0.1	46±0.2	54±0.1	6.05	185.6	0.11
CHN	80±1	202±6	256±3	314±4	363±3	4.84	246.4	0.07
Heq	118±3	283±5	366±4	443±4	512±8	4.58	242.7	0.08
Heq’	130±24	327±10	411±9	501±6	580±9	4.48	251.8	0.07
Hax	158±2	377±9	479±5	589±4	699±5	4.36	212.6	0.10

SI-Table 40 : *^{1}H nuclear relaxation rates, R_1, and single fitting values (μ_{eff} = 4.5 BM) for $[Yb.L^3]^{3+}$ (295 K, CD_3OD).*

^{1}H	R_1/ s^{-1} 4.7 T	9.4 T	11.7 T	14.1 T	16.5 T	Fitting values r / Å	τ_R / ps	T_{1e} / ps
pyH^3	7.4 ±1	12.0 ±1	15.3 ±1	17.4 ±1	20.6 ±1	5.47	160.6	0.09
pyH^5	7.9 ±1	12.3 ±1	15.8 ±1	17.8 ±1	21.2 ±1	5.51	170.8	0.11
pyH^4	5.0 ±1	7.3 ±1	8.6 ±1	10.1 ±1	10.7 ±1	6.23	211.2	0.15
CHN	19 ±1	29 ±1	39 ±1	46 ±1	53 ±1	4.67	156.0	0.10
Heq	30 ±1	43 ±1	56 ±1	65 ±1	74 ±1	4.46	157.2	0.12
Heq’	34 ±1	48 ±1	63 ±1	73 ±1	83 ±1	4.38	153.7	0.12
Hax	43 ±1	62 ±1	78 ±1	89 ±1	102 ±1	4.26	166.3	0.14

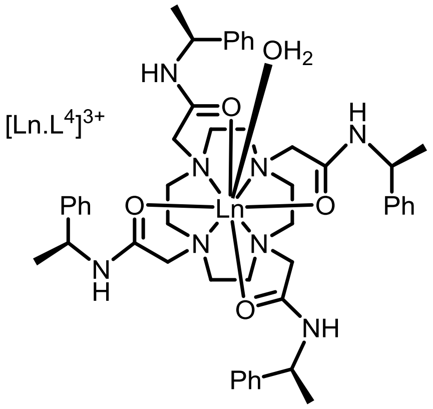

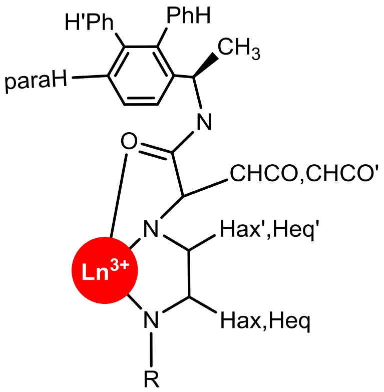

SI-Table 41 : *^{1}H nuclear relaxation rates,R_1, and single fitting values (μ_{eff} = 9.8 BM) for [Tb.L^4]$^{3+}$ (295 K, D_2O).*

^{1}H	R$_1$/ s^{-1} 4.7 T	9.4 T	11.7 T	14.1 T	16.5 T	Fitting values r / Å	τ_R / ps	T_{1e} / ps
Hax'	3020 ±82	379 ±172	5527 ±41	6050 ±168	6693 ±398	3.58	268.9	0.63
CHCO'	2699 ±83	4088 ±339	4588 ±23	4940 ±116	5263 ±186	3.66	289.4	0.55
paraH	48 ±1	75 ±1	85 ±1	92 ±1	101 ±2	7.13	350.3	0.55
PhH'	76 ±1	121 ±1	135 ±1	148 ±1	161 ±2	6.58	358.4	0.52
PhH	224 ±1	363 ±1	407 ±1	429 ±3	477 ±15	5.47	386.6	0.46
CH$_3$	66 ±1	101 ±1	114 ±1	125 ±1	131 ±1	6.77	382.2	0.53
Heq'	708 ±12	1111 ±7	1275 ±5	1405 ±6	1554 ±33	4.55	316.9	0.56
Hax	1453 ±12	2205 ±145	2840 ±8	3136 ±23	3420 ±58	3.99	272.3	0.53

SI-Table 42 *^{1}H nuclear relaxation rates,R_1, and single fitting values (μ_{eff} = 10.3 BM) for [Dy.L^4]$^{3+}$ (295 K, D_2O).*

^{1}H	R$_1$/ s^{-1} 4.7 T	9.4 T	11.7 T	14.1 T	16.5 T	Fitting values r / Å	τ_R / ps	T_{1e} / ps
Hax'	3117 ±98	6114 ±128	6916 ±171	7722 ±268	9381 ±827	3.50	266.7	0.41
paraH	53 ±1	92 ±1	103 ±1	115 ±1	125 ±1	7.07	360.2	0.43
PhH'	83 ±1	146 ±1	166 ±1	184 1±	198 ±2	6.51	376.0	0.38
CH$_3$	76 ±1	126 ±1	144 ±1	160 2±	171 ±2	6.70	360.1	0.47
Heq'	785 ±11	1374 ±10	1600 ±8	1799 17±	2016 ±85	4.49	301.8	0.46
Heq	762 ±10	1379 ±13	1602 ±12	1784 ±20	1981 ±66	4.48	329.5	0.39
Hax	1769 ±52	3038 ±71	3633 ±47	3959 ±51	4448 ±57	3.93	314.6	0.46

SI-Table 43 *^{1}H nuclear relaxation rates,R_1, and single fitting values (μ_{eff} = 10.4 BM) for [Ho.L^4]$^{3+}$ (295 K, D_2O).*

^{1}H	R$_1$/ s^{-1} 4.7 T	9.4 T	11.7 T	14.1 T	16.5 T	Fitting values r / Å	τ_R / ps	T_{1e} / ps
CHCO'	1955 ±321	3715 ±121	4590 ±53	5214 ±241	6231 ±319	3.76	230.2	0.39
paraH	37 ±1	71 ±1	72 ±1	93 ±1	98 ±1	7.45	307.0	0.43
PhH'	189 ±8	356 ±3	388 ±3	443 ±3	461 ±6	6.85	305.4	0.41
CH$_3$	52 ±1	97 ±1	115 ±1	130 ±1	137 ±1	6.94	369.2	0.29
Heq'	613 ±9	1185 ±12	1406 ±11	1595 ±12	1707 ±46	4.57	358.3	0.27
Heq	612 ±9	1185 ±6	1410 ±14	1590 ±12	1708 ±47	4.57	360.6	0.26

SI-Table 44 *^{1}H nuclear relaxation rates, R_1, and single fitting values (μ_{eff} = 9.4 BM) for $[Er.L^4]^{3+}$ (295 K, D_2O).*

^{1}H	R$_1$/ s^{-1}					Fitting values		
	4.7 T	9.4 T	11.7 T	14.1 T	16.5 T	r / Å	τ_R / ps	T_{1e} / ps
PhH	33 ±1	60 ±3	77 ±1	88 ±1	97 ±2	7.01	270.8	0.30
paraH	20 ±1	35 ±2	45 ±1	51 ±1	57 ±1	7.68	256.9	0.34
CH_3	30 ±1	56 ±1	69 ±1	78 ±1	85 ±1	7.12	312.3	0.27

SI-Table 45 *^{1}H nuclear relaxation rates, R_1, and single fitting values (μ_{eff} = 7.6 BM) for $[Tm.L^4]^{3+}$ (295 K, D_2O).*

^{1}H	R$_1$/ s^{-1}					Fitting values		
	4.7 T	9.4 T	11.7 T	14.1 T	16.5 T	r / Å	τ_R / ps	T_{1e} / ps
Heq’	253 ±1	468 ±8	521 ±1	584 ±3	617 ±2	4.35	439.0	0.14
Heq	248 ±1	453 ±7	506 ±1	566 ±3	601 ±1	4.39	421.5	0.16
CH_3	18 ±1	28 ±1	33 ±1	36 ±1	38 ±1	7.14	341.1	0.33
CHCO	201 ±1	364 ±2	421 ±1	472 ±1	518 ±7	4.60	334.1	0.22
CHCO’	597 ±12	1052 ±14	1186 ±1	1354 ±4	1426 ±6	3.86	365.9	0.22
Hax	536 ±2	999 ±15	1187 ±7	1316 ±2	1412 ±7	3.84	376.6	0.16

SI-Table 46 : *^{1}H nuclear relaxation rates, R_1, and single fitting values (μ_{eff} = 4.5 BM) for $[Yb.L^4]^{3+}$ (295 K, D_2O).*

^{1}H	R$_1$/ s^{-1}					Fitting values		
	4.7 T	9.4 T	11.7 T	14.1 T	16.5 T	r / Å	τ_R / ps	T_{1e} / ps
Hax’	163 ±1	242 ±1	271 ±1	298 ±1	312 ±1	3.53	353.6	0.15
Heq’	51 ±1	69 ±1	77 ±1	84 ±1	87 ±1	4.45	312.1	0.21
Heq	50 ±1	67 ±1	75 ±1	82 ±1	85 ±1	4.48	299.3	0.22
PhH	30 ±1	43 ±1	49 ±1	53 ±1	55 ±1	4.72	356.6	0.16
PhH’	11 ±1	15 ±1	18 ±1	19 ±1	20 ±1	5.65	310.2	0.18
CHCO	41 ±1	56 ±1	62 ±1	68 ±1	69 ±1	4.59	346.5	0.20
CHCO’	106 ±1	157 ±1	173 ±1	190 ±1	196 ±1	3.78	393.8	0.14
Hax	98 ±1	148 ±1	169 ±1	186 ±1	192 ±1	3.79	372.8	0.13

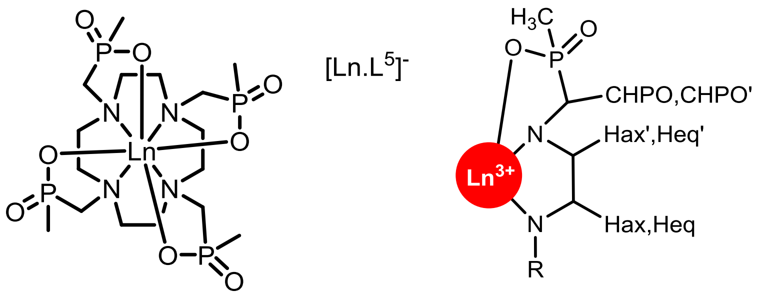

SI-Table 47 : *^{1}H nuclear relaxation rates, R_1, and single fitting values (μ_{eff} = 9.8 BM) for $[Tb.L^5]^-$ (295 K, D_2O).*

^{1}H	R$_1$/ s^{-1}					Fitting values		
	4.7 T	9.4 T	11.7 T	14.1 T	16.5 T	r / Å	τ_R / ps	T_{1e} / ps
CHPO	609 ±9	1296 ±22	1577 ±20	1833 ±15	2205 ±55	4.29	230.4	0.27
CH_3	413 ±5	791 ±5	991 ±4	1156 ±15	1304 ±14	4.68	253.5	0.31
Heq	496 ±8	992 ±19	1210 ±12	1431 ±21	1620 ±37	4.51	256.2	0.29
Heq’	491 ±6	977 ±19	1171 ±12	1366 ±12	1609 ±38	4.53	236.5	0.32

SI-Table 48: *^1H nuclear relaxation rates, R_1, and single fitting values (μ_{eff} = 10.3 BM) for $[Dy.L^5]^-$ (295 K, D_2O).*

^1H	R₁/ s⁻¹					Fitting values		
	4.7 T	9.4 T	11.7 T	14.1 T	16.5 T	*r* / Å	τ_R / ps	T_{1e} / ps
CHPO	1021 ±42	1907 ±34	2158 ±53	2551 ±63	2903 ±99	4.24	265.3	0.44
CH_3	639 ±17	116 ±12	1377 ±8	1658 ±35	1797 ±43	4.58	249.8	0.43
Heq	736 ±23	1364 ±56	1734 ±33	1976 ±43	2375 ±190	4.39	210.8	0.39
Heq’	707 ±23	1359 ±30	1638 ±28	1950 ±48	2388 ±152	4.37	182.1	0.40

SI-Table 49: *^1H nuclear relaxation rates, R_1, and single fitting values (μ_{eff} = 10.4 BM) for $[Ho.L^5]^-$ (295 K, D_2O).*

^1H	R₁/ s⁻¹					Fitting values		
	4.7 T	9.4 T	11.7 T	14.1 T	16.5 T	*r* / Å	τ_R / ps	T_{1e} / ps
CH_3	519 ±18	921 ±17	1124 ±21	1446 ±62	1685 ±52	4.62	154.8	0.41
Heq	606 ±28	1176 ±25	1503 ±27	1820 ±88	2108 ±88	4.48	204.4	0.33
Heq’	598 ±34	1203 ±27	1533 ±33	1841 ±74	2098 ±78	4.49	230.4	0.30

SI-Table 50: *^1H nuclear relaxation rates, R_1, and single fitting values (μ_{eff} = 9.4 BM) for $[Er.L^5]^-$ (295 K, D_2O).*

^1H	R₁/ s⁻¹					Fitting values		
	4.7 T	9.4 T	11.7 T	14.1 T	16.5 T	*r* / Å	τ_R / ps	T_{1e} / ps
Heq’	720 ±21	1080 ±7	1303 ±11	1519 ±6	1733 ±34	4.38	210.3	0.58
Heq	700 ±26	1051 ±13	1295 ±20	1546 ±9	1735 ±52	4.36	199.7	0.54
CH_3	488 ±17	694 ±7	850 ±3	985 ±3	1106 ±5	4.72	215.7	0.64
CHPO	581 ±14	942 ±13	1130 9±	1290 ±6	1474 ±20	4.49	239.1	0.49

SI-Table 51 : *^1H nuclear relaxation rates, R_1, and single fitting values (μ_{eff} = 7.6 BM) for $[Tm.L^5]^-$ (295 K, D_2O).*

^1H	R₁/ s⁻¹					Fitting values		
	4.7 T	9.4 T	11.7 T	14.1 T	16.5 T	*r* / Å	τ_R / ps	T_{1e} / ps
Heq’	751±38	1143±12	1260±15	1388±10	1483±25	3.90	344.4	0.39
Heq	739±21	1116±16	1248±19	1365±12	1450±32	3.91	349.4	0.39
CH_3	446±40	665±2	745±3	831±5	894±5	4.27	302.3	0.43
CHPO	394±9	632±6	732±7	808±3	900±6	4.26	288.8	0.35

SI-Table 52: *^1H nuclear relaxation rates, R_1, and single fitting values (μ_{eff} = 4.5 BM) for $[Yb.L^5]^-$ (295 K, D_2O).*

^1H	R₁/ s⁻¹					Fitting values		
	4.7 T	9.4 T	11.7 T	14.1 T	16.5 T	*r* / Å	τ_R / ps	T_{1e} / ps
CHPO’	202 ±2	249 ±1	278 ±1	311 ±1	339 ±2	3.58	162.5	0.25
Heq’	59 ±1	73 ±1	83 ±1	91 ±1	98 ±1	4.42	196.4	0.26
Heq	57 ±1	72 ±1	82 ±	90 ±1	97 ±1	4.43	206.3	0.25
CH_3	37 ±1	47 ±1	53 ±1	58 ±1	63 ±1	4.76	204.8	0.25
CHPO	41 ±1	55 ±1	62 ±1	69 ±1	75 ±1	4.60	206.1	0.22
Hax	127 ±1	174 ±1	198 ±1	219 ±1	240 ±1	3.78	226.2	0.21
Hax’	136 ±1	190 ±1	217 ±1	245 ±1	271 ±1	3.69	205.8	0.19

[Ln.L[6]]

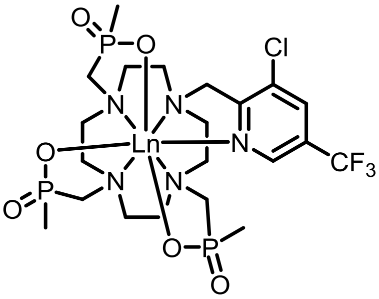

SI-Table 53: *^{19}F nuclear relaxation rates of the CF_3 resonance for [Ln.L[6]] used in global fitting (295 K, D_2O)*

Ln^{3+}	δ_F / ppm	R_1/ s^{-1} 4.7 T	9.4 T	11.7 T	14.1 T	16.5 T
Tb	-158.4	56±1	89±1	117±3	133±1	150±4
Dy	-162.4	64±2	114±1	142±1	166±1	192±1
Ho	-107.8	67±1	129±1	154±2	189±1	218±1
Er	-16.9	94±1	136±1	175±1	188±1	219±3
Tm	17.1	59±1	107±1	132±169	152±1	173±1
Yb	-41.5	14.2±0.5	15.1±0.1	17.3±1.1	18.0±0.1	19.4±0.3

SI-Table 54: *^{19}F nuclear relaxation rates of the CF_3 resonance of the carboxylate analogue, [Ln.L[10]], used in single fitting (295 K, D_2O)*

Ln^{3+}	R_1 / s^{-1}					Fitting values		
	4.7 T	9.4 T	11.7 T	14.1 T	16.5 T	r / Å	τ_R / ps	T_{1e} / ps
Dy	70 ±1	132 ±1	158 ±2	197 ±1	233 ±2	6.36	189.9	0.42
Tm	27 ±1	54 ±2	71 ±1	90 ±2	113 ±3	5.65	112.1	0.14

[Ln.L[7]]

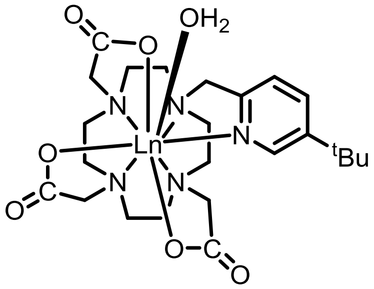

SI-Table 55 : *^{1}H nuclear relaxation rates of the ^{t}Bu resonance for [Ln.L[7]] used in global fitting (295 K, D_2O).*

Ln^{3+}	δ_H / ppm	R_1/ s^{-1} 4.7 T	9.4 T	11.7 T	14.1 T	16.5 T	R_2 / s^{-1} 4.7 T	9.4 T
Tb	-11.6	72±1	104±2	128±1	146±1	169±1	109	79
Dy	-20.5	73±1	124±1	149±1	170±1	210±2	107	176
Ho	-7.4	45±1	89±1	118±1	144±1	169±1	50	223
Er	7.0	29±1	59±1	80±1	97±1	120±1	81	108
Tm	10.8	31±1	54±1	64±1	71±1	82±1	66	94
Yb	6.3	6.4±0.1	7.6±0.3	8.6±0.1	9.5±0.1	10.7±0.1	59	65

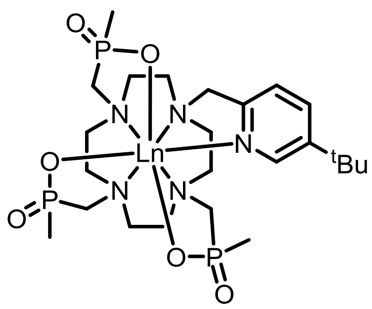

SI-Table 56: *^{1}H nuclear relaxation rates of the ^{t}Bu resonance for [Ln.L^8] used in global fitting (295 K, D_2O).*

Ln^{3+}	δ_H / ppm	R_1/ s^{-1}					R_2 / s^{-1}	
		4.7 T	9.4 T	11.7 T	14.1 T	16.5 T	4.7 T	9.4 T
Tb	-75.9	53±2	81±2	91±1	102±1	113±1	101	224
Dy	-75.0	59±1	96±1	114±1	132±1	150±1	71	205
Ho	-31.8	61±2	100±1	127±1	147±1	166±1	81	217
Er	38.2	88±2	120±2	135±1	152±1	169±1	134	341
Tm	67.0	55±1	97±1	115±1	129±1	142±1	76	202
Yb	16.3	10.7±0.1	11.4±0.7	11.6±0.6	12.0±0.1	12.4±0.3	25	46

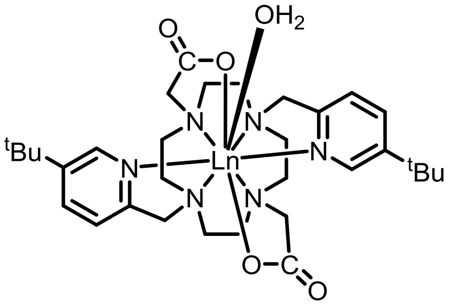

SI-Table 57: *^{1}H nuclear relaxation rates of the ^{t}Bu resonance for [Ln.L^9]$^+$ used in global fitting (295 K, D_2O).*

Ln^{3+}	δ_H / ppm	R_1/ s^{-1}					R_2 / s^{-1}	
		4.7 T	9.4 T	11.7 T	14.1 T	16.5 T	4.7 T	9.4 T
Tb	-7.2	103±1	151±1	169±1	188±1	207±2	128	192
Dy	-17.8	119±1	174±1	203±1	230±1	256±1	138	223
Ho	-7.0	56±1	102±2	138±2	156±1	177±4	70	156
Er	3.4	19±1	41±5	53±7	71±9	81±12	54	102
Tm	6.2	21±1	40±1	51±1	59±1	68±1	48	86
Yb	9.1	4.7±0.2	7.1±0.1	8.3±0.2	9.6±0.3	10.5±0.2	92	97

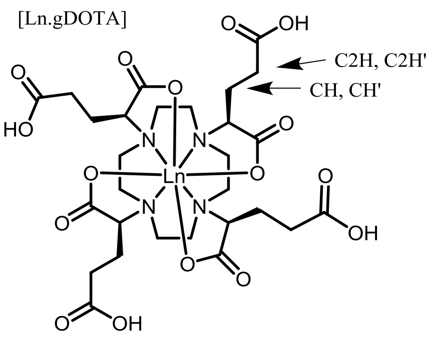

SI-Table 58: *^{1}H nuclear relaxation rates,R_1, and single fitting values (μ_{eff} = 9.4 BM) for [Er.gDOTA]$^{5-}$ (295 K, D_2O).*

$^1H^+$	R_1/ s^{-1}					Fitting values		
	4.7 T	9.4 T	11.7 T	14.1 T	16.5 T	*r* / Å	τ_R / ps	T_{1e} / ps
C2H	76 ±2	121 ±3	140 ±2	155 ±1	174 ±4	6.40	292.4	0.52
C2H'	116 ±4	188 ±3	229 ±9	251 ±3	269 ±5	5.89	326.4	0.41
CH	198 ±5	352 ±13	393 ±16	468 ±7	539 ±8	5.31	213.2	0.46

SI-Table 59: *^{1}H nuclear relaxation rates,R_1, and single fitting values (μ_{eff} = 7.6 BM) for [Tm.gDOTA]$^{5-}$ (295 K, D_2O).*

$^1H^+$	R_1/ s^{-1}					Fitting values		
	4.7 T	9.4 T	11.7 T	14.1 T	16.5 T	*r* / Å	τ_R / ps	T_{1e} / ps
CH'	288 ±20	432 ±15	487 ±16	533 ±4	560 ±24	4.56	352.4	0.38
C2H'	54 ±2	85 ±1	94 ±3	105 ±1	1131 ±	5.99	327.1	0.36
C2H	81 ±2	133 ±24	158 ±1	168 ±1	184 ±2	5.48	349.0	0.28
CH	189 ±3	298 ±4	333 ±4	364 ±3	382 ±4	4.85	349.8	0.34

SI-Table 60 : *^{1}H nuclear relaxation rates,R_1, and single fitting values (μ_{eff} = 4.5 BM) for [Yb.gDOTA]$^{5-}$ (295 K, D_2O).*

$^1H^+$	R_1/ s^{-1}					Fitting values		
	4.7 T	9.4 T	11.7 T	14.1 T	16.5 T	*r* / Å	τ_R / ps	T_{1e} / ps
Heq'	84 ±10	98 ±4	106 ±3	109 ±1	114 ±3	4.38	241.5	0.38
Heq	82 ±10	95 ±3	102 ±3	108 ±1	112 ±3	4.37	254.2	0.35

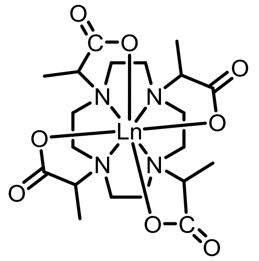

SI-Table 61 : *^{1}H nuclear relaxation rates,R_1, and single fitting values (μ_{eff} = 7.6) for [Tm.DOTMA]$^{-}$ (295 K, D_2O).*

^1H	R_1 / s^{-1}					Fitting values		
	4.7 T	9.4 T	11.7 T	14.1 T	16.5 T	*r* / Å	τ_R / ps	T_{1e} / ps
CH_3 major	143 ±2	243 ±3	275 ±2	306 ±1	342 ±1	4.98	305	0.30
CH_3 minor	114 ±4	191 ±7	235 ±5	260 ±6	284 ±5	5.14	272	0.30

The fitting algorithm for a single fit all vary is shown below; a modified version was used for fixing individual values and for global fitting:

```
function fitter_r1

data=[x x x x x];
fields=[4.7 9.4 11.7 14.1 16.5];
guess=[x1 x2 x3 x4];
ls_err(guess,fields,data)
options=optimset('Display','iter');
answer=fminsearch(@(x)ls_err(x,fields,data),guess,options)
hold on
plot(fields,data,'ro');
field_grid=linspace(4.7,16.5,100);
rates=zeros(size(field_grid));
for n=1:numel(field_grid)
rates(n)=R1(answer(1),answer(2),field_grid(n),answer(3),answer(4))
end
plot(field_grid,rates,'r-');
end

function err=ls_err(guess,fields,data)
err=0;
for n=1:numel(fields)
err=err+(data(n)-R1(guess(1),guess(2),fields(n),guess(3),guess(4)))^2;
end
end

function R1=R1(mu_eff,r,B,tau_r,tau_e)
mu0=4*pi*1e-7;
r=r*1e-10;
mu_eff=mu_eff*9.274e-24;
tau_r=tau_r*1e-12;
tau_e=tau_e*1e-12;
gamma_F=2.5181e8;
gamma_e=1.760860e11;
k=1.3806503e-23;
T=295;
omega_F=gamma_F*B;
omega_e=gamma_e*B;
tau_rpe=1/(1/tau_r+1/tau_e);
R1=(2/15)*((mu0/(4*pi))^2)*((gamma_F^2)*(mu_eff^2)/(r^6))*(7*tau_rpe/(1+(omega_e^2)*(tau_rp
e^2))+3*tau_rpe/(1+(omega_F^2)*(tau_rpe^2)))+(2/5)*((mu0/(4*pi))^2)*((omega_F^2)*(mu_eff^4)/
(((3*k*T)^2)*(r^6)))*(3*tau_r/(1+(omega_F^2)*(tau_r^2)));
end;
```

5. References

(1) Nocton, G.; Nonat, A.; Gateau, C.; Mazzanti, M. *Helv. Chim. Acta* **2009**, *92*, 2257–2273.

(2) Broan, C. J.; Cole, E.; Jankowski, K. J.; Parker, D.; Pulukkody, K.; Boyce, B. A.; Beeley, N. R. A.; Millar, K.; Millican, A. T. *Synthesis* **1992**, *1992*, 63–68.

(3) Walton, J. W.; Carr, R.; Evans, N. H.; Funk, A. M.; Kenwright, A. M.; Parker, D.; Yufit, D. S.; Botta, M.; De Pinto, S.; Wong, K.-L. *Inorg. Chem.* **2012**, *51*, 8042–8056.

(4) Neil, E. R.; Funk, A. M.; Yufit, D. S.; Parker, D. *Dalton Trans* **2014**, 5490–5504.

(5) Dickins, R. S.; Howard, J. A. K.; Maupin, C. L.; Moloney, J. M.; Parker, D.; Riehl, J. P.; Siligardi, G.; Williams, J. A. G. *Chem Eur J* **1999**, *5*, 1095–1105.

(6) Harvey, P. Paramagnetic Probes for Magnetic Resonance. Doctoral, Durham University, 2013.

(7) Harvey, P.; Blamire, A. M.; Wilson, J. I.; Finney, K.-L. N. A.; Funk, A. M.; Senanayake, P. K.; Parker, D. *Chem Sci* **2013**, *4*, 4251–4258.

(8) Aime, S.; Botta, M.; Garda, Z.; Kucera, B. E.; Tircso, G.; Young, V. G.; Woods, M. *Inorg. Chem.* **2011**, *50*, 7955–7965.

(9) Woods, M.; Aime, S.; Botta, M.; Howard, J. A. K.; Moloney, J. M.; Navet, M.; Parker, D.; Port, M.; Rousseaux, O. *J. Am. Chem. Soc.* **2000**, *122*, 9781–9792.

(10) Bertini, I.; Luchinat, C.; Parigi, G. *Solution NMR of Paramagnetic Molecules*; Current Methods in Inorganic Chemistry; Elsevier Science B.V.: Amsterdam, 2001; Vol. 2.

(11) Funk, A. M.; Fries, P. H.; Harvey, P.; Kenwright, A. M.; Parker, D. *J Phys Chem A* **2013**, *117*, 905–917.

(12) Chalmers, K. H.; De Luca, E.; Hogg, N. H. M.; Kenwright, A. M.; Kuprov, I.; Parker, D.; Botta, M.; Wilson, J. I.; Blamire, A. M. *Chem Eur J* **2010**, *16*, 134–148.

(13) Motulsky, H.; Christopoulos, A. *Fitting Models to Biological Data Using Linear and Nonlinear Regression: A Practical Guide to Curve Fitting*; Oxford University Press, 2004.

(14) Perrin, C. L. *J. Magn. Reson. 1969* **1980**, *40*, 391–395.

(15) Kline, M.; Cheatham, S. *Magn. Reson. Chem.* **2003**, *41*, 307–314.

(16) Reilley, C. N.; Good, B. W.; Allendoerfer, R. D. *Anal. Chem.* **1976**, *48*, 1446–1458.

(17) Reuben, J.; Elgavish, G. A. *J Magn Reson* **1980**, *39*, 421–430.

(18) Piguet, C.; Geraldes, C. F. G. C. In *Handbook on the Physics and Chemistry of Rare Earths*; Karl A. Gschneidner, J. and L. E.; Pecharsky, V. K., Eds.; Elsevier, 2003; Vol. Volume 33, pp. 353–463.

(19) Ren, J.; Zhang, S.; Dean Sherry, A.; Geraldes, C. F. G. C. *Inorganica Chim. Acta* **2002**, *339*, 273–282.

(20) S. Dickins, R.; Parker, D.; I. Bruce, J.; J. Tozer, D. *J Chem Soc Dalton Trans* **2003**, 1264–1271.

(21) Di Pietro, S.; Piano, S. L.; Di Bari, L. *Coord. Chem. Rev.* **2011**, *255*, 2810–2820.

(22) Hekmatyar, S. K.; Hopewell, P.; Pakin, S. K.; Babsky, A.; Bansal, N. *Magn Reson Med* **2005**, *53*, 294–303.
